# Low-Temperature Aqueous Alteration of Chondrites

**DOI:** 10.1007/s11214-024-01132-8

**Published:** 2025-02-04

**Authors:** Martin R. Lee, Conel M. O’D. Alexander, Addi Bischoff, Adrian J. Brearley, Elena Dobrică, Wataru Fujiya, Corentin Le Guillou, Ashley J. King, Elishevah van Kooten, Alexander N. Krot, Jan Leitner, Yves Marrocchi, Markus Patzek, Michail I. Petaev, Laurette Piani, Olga Pravdivtseva, Laurent Remusat, Myriam Telus, Akira Tsuchiyama, Lionel G. Vacher

**Affiliations:** 1 School of Geographical & Earth Sciences, University of Glasgow, Glasgow, G12 8QQ UK; 2 Earth & Planets Laboratory, Carnegie Institution of Washington, 5241 Broad Branch Road NW, Washington, DC 20015 USA; 3 Institut für Planetologie, University of Münster, Wilhelm-Klemm-Str. 10, D-48149 Münster, Germany; 4 Department of Earth & Planetary Sciences, University of New Mexico, Albuquerque, NM USA; 5 Hawai‘i Institute of Geophysics & Planetology, The University of Hawai‘i at Mānoa, Honolulu, HI 96822 USA; 6 Faculty of Science, Ibaraki University, 2-1-1 Bunkyo, Mito, Ibaraki 310-8512 Japan; 7 Université de Lille, CNRS, INRAE, Centrale Lille, UMR 8207-UMET-Unité Matériaux et Transformations, F-59000 Lille, France; 8 Planetary Materials Group, Department of Earth Sciences, Natural History Museum, Cromwell Road, London, SW7 5BD UK; 9 Centre for Star and Planet Formation, Globe Institute, University of Copenhagen, DK-1350 Copenhagen, Denmark; 10 Institute of Earth Sciences, Heidelberg University, Im Neuenheimer Feld 234-236, D-69120 Heidelberg, Germany; 11 Particle Chemistry Department, Max Planck Institute for Chemistry, Hahn-Meitner-Weg 1, 55128 Mainz, Germany; 12 Université de Lorraine, CNRS, CRPG, UMR 7358, Nancy, France; 13 Department of Earth and Planetary Sciences, Harvard University, Cambridge, 02138, USA; 14 Physics Department, Washington University, St. Louis, MO 63130 USA; 15 CNRS–Museum National d’Histoire Naturelle, Laboratoire de Minéralogie et Cosmochimie du Museum–UMR 7202, Case 52, 57 rue Cuvier, 75231 Paris Cedex 05, France; 16 Earth and Planetary Sciences, University of California Santa Cruz, Santa Cruz, CA 95064 USA; 17 Research Organization of Science & Technology, Ritsumeikan University, Shiga, 525-8577 Japan; 18 Chinese Academy of Sciences (CAS) Key Laboratory of Mineralogy & Metallogeny/Guangdong Provincial Key Laboratory of Mineral Physics and Materials, Guangzhou Institute of Geochemistry, CAS, Guangzhou, 510640 China; 19 CAS Center for Excellence in Deep Earth Science, Guangzhou, 510640 China; 20 University Grenoble Alpes, CNRS, IPAG, 38000 Grenoble, France

**Keywords:** Carbonaceous chondrites, Aqueous alteration, Clasts, Presolar grains, Organic matter, Physicochemical modelling, Hydrothermal experiments, Fluid inclusions, Stable isotopes, ^53^Mn-^53^Cr chronology, ^129^I-^129^Xe chronology

## Abstract

Chondritic meteorites (chondrites) contain evidence for the interaction of liquid water with the interiors of small bodies early in Solar System history. Here we review the processes, products and timings of the low-temperature aqueous alteration reactions in CR, CM, CI and ungrouped carbonaceous chondrites, the asteroids Ryugu and Bennu, and hydrated dark clasts in different types of meteorites. We first consider the nature of chondritic lithologies and the insights that they provide into alteration conditions, subdivided by the mineralogy and petrology of hydrated chondrites, the mineralogy of hydrated dark clasts, the effects of alteration on presolar grains, and the evolution of organic matter. We then describe the properties of the aqueous fluids and how they reacted with accreted material as revealed by physicochemical modelling and hydrothermal experiments, the analysis of fluid inclusions in aqueously formed minerals, and isotope tracers. Lastly, we outline the chronology of aqueous alteration reactions as determined using the ^53^Mn-^53^Cr and ^129^I-^129^Xe systems.

**Supplementary Information:**

The online version contains supplementary material available at 10.1007/s11214-024-01132-8.

## Introduction

The radiogenic heating of small bodies early in Solar System history produced liquid water from the melting of accreted water-rich ices. The principal products of the reaction of water with other accreted materials were phyllosilicate minerals whose abundance helps to classify such altered carbonaceous chondrites into petrologic types 1 and 2. Samples of these hydrated lithologies are available as the CR (Renazzo-like), CM (Mighei-like), and CI (Ivuna-like) meteorites, ungrouped carbonaceous chondrites, clasts that occur in a wide variety of meteorite groups, and grains returned from the Cb-type asteroid Ryugu and B-type asteroid Bennu. Hydrated lithologies include interplanetary dust particles and Antarctic micrometeorites (e.g., Bradley 2014), but they are not covered here.

This article starts by describing the petrological and mineralogical characteristics of meteorites and clasts that have undergone low-temperature aqueous alteration, and the insights that their properties can provide into the physicochemical conditions and environments of aqueous alteration. The effects of liquid water on two of the originally accreted constituents of carbonaceous chondrite parent bodies, presolar grains and organic matter, is then described. Next, this article explores the properties of the aqueous fluids, and how they reacted with the chondritic parent bodies (e.g., chemistry, temperature, water-rock (W/R) ratio, duration of water-rock interaction) as investigated by physicochemical modelling and hydrothermal experiments, by analyzing samples of water preserved as fluid inclusions, and through measurement of the oxygen, hydrogen, carbon, nitrogen and sulfur isotope compositions of bulk rocks and constituent minerals. This article finishes by describing the chronology of aqueous alteration as revealed by the ^53^Mn-^53^Cr and ^129^I-^129^Xe systems.

## Mineralogy and Petrology of Hydrated Carbonaceous Chondrites

This section includes the three groups of hydrated chondrites (CR, CM, CI), several of the more well-studied ungrouped carbonaceous chondrites (Tagish Lake, Tarda, Flensburg), and samples that have been recently returned from asteroids Ryugu and Bennu. It finishes with a description of thermally altered carbonaceous chondrites.

### CR (Renazzo-Like) Chondrites

The CR chondrites are characterised by high abundances of presolar silicate grains (except heavily hydrated CRs), very elevated D/H ratios of their organic matter, and a solar Co/Ni ratio of Fe-Ni metal (Kallemeyn and Wasson 1981; Krot et al. 2002; Floss and Haenecour 2016). This record is preserved despite the CR chondrites exhibiting significant variations in their degree of alteration, but show no evidence of thermal metamorphism, with the exception of the shock-heated CR Graves Nunataks (GRA) 06100 (Abreu and Bullock 2013). The vast majority of CRs are Saharan and Antarctic finds. Only two falls are known: Al Rais and Renazzo (potentially three if Bells is considered a CR; Marrocchi et al. 2023a). Although most CRs are predominantly composed of chondrules and have a low modal abundance of matrix (\Math@Op@BoldSymbol70 *vs* \Math@Op@BoldSymbol30 vol. %), rare matrix-rich CR chondrites like Al Rais also exist (∼70 vol. % matrix) (Schrader et al. 2011). Like CM chondrites, CRs are commonly brecciated and contain so-called dark inclusions that have been widely documented, as well as clasts of more highly altered lithologies (Endress et al. 1994).

With the exception of the almost completely altered CR1 chondrites Al Rais and Grosvenor Mountains (GRO) 95577 (Weisberg and Huber 2007; Schrader et al. 2011), CRs typically belong to petrologic type 2 (Schrader et al. 2011; Harju et al. 2014). However, this classification is more nuanced because some CR chondrites, such as Queen Alexandra Range (QUE) 99177, Meteorite Hills (MET) 00426 and Miller Range (MIL) 090657, exhibit characteristics more typical of type 3.00 chondrites (e.g., Abreu and Brearley 2010). CRs can be classified as type 2 (e.g., Harju et al. 2014; Howard et al. 2015), but have experienced significantly less alteration than almost all CM2 chondrites. Many CR chondrites have minimally altered glassy chondrule mesostasis and Fe-Ni metal beads in chondrules (Weisberg et al. 1993; Abreu and Brearley 2010) as well as high abundances of presolar silicate grains that are known to be highly sensitive to aqueous alteration (e.g., Floss and Stadermann 2009; Floss and Haenecour 2016) (Sect. 4). The generally lower degrees of alteration of the CRs are reflected in their low bulk H contents (0.14–0.68 wt.% H) compared to CM2 chondrites (0.9–1.5 wt.% H) (Alexander et al. 2012). The low degree of aqueous alteration makes the CR chondrites of special importance for studying the earliest interaction of aqueous fluids with chondritic materials. Some CRs, such as Renazzo, GRO 95577 and Al Rais, show more advanced alteration with glassy chondrule mesostases being completely replaced by phyllosilicates (Weisberg et al. 1993).

**Fig. 1:**
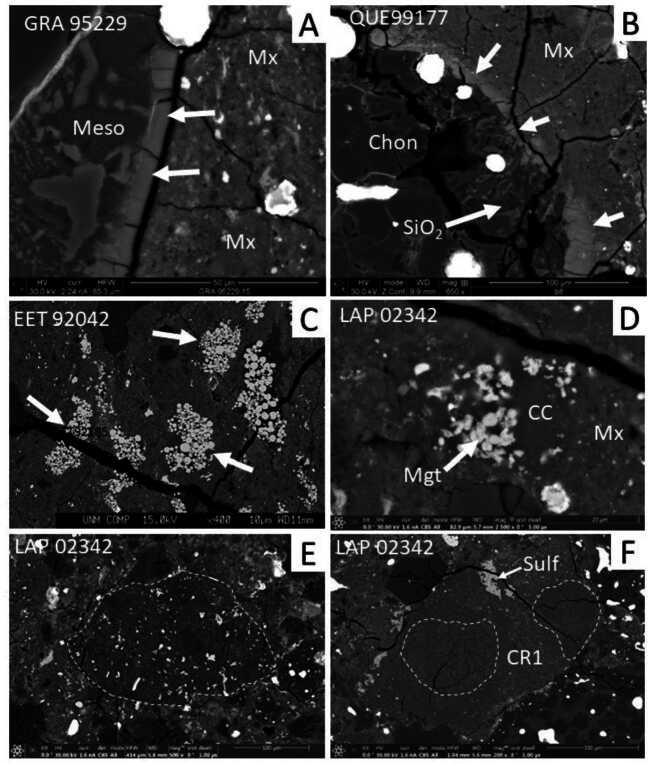
Backscattered electron (BSE) images of alteration features and clasts in CR chondrites. (A) Thin alteration rind (indicated by white arrows) on the edge of a type IIA chondrule in GRA 95229. The alteration rind has replaced mesostasis glass where it is in direct contact with the matrix (Mx). In this example, the alteration rind is <5 μm in thickness and is enriched in FeO compared with the adjacent unaltered mesostasis (Meso). (B) Smooth rim associated with a silica-rich igneous rim (SIR) in a type IA porphyritic olivine chondrule in QUE 99177. The smooth rim (indicated by white arrows and with slightly higher BSE contrast) has replaced abundant silica crystals (SiO_2_) in the SIR which is also in contact with the adjacent fine-grained matrix (Mx). (C) Abundant, large magnetite framboids (indicated by white arrows) in a region of matrix in the Elephant Moraine (EET) 92042 CR chondrite. (D) Calcite grain (CC) intergrown with extremely fine-grained framboidal magnetite (Mgt) within the matrix of LaPaz Icefield (LAP) 02342. (E) Example of a dark clast (optically dark, but a distinct lithic clast with very few or no anhydrous silicates; outlined by white dashed line) of a C1 lithology consisting of a very fine-grained matrix, with coarse-grained magnetite and sulfides in LAP 02342 (compare Sect. 3). (F) A CR1 clast within LAP 02342 (CR2.8 – Harju et al. 2014). The clast consists of parts of completely pseudomorphed chondrules (outlined by white dashed line) that have been replaced by serpentine with a uniformly low contrast and large masses of Fe,Ni sulfide (Sulf)

For most CR chondrites, aqueous alteration effects are seen mainly in the fine-grained matrix that occurs interstitially to chondrules; fine-grained matrix-like chondrule rims are comparatively rare in CR chondrites. Scanning electron microscope (SEM) studies show that the dominant secondary alteration minerals that form in the matrix are framboidal magnetites [$\text{Fe}^{2+}\text{Fe}^{3+}{}_{2}\text{O}_{4}$] and Ca carbonates (Fig. 1) with grain sizes of 10s of microns. The abundance of these minerals increases with increasing degree of aqueous alteration (Harju et al. 2014). Transmission electron microscope (TEM) studies of several different CR chondrite matrices (e.g., Abreu and Brearley 2010; Le Guillou and Brearley 2014; Le Guillou et al. 2015a; Changela et al. 2018; Davidson et al. 2019; Vollmer et al. 2020a) show that the least altered CRs contain matrix that is dominated by Fe-rich amorphous silicate material that contains abundant nanophase Fe,Ni sulfides and is complexly intergrown with insoluble organic matter (Le Guillou and Brearley 2014; Floss et al. 2014). The amorphous silicate material is both hydrated (Bonal et al. 2013; Le Guillou and Brearley 2014) and highly oxidized (Le Guillou et al. 2015a). As aqueous alteration of matrix advances, either due to an increase in temperature or increased availability of aqueous fluid, the amorphous silicate material progressively transforms to fine-grained hydrous phyllosilicates (an intergrowth of serpentine [(Mg,Fe)_3_Si_2_O_5_(OH)_4_] and smectite (saponite) [$\text{(Ca,Na)}_{0.3}(\text{Mg,Fe}^{2+})_{3}(\text{Si,Al})_{4}\text{O}_{10}\text{(OH)}_{2}\cdot 4\text{H}_{2}\text{O}$]). The increase in phyllosilicate abundance with increasing aqueous alteration has been tracked by both X-ray diffraction (XRD) (Howard et al. 2015) and TEM studies (Abreu and Brearley 2010; Le Guillou and Brearley 2014; Changela et al. 2018; Vollmer et al. 2020a). Generally, alteration mostly affected the matrices of CR chondrites forming mineral assemblages that are similar to those of CI chondrites, although more limited in extent. The effects of aqueous alteration on matrix are also tracked by the abundance of presolar silicate grains (Sect. 4).

Petrographic evidence for alteration of chondrules in minimally altered CR chondrites is limited to the replacement of chondrule mesostasis glass in narrow zones a few 10s to a 100 μm wide in contact with matrix. A unique feature of the alteration in CR chondrites, which has not been observed in any other chondrite group, is the presence of so-called smooth rims (Harju et al. 2014; Martínez and Brearley 2022), 5–50 μm in thickness, surrounding some chondrules (Fig. 1B). The smooth rims are present in even the least altered CR chondrites such as QUE 99177 and MET 00426. Martínez and Brearley (2022) showed that smooth rims in QUE 99177 formed by the replacement of silica (cristobalite, SiO_2_) within Silica-Rich Igneous Rims (SIRs) that occur surrounding some type I chondrules (Fig. 1B) (Noguchi 1995; Krot et al. 2004). Smooth rims consist of a very Fe-rich amorphous silicate phase (Brearley and Jones 2016; Martínez and Brearley 2022) and show no evidence for phyllosilicates. Secondary phases in CR2 chondrites include framboidal magnetite, Ca carbonates, fine-grained sulfides and phyllosilicates corresponding to intergrowths between dominant serpentine and saponite (Weisberg et al. 1993; Zolensky et al. 1993) (Fig. 1C, D). During more advanced alteration, dolomite [CaMg(CO_3_)_2_] also forms, and in the CR1 chondrites siderite [FeCO_3_] can be present.

Isotopic data are very informative about the evolution of CC lithologies. Bulk O-isotope studies of a large number of CR chondrites (mostly Antarctic finds) (Schrader et al. 2011) show a progressive increase in $\delta ^{17}\text{O}$ and $\delta ^{18}\text{O}$ that appears to correlate well with petrographic evidence of aqueous alteration, such as the abundance of phyllosilicates $\delta ^{18}\text{O} =$ [(^18^O/^16^O)_sample_/(^18^O/^16^O)_standard_ −1] × 1000, $\delta$^17^O = [(^17^O/^16^O)_sample_/(^17^O/^16^O)_standard_ −1] × 1000. The bulk O-isotope compositions lie on a well-defined linear array on a three O-isotope plot with a slope of 0.70, with QUE 99177 defining the lightest O-isotope composition of ∼−4‰ and $\delta^{18}\text{O}=∼-2.3‰$ and Al Rais the heaviest ($\delta^{17}\text{O}=∼4.3‰$ and $\delta^{18}\text{O}=\;∼10.8‰$). $\Delta ^{17}\text{O}$ ($= \delta ^{17}\text{O} - 0.52 \times \delta ^{18}\text{O}$), a measure of deviation from the terrestrial fractionation line (TFL), ranges from ${\sim} {-}3$ to −0.4‰ (Sect. 8). These data demonstrate that most CR chondrites have relatively low degrees of aqueous alteration, consistent with their petrographic characteristics, and that the two known CR chondrite falls, Renazzo and Al Rais, are among the most heavily altered CR chondrites. It is estimated that the CRs had W/R mass ratios of 0.10–0.40 (Sect. 8.1.4).

The O-isotope composition of secondary minerals (Jilly-Rehak et al. 2018) provides constraints on the conditions of aqueous alteration in CR chondrites. Carbonates and magnetite show a range of $\delta ^{18}\text{O} \approx 9$ to 35‰ in calcite [CaCO_3_, trigonal], from $\delta ^{18}\text{O}\approx 23$ to 27‰ in dolomite, and from $\delta ^{18}\text{O}\approx -18$ to 5‰ in magnetite. The widest variation in O-isotope compositions occurs in the minimally altered CR chondrites such as QUE 99177 and MIL 090292, which Jilly-Rehak et al. (2018) proposed is due to low W/R ratios and progressive evolution in the isotopic compositions of the fluid. In this scenario, carbonate grains grew at different times and reflect this isotopic evolution that may also be caused by local variations in fluid composition. In more heavily altered CRs, e.g., Al Rais and the CR1 GRO 95577, the carbonate O-isotope compositions have a more restricted range, suggesting that they all formed from the same isotopically homogeneous fluid. Assuming closed-system aqueous alteration on the CR chondrite parent body, and that isotopic equilibrium was attained between carbonate and magnetite, Jilly-Rehak et al. (2018) calculated a global temperature of aqueous alteration from ∼55 to 88 °C, based on the parallel arrays of calcite and magnetite O-isotope compositions, and Marrocchi et al. (2018) estimated temperatures of between 35 and 110 °C (Sect. 8.1.4). The chronology of aqueous alteration of the CRs is discussed along with other CC groups in Sect. 9.

### CM (Mighei-Like) Chondrites

**Fig. 2:**
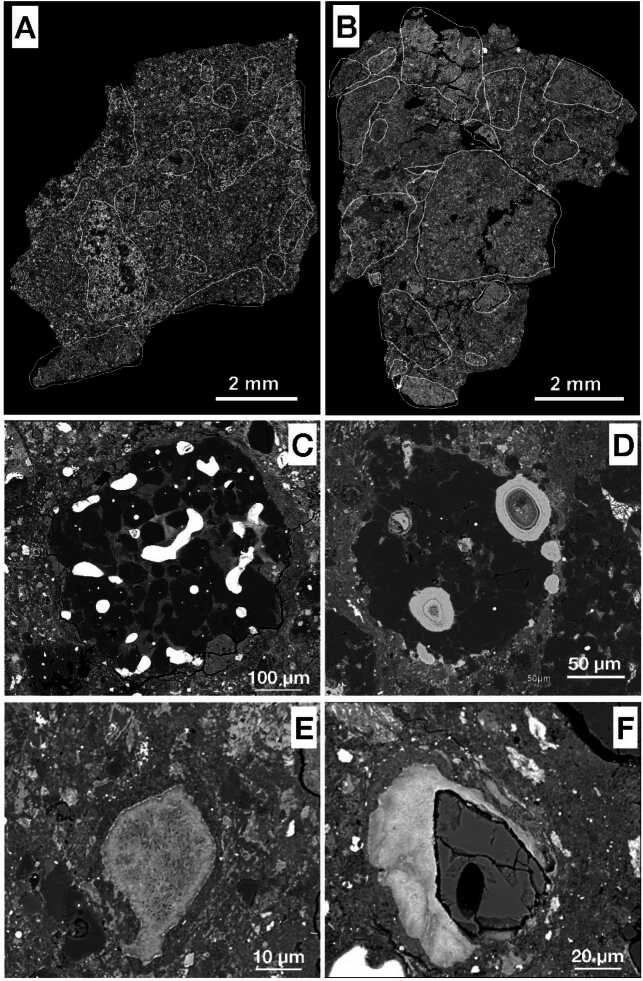
BSE images of CM meteorites. (A) and (B) are the CM breccias ALH 85013 and LON 94101 (sample: co. 36), respectively. Fragments with different degrees of alteration are embedded in a fine-grained clastic matrix. From Lentfort et al. (2021). (C) A type I chondrule in the Paris that contains well-preserved metal beads but altered mesostasis. (D) Altered Fe-Ni metal beads within a type I chondrule of Murchison. (E) A tochilinite-cronstedtite intergrowth (TCI) in Paris. (F) BSE image of Ca-carbonate (CaCO_3_) surrounded by TCIs within the matrix of Paris. Black spot corresponds to a SIMS pit

Among the carbonaceous chondrites, the CM chondrites are the most abundant group, representing around 30% of falls and finds. CM-like matter also constitutes an important fraction of xenoliths discovered in meteorites (Gounelle et al. 2003; Patzek et al. 2018; Marrocchi et al. 2021a; King et al. 2022) (Sect. 3), attesting to the importance of these types of meteorites in the formation and the evolution of the Solar System. CM chondrites are dark, highly brecciated meteorites, in which lithic clasts show various degrees of aqueous alteration (Fig. 2, e.g., Metzler et al. 1992; Bischoff et al. 2006, 2017; Lindgren et al. 2013; Verdier-Paoletti et al. 2019; Lentfort et al. 2021; Kerraouch et al. 2022; Suttle et al. 2022a). The main components of CMs are abundant fine-grained matrix (∼70 vol. %), small chondrules (0.194 mm; Floyd et al. 2024) and refractory inclusions, and abundant secondary minerals. Most chondrules are surrounded by fine-grained rims (FGRs; Metzler et al. 1992).

The CM chondrites represent the largest group of hydrated meteorites with water contents ranging from 3 to almost 10 wt.% (Vacher et al. 2020; Marrocchi et al. 2023b). They span a wide range of alteration degrees, from less altered (i.e., CM2) down to heavily altered (i.e., CM1; Zolensky et al. 1997), with several minimally altered samples being recognized over the last decade (Fig. 2, Hewins et al. 2014; Marrocchi et al. 2014; Kimura et al. 2020). These mildly altered CMs are characterized by well-preserved Fe-Ni metal beads and glassy chondrule mesostases as well as porous aggregates of amorphous silicates dispersed throughout the fine-grained matrix (Leroux et al. 2015; Kimura et al. 2020). More altered CMs contain a variety of secondary phases produced through aqueous alteration processes, including carbonates, sulfides, oxides, and hydroxides (Fig. 2, Brearley 2006). The most characteristic secondary phases in CM chondrites are tochilinite-cronstedtite intergrowths (TCIs, Fig. 2E), occurring as complex assemblages dispersed throughout the chondrules, FGRs, and matrix (Mackinnon and Zolensky 1984; Pignatelli et al. 2017). Tochilinite is a mineral with an incommensurate structure based on two layers: (i) a mackinawite-type Fe_1−x_S sulfide layer and (ii) a brucite- or amakinite-type hydroxide layer, depending on the composition. Its ideal formula is 2(Fe_1−x_S)n(Fe,Mg,Al,Ca)(OH)_2_ with 1.58 < n < 1.85 (Zolensky and MacKinnon 1986) and 0.08 < x < 0.28 (Gubaidulina et al. 2007), where n and x correspond to the hydroxide layer content and the vacancies of the sulfide layer, respectively. Cronstedtite is a T-O phyllosilicate with a general formula $(\text{Fe}^{2+}_{3-\mathrm{x}}\text{Fe}^{3+}_{\mathrm{x}})(\text{Si}_{2-\mathrm{x}}\text{Fe}^{3+}_{\mathrm{x}})\text{O}_{5}\text{(OH)}_{4}$ with 0 < x < 0.8 in terrestrial crystals (Pignatelli et al. 2016, 2017).

The majority of CM2s are dominated by phyllosilicates in the range 73–79 vol. % (average = 75 vol. %) with Fe-cronstedtite (43–50 vol. %) being more abundant than Mg-serpentine (25–33 vol. %) (Howard et al. 2015). The only exception is Cold Bokkeveld where the abundance of Mg-serpentine (49–59 vol. %) exceeds that of Fe-cronstedtite (19–27 vol. %). For fall samples there is a limited range in olivine (6.9 vol. %) and pyroxene (5 vol. %) whereas find samples show larger anhydrous silicate abundances that can reach up to 30 vol %. Modal abundances of the remaining identified phases also show a limited range: calcite (0–1.3 vol. %); gypsum [CaSO_4_.2H_2_O] (0–1.6 vol. %, but likely terrestrial contamination); magnetite (1.1–2.4 vol. %); pentlandite [(Fe,Ni)_9_S_8_] (0–2.1 vol. %) and pyrrhotite [Fe_1-x_S] (1–3.8 vol. %). More altered samples (CM1) contain 6–10 vol. % olivine + pyroxene and 86–88 vol. % total phyllosilicate. Magnetite (0.6–5.2 vol. %), sulfide (0.6–3.9 vol. %), calcite (0–1.9 vol. %) and gypsum (0–0.8 vol. %) are minor phases across all samples.

Calcite is present in all CMs and is often the most abundant carbonate mineral. Aragonite [CaCO_3_, orthorhombic] also occurs in the less altered meteorites (Barber 1981; Lee and Ellen 2008; Lee et al. 2014) and dolomite in the more highly altered ones (Rubin et al. 2007; de Leuw et al. 2010; Lee et al. 2014). Breunnerite [(Fe,Mg)CO_3_], and ankerite [Ca(Mg,Fe,Mn)(CO_3_)_2_] have been described only from the highly altered QUE 93005 (Lee et al. 2012). Several generations of calcite have been recognised from their petrographic characteristics and O-isotope compositions. Tyra et al. (2012) defined Types 1 and 2, with Vacher et al. (2018) adding Type 0, and subdividing Types 1 and 2 into two subtypes. The type 1 calcite grains often display cathodoluminescence (CL) zoning that reflects changes in the concentrations of Mn and Fe of the precipitating fluids during crystal growth. Fujiya et al. (2020) showed that the O-isotope composition of calcite can also differ significantly between the zones of individual crystals in Yamato-791198 such that later zones have a lower $\delta ^{18}\text{O}$ and $\Delta ^{17}\text{O}$ than earlier ones. These isotopic differences reflect the evolution of the O-isotope composition of aqueous solutions during their progressive reaction with anhydrous silicates and show that the growth rate of calcite crystals was slower than the rate of isotopic changes in parent body fluids.

The liquid water that was responsible for aqueous alteration of the CM chondrites came from the melting of accreted ices. The main source of heat was the decay of ^26^Al, although collisions may also have contributed (Rubin 2012; Vacher et al. 2018). The temperatures of the fluids responsible for alteration have been investigated extensively using a range of geothermometers including clumped O-isotopes (∼20–71 °C, Guo and Eiler 2007; ∼5–101 °C, Clog et al. 2024), phyllosilicate-carbonate O-isotope fractionation (<∼20 °C, Clayton and Mayeda 1984), magnetite-carbonate O-isotope fractionation (125 ± 60 °C, Telus et al. 2019), and modeling of carbonate O-isotope compositions (0–130 °C, Alexander et al. 2015; 113 ± 54 °C, Verdier-Paoletti et al. 2017, 2019, Vacher et al. 2019a). These measurements typically provide only one temperature per meteorite, whereas the CM parent bodies will have undergone at least one heating-cooling cycle, and possibly several considering the occurrence of carbonates in different clasts with various degrees of alteration within the same CM breccia and if collisions generated thermal pulses. The aqueous alteration products could have formed during heating and/or cooling, and Vacher et al. (2019b) have proposed that carbonates crystallized early during heating whereas tochilinite formed shortly before or after the parent body reached its peak temperature. The duration of aqueous alteration of any one CM is difficult to quantify, although Velbel et al. (2012) estimates that serpentinization of chondrule olivine would need timescales of hundreds of days to more than a decade. The chronology of aqueous alteration is discussed in Sect. 9.

### CI (Ivuna-Like) Chondrites

**Fig. 3:**
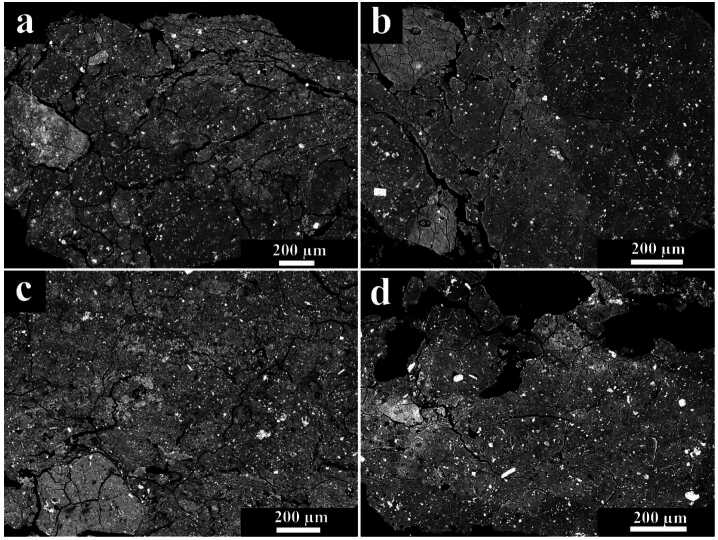
Representative BSE images of typical textures of CI chondrites, demonstrating brecciation. (a) Orgueil, (b) Ivuna, (c) Alais, and (d) Tonk. The white spots are magnetites and pyrrhotite typically embedded within a fine-grained intergrowth of phyllosilicates. The degree of brecciation decreases in the sequence: Orgueil (a) > Ivuna (b) > Alais (c) ∼ Tonk (d). From Alfing et al. (2019)

The rare CI carbonaceous chondrites are all classified as petrologic type 1 having experienced pervasive aqueous alteration. They have primitive bulk chemical compositions that are almost identical to the solar photosphere, making them of significant cosmochemical importance (e.g., Anders and Grevesse 1989). The main component is an abundant (>90%) fine-grained (<1 μm) matrix of phyllosilicates (e.g., Tomeoka and Buseck 1988). The phyllosilicates are Fe-Mg serpentine-group minerals interlayered with saponite (smectite-group). Other phases include oxides (magnetite, ∼3–6 vol. %), Fe-sulfides (pyrrhotite, pentlandite, cubanite [CuFe_2_S_3_], ∼1–7 vol. %), carbonates (dolomite, breunnerite, calcite, ∼0.1–2 vol. %), and phosphates (Alfing et al. 2019) (Fig. 3). The scarcity of intact CAIs and chondrules implies the parent body either accreted in a region of the protoplanetary disk nearly lacking them, or that the primary mineralogy and textures were destroyed by aqueous alteration (Frank et al. 2023).

The CI chondrites were altered at temperatures of ∼50 to >150 °C, W/R mass ratios of 0.38–0.43, and a pH and Eh of ∼7–10 and ${\sim} \ -0.2$ to −0.8, respectively (e.g., Zolensky et al. 1989). These conditions are largely supported by isotopic and petrographic constraints (e.g., Clayton and Mayeda 1999). Other minerals commonly reported in the CI chondrites are sulphates (e.g., gypsum [$\text{CaSO}_{4}\boldsymbol{\cdot} 2\text{H}_{2}\text{O}$], epsomite [$\text{MgSO}_{4}\boldsymbol{\cdot}7\text{H}_{2}\text{O}$], and bloedite [$\text{Na}_{2}\text{Mg}(\text{SO}_{4})_{2}\boldsymbol{\cdot}4\text{H}_{2}\text{O}$]) and ferrihydrite [$\text{Fe}^{3+}_{10}\text{O}_{14}\text{(OH)}_{2}$] (Gounelle and Zolensky 2001). The sulphates occur as white flecks, flakes, and efflorescence on sample surfaces and infilling veins within the interior of the meteorites, while the ferrihydrite is intimately intergrown with phyllosilicates. The porous and volatile-rich nature of CI chondrites makes them susceptible to terrestrial modification, and the sulphates and ferrihydrite probably formed from reactions between the meteorites and atmospheric O_2_ and H_2_O (Gounelle and Zolensky 2001).

### (162173) Ryugu

The mineralogy, petrography, and chemical properties of samples returned from the Cb-type asteroid (162173) Ryugu are almost identical to the CI chondrites (Yokoyama et al. 2023; Nakamura et al. 2023). In hand specimen, these samples are dark, highly friable, and have bulk densities up to ${\sim} 1.8\text{ g cm}^{-3}$. Most of the examined particles are breccias, but typically they consist of an abundant (∼90%) fine-grained (<1 μm) matrix of phyllosilicates (interlayered serpentine and saponite) in which are embedded coarser (∼10’s–100’s μm in size) grains, fragments, and clusters of oxides (∼4 vol. %), Fe-sulfides (∼3 vol. %), carbonates (∼3 vol. %), and phosphates (<1 vol. %). Sulphates and ferrihydrite are absent from pristine particles not exposed to Earth’s atmosphere, seemingly confirming the terrestrial origin of these phases in the CI chondrites. Small (<30 μm) fragments of CAI- and chondrule-like materials have been identified in some Ryugu particles (e.g., Nakamura et al. 2023).

Alteration temperatures of <∼50 °C for Ryugu particles have been estimated from the O-isotope composition of dolomite-magnetite assemblages (Yokoyama et al. 2023) and the composition and structure of Fe-sulfide grains (Nakamura et al. 2023). Thermodynamic modelling suggests a fluid Eh <−0.45 V, pH >∼8.5, and W/R ratios of 0.2–0.9 for the main highly altered lithology (Nakamura et al. 2023). Fluid inclusions trapped within pyrrhotite grains retain H_2_O and CO_2_, consistent with Ryugu’s parent body accreting beyond the water and carbon dioxide snowlines at heliocentric distances of >∼3–4 au (Nakamura et al. 2023, Sect. 7). The aqueous alteration chronology of Ryugu is discussed in Sect. 9.

### (101955) Bennu

Initial characterization of samples returned from the B-type asteroid (101955) Bennu has shown that they are dark, with morphologies that range from angular to sub-rounded or hummocky (Lauretta et al. 2024). The mineralogy is dominated by phyllosilicates (serpentine and saponite, ∼80 vol.%), with minor amounts of Fe-sulfides (∼10 vol.%), oxides (∼5 vol.%), carbonates (∼3 vol.%), and olivine (∼2 vol.%). Mg,Na-phosphates are also present, often occurring as higher reflectance coatings, veins, or individual grains within the matrix. Overall, the mineralogy and petrography of Bennu particles are consistent with extensive aqueous alteration and closely resemble samples of Ryugu and low petrologic type carbonaceous chondrites.

### Ungrouped Hydrated Carbonaceous Chondrites

#### Tagish Lake

Tagish Lake is an ungrouped C2 carbonaceous chondrite whose chemical and isotopic composition, mineralogy, and petrographic characteristics suggest affinities to both the CI and CM groups (Brown et al. 2000; Zolensky et al. 2002). The meteorite is a breccia containing multiple lithologies including carbonate-poor, carbonate-rich, magnetite- and sulfide-rich, and carbonate-rich siderite-dominated (Zolensky et al. 2002; Izawa et al. 2010). These lithologies have a fine-grained matrix composed of serpentine and saponite together with magnetite and sulfides within which are rare altered chondrules and CAIs, anhydrous silicate grains, carbonate nodules that range widely in composition (calcite-dolomite-siderite), magnetite, and Fe-Ni sulfides (Brown et al. 2000; Zolensky et al. 2002). The mineralogy and petrography of Tagish Lake shows that it is petrologic type 2 with different lithologies showing contrasting degrees of aqueous alteration under largely isochemical conditions (Greshake et al. 2005; Herd et al. 2011; Alexander et al. 2014; Blinova et al. 2014a). Despite the intensity of alteration, Blinova et al. (2014b) showed that one sample (TL5b) contains minimally aqueously altered amorphous material of a nebular origin that is analogous to amorphous material in some CR and CM chondrites (Chizmadia and Brearley 2008; Abreu and Brearley 2010). Tagish Lake has also incorporated exogeneous CM1 materials (Zolensky et al. 2002).

#### Tarda

Tarda fell in Morocco in 2020 and is classified as a C2 ungrouped chondrite. It has a bulk O-isotope composition, and enrichments in deuterium (D), ^15^N, and ^13^C that suggest a close genetic relationship to Tagish Lake (Marrocchi et al. 2021b). Tarda contains highly altered chondrules and isolated olivine grains, rare CAIs, magnetite clusters, Fe-sulfides (pyrrhotite, pentlandite, ∼8 vol. %), and carbonates (dolomite, calcite, siderite). However, the most abundant component is a fine-grained matrix of phyllosilicates (∼72 vol. %), which comprise serpentines, smectites, and an interlayered serpentine/smectite phase. The composition of Fe-sulfides in Tarda constrains its alteration temperature to ∼100–135 °C.

#### Flensburg

Flensburg is a C1 ungrouped chondrite breccia that was observed to fall in Germany in 2019 (Bischoff et al. 2021). It shares some mineralogical and chemical properties with highly altered members of the CM, CR, and CI groups, but has textures and bulk H, C, and N compositions that differ from other carbonaceous chondrites. Flensburg consists of chondrule pseudomorphs, clusters of Fe-sulfides (pyrrhotite, troilite [FeS], pentlandite, ∼4 vol. %), magnetite (∼2 vol. %), and carbonate grains (calcite, dolomite, ∼4 vol. %) within a matrix of serpentine-rich phyllosilicates (∼90 vol. %). Fe-sulfide compositions are consistent with formation at temperatures of ∼150–200 °C, and bulk O-isotope indicate that Flensburg was altered at a relatively low W/R ratio. The ^53^Mn-^53^Cr ages of Flensburg carbonates are discussed in Sect. 9.

### Aqueously and Thermally Altered Chondrites

A number of aqueously altered chondrites also experienced thermal metamorphism at temperatures of ∼300 °C to >750 °C. This heating resulted in the dehydration of phyllosilicates and crystallization of olivine and pyroxene, and the modification and depletion of volatile species (e.g., water, noble gases, and organics) (Nakamura 2005). Thermal metamorphism must have occurred after the main period of aqueous alteration since phyllosilicates are dehydrated in these meteorites, but it remains unclear whether the process was a single event or episodic. Mineral textures and the structure of organics indicate that the metamorphism was short-lived, on the order of hours to several years, implying that the heat source was impacts and/or solar radiation rather than radiogenic decay.

## Mineralogy of Hydrated Dark Clasts

### Introduction

#### What Are Dark Clasts?

**Fig. 4:**
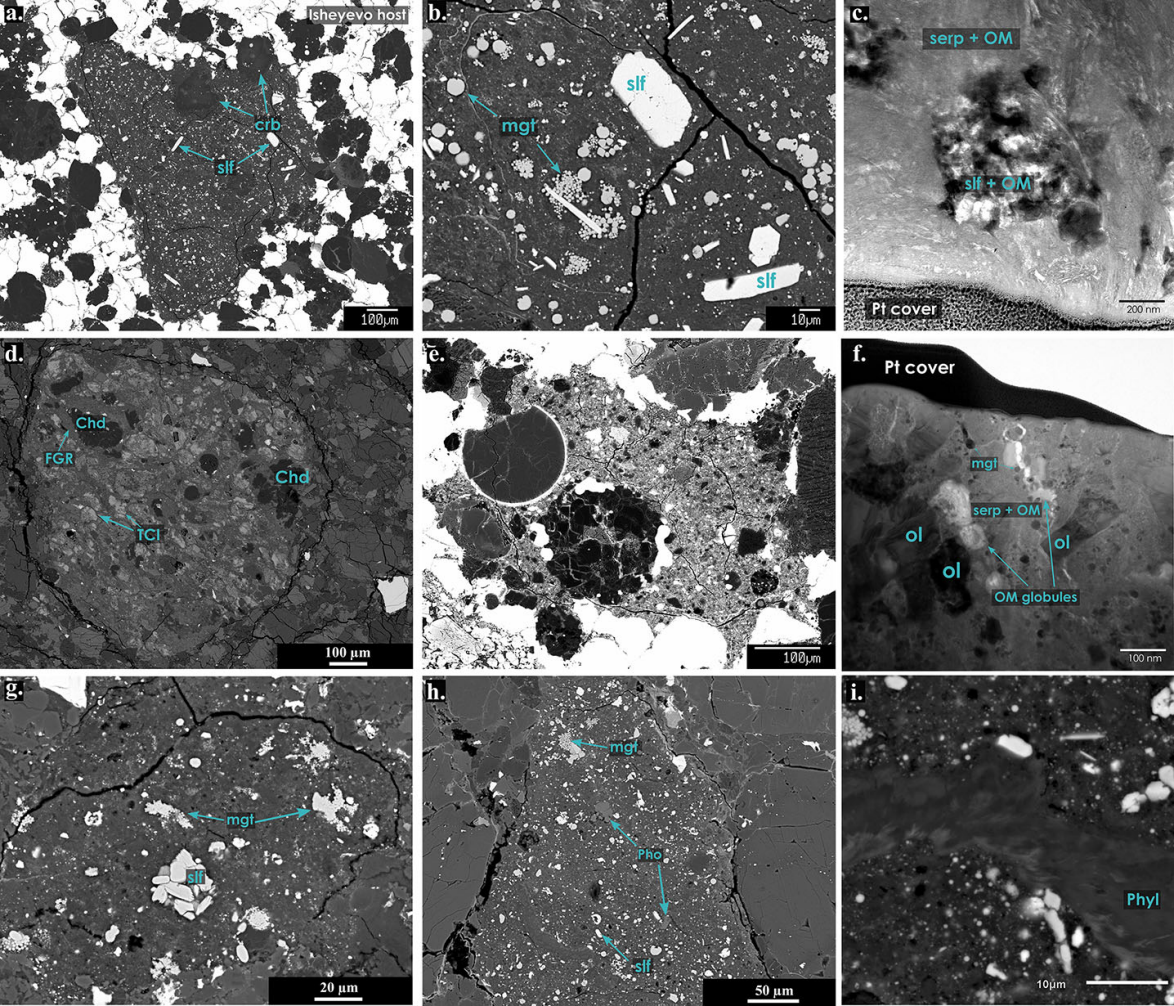
Hydrated dark clasts from various Solar System objects, including (a) C1 clast from Isheyevo (van Kooten et al. 2017a) including abundant sulfide (slf) and complex carbonates (crb) (b) zoom-in of panel A: framboidal and single magnetite (mgt) grains. (c) TEM cross-section of Isheyevo C1 clast showing a fine-grained serpentine-rich matrix (serp) with diffuse organic matter (OM) and pockets of sulfide with more granular OM (from van Kooten et al. 2017a). (d) C2 clast from polymict eucrite NWA 7542 that contains Mg-rich chondrules (Chd) and mineral fragments surrounded by fine-grained rims (FGR). Fe-rich lumps of tochilinite (TCI), sulfide and carbonate are spread throughout the clast and are embedded in a fine-grained matrix (Patzek et al. 2018). (e) C2 clast from Isheyevo with a large cryptocrystalline and another porphyritic chondrule with sulfide and metal rim, respectively. Anhydrous silicates dominate the matrix (van Kooten et al. 2017a). (f) TEM cross-section of C2 clast from Isheyevo showing globular OM, olivine (ol) grains and small magnetite grains (mgt). (g) C1 clast from ordinary H chondrite SAH 98645 with abundant magnetite (mgt) and (platy) sulfide (slf) in a phyllosilicate matrix. (h) C1 clast from polymict ureilite Dar al Gani (DaG) (Patzek et al. 2018) with a similar mineralogy as panel g, but also containing phosphates (Pho) and large phyllosilicates (phyl, panel i)

Most chondritic meteorites, but also a significant number of achondrites, are breccias (i.e., mixtures of different rock types within a single specimen). Most of these breccias likely represent rock units from a single parent body (genomict breccias), but many chondrites and achondrites also include xenolithic clasts that were derived from a different Solar System objects. The most obvious and easily visible clasts in meteorite breccias are the so-called dark inclusions or clasts. In general, most of these clasts are not chondritic in origin and the term “dark clast” does not provide any mineralogic or genetic information (Bischoff et al. 2006). Except for the carbonaceous chondrite groups and polymict ureilites, dark clasts in most brecciated meteorite groups are dominated by (1) shock-darkened objects, (2) fragments of fine-grained brecciated materials, (3) metal-troilite-rich clasts, (4) fine-grained, matrix-like cognate inclusions (related to the host rock but with a different alteration degree), and (5) fragments of shock melts with abundant tiny metal/sulfide grains (Bischoff et al. 2006). Dark clasts in this work exclusively refer to hydrated clasts in all kinds of chondritic and achondritic breccias and will be subdivided into C1- and C2-type clasts based on their degree of aqueous alteration (Fig. 4).

#### Classification of Hydrated Dark Clasts

C1 clasts represent the highest degree of hydrous modifications and include fragments of the brecciated CI-chondrite type, homogeneous fragments that could occur as clasts in CI-breccias, and C1-like fragments (not related to CI chondrites). All C1-like clasts either contain no or only very minor (<2 vol. %) anhydrous phases (e.g., olivine [(Mg,Fe)_2_SiO_4_], pyroxene, metal; Endress 1994; Morlok et al. 2006; Patzek et al. 2018; Alfing et al. 2019; Morin et al. 2022) (Fig. 4a-c). C2 clasts include typical fragments of brecciated CM-related clasts since most (if not all) CM chondrites are breccias (Fig. 4d). However, not all anhydrous mineral-bearing (or chondrule-containing) clasts are CM-related and thus, the neutral description ‘C2-type’ is more appropriate.

#### Occurrence of Hydrated Dark Clasts

Only very few reports describe the occurrence of hydrated clasts in non-carbonaceous chondrites (e.g., Zolensky et al. 2003, 2016; Funk et al. 2011; Briani et al. 2012; Greshake 2014; Bischoff et al. 2018; Patzek et al. 2018) (Fig. 4d). Phyllosilicate-rich clasts have not been found in enstatite chondrites and members of the Kakangari grouplet. The main reason that C1 or C2 clasts have not yet been found in the most reduced non-carbonaceous (NC, Warren 2011) chondrite groups might be the small number of samples among the recovered meteorites. Ordinary chondrites contain rare C1 clasts, which are most abundant in H-chondrites (e.g., Zolensky et al. 2003; Funk et al. 2011; Briani et al. 2012; Bischoff et al. 2018) and possibly represent very primitive outer Solar System materials (Kebukawa et al. 2019).

Hydrated clasts are absent from many achondrites, including brachinites, acapulcoites, lodranites, winonaites, and angrites. Polymict ureilites are known to contain numerous clasts (e.g., Prinz et al. 1987; Brearley and Prinz 1992; Ikeda et al. 2000; Ikeda and Prinz 2001; Goodrich and Keil 2002; Ikeda et al. 2003; Goodrich et al. 2004; van Kooten et al. 2017b; Patzek et al. 2018) including genomict ones related to ureilite petrogenesis, but also xenolithic clasts such as chondrules (or fragments thereof), C2 clasts (van Kooten et al. 2017b) and C1 clasts with a high abundance of phyllosilicates (Zolensky and Ivanov 2003; Zolensky et al. 1996b; van Kooten et al. 2017b; Patzek et al. 2018; Visser et al. 2018; Goodrich et al. 2019; Bischoff et al. 2021). Based on their H- and Cr-isotope systematics, the ureilite C1 clasts may represent some of the most primitive Solar System materials (van Kooten et al. 2017b; Patzek et al. 2020). C1 material has been described within several HED meteorites (e.g., Brearley 1993; Zolensky et al. 1996a). These clasts resemble CR chondrite matrix and are suggested to represent fossil micrometeorites (Gounelle et al. 2003, 2005).

To our knowledge, phyllosilicate-bearing C1 or C2 clasts have not been reported from CO, CV and CK chondrites. However, dark clasts within these chondrite groups have frequently been described (Fruland et al. 1978; Bischoff et al. 1988; Palme et al. 1989; Kojima et al. 1993; Krot et al. 1997, 1999; Itoh and Tomeoka 2003) and could represent C1 or C2 clasts that experienced metasomatism. During this process they may have lost water, and phyllosilicates were transformed into Fe-rich olivine (Krot et al. 1997, 1999). CM chondrites are well known to be extensively brecciated (e.g., Metzler et al. 1992; Lindgren et al. 2013; Lentfort et al. 2021; Kerraouch et al. 2021, 2022), and it is unclear if some of the clasts represent C2-type xenoliths. Metal-rich carbonaceous chondrites (i.e., CR, CB, and CH) contain the most abundant hydrated clasts of all carbonaceous and non-carbonaceous chondrite groups (up to 25 vol. % in CR2 chondrite Al Rais; Weisberg et al. 1993; Bischoff et al. 1993; Endress et al. 1994), but also contain rarer C2 clasts (Bonal et al. 2010; van Kooten et al. 2017a). The highly anomalous ^15^N-enrichments in Isheyevo (CH/CB) C2 clasts have been interpreted as reflecting an accretion location in the outermost protoplanetary disk (Briani et al. 2009; Bonal et al. 2010; van Kooten et al. 2016, 2017a). Likewise, C1 clasts with high ^15^N-enrichments have been discovered in samples returned from the Cb-asteroid Ryugu (Nguyen et al. 2023). Recently, dark clasts from the CR chondrite NWA 14250 have been attributed an outer disk origin, representing a nucleosynthetic isotope endmember for the dust composition of the protoplanetary disk (van Kooten et al. 2024).

### Petrology and Mineralogy of Dark, Phyllosilicate-Bearing Clasts

#### Anhydrous Components in C2 and C1 Clasts

*Ordinary chondrites:* Among the 24 hydrous clasts found in a study of H-group regolith breccias (Fig. 4g), Briani et al. (2012) reported on the occurrence of three larger C2 clasts. The smaller C1 microxenoliths (<1 mm) include olivine and pyroxene grains, but chondrules were not observed. The larger C2-like fragments (>1 mm) contain serpentinized relict chondrules (Briani et al. 2012). These chondrules are typically <500 μm in size, but due to scarcity and heavily altered nature the size range is likely biased. Compositionally, the matrix of these C2 clasts is CM-like.

*Ureilites:* Rare porphyritic, Mg-rich chondrules (<200 μm) have been identified in C1-like clasts in ureilites (van Kooten et al. 2017b; Patzek et al. 2018). Some of these chondrules are rounded and well-preserved (Patzek et al. 2018), but the bulk consists of heavily altered, irregularly shaped chondrules. Ureilites contain C2 clasts with chondrules and CAIs (>200 μm) in a heavily hydrated sulfide-rich matrix. In contrast to C1-like clasts, these chondritic materials have Mg- and Cr-isotope signatures related to NC chondrites and that are most similar to Rumuruti chondrites (van Kooten et al. 2017b) (Fig. 4h).

*HED:* C2 clasts in HED meteorites are sub-mm to mm in size and contain chondrules and olivine/pyroxene grains and fragments thereof (Wilkening 1973; Chou et al. 1976; Mittlefehldt 1994, 2015; Zolensky et al. 1996a; Gounelle et al. 2003). In general, there is a variability in the mineralogy within the clasts reaching sizes of up to a few mm with varying abundances of chondrules (and fragments thereof), olivine and pyroxene grains, as well as TCIs, sulfides, and carbonates (e.g., Buchanan et al. 1993; Gounelle et al. 2003, 2005; Patzek et al. 2018).

*Metal-rich carbonaceous chondrites:* For metal-rich carbonaceous chondrites, C2 clasts have only been observed in Isheyevo (Fig. 4e) and the CR chondrite Northwest Africa (NWA) 14250 (van Kooten et al. 2024). Few microchondrules are present in clasts from the CR chondrite El Djouf 001 (Weisberg and Prinz 1991; Endress et al. 1994; Patzek et al. 2018, 2020) and CH chondrite Allan Hills (ALH) 85085 (Greshake et al. 2002) but are not comparable to the 100-200 μm sized chondrules in Isheyevo and 100-500 μm sized chondrules in NWA 14250 (van Kooten et al. 2017a, 2024; Gattacceca et al. 2022). Hence, we consider dark clasts with rare microchondrules as C1-type objects, or possibly, as an intermediate petrology between C1 and C2 clasts. The C2 clasts in the Isheyevo meteorite contain both microchondrules (<50 μm) and larger chondrules (100–200 μm) (Fig. 4e). For the latter, both porphyritic olivine (PO) chondrules and non-porphyritic chondrules with a glassy mesostasis have been observed, which are typically perfectly round but can also occur as fragments. The glassy chondrules are usually surrounded by metal or sulfide rims. The matrix of C2 clasts contains abundant olivine and pyroxene grains.

#### Matrix Composition and Mineralogy of C1 and C2 Clasts

The mineralogy of C1 clasts generally consists of a phyllosilicate-rich matrix, and includes magnetite framboids as well as isolated grains of magnetite, sulfides, and occasionally accessory phases such as carbonates, phosphate, and chromite [FeCr_2_O_4_]. Chondrules and fragments of anhydrous silicates (mostly forsterite-rich olivine [Mg_2_SiO_4_]) have only rarely been identified (Patzek et al. 2018). The matrix of C2 clasts is more variable and is typically linked to either CM-like compositions (including tochilinite) and CR-like compositions (including magnetite). We highlight below some variations between different clasts, which illuminate the impact of aqueous alteration and/or precursor materials.

*Ordinary chondrites:* Submillimeter-size C1 clasts in ordinary chondrites are divided into (1) hydrous magnetite-rich, (2) semi-hydrous magnetite-rich, and (3) semi-hydrous magnetite-poor (Briani et al. 2012). While all are dominated by a fine-grained phyllosilicate matrix with pyrrhotite laths, spheroidal, hexagonal and framboidal magnetite and rare dolomite and breunnerite, the second and third groups have abundant olivine and pyroxene. In the third group, Fe-Ni metal is replaced by magnetite, anhydrous silicates are less abundant and pyrrhotite dominates over troilite.

*Ureilites:* C1 clasts are dominated by fine-grained phyllosilicate-rich matrix and varying abundances of magnetite, sulfide, carbonate, and isolated grains of olivine and pyroxene (rarely chondrules) indicating intense aqueous alteration under slightly different alteration conditions (Patzek et al. 2018).

*HED:* Zolensky et al. (1996a) state that these clasts possess important similarities to matrices of CR2 chondrites with a phyllosilicate-rich matrix in which olivine grains and aggregates are embedded along with framboidal magnetite and carbonates. In general, the C1 clasts in HEDs are mineralogically similar to C1 clasts in ureilites including the variable occurrence of individual minerals such as olivine, pyroxene, magnetite, lath-shaped sulfides, and carbonates (Patzek et al. 2018).

*Metal-rich carbonaceous chondrites* (*CR, CH, CB*): Electron microprobe imaging of hydrated C1 clasts in metal-rich carbonaceous chondrites shows that these clasts are dominated by phyllosilicates (typically serpentine) and contain abundant accessory phases such as magnetite (framboidal and micron-sized individual grains), sulfides (mainly laths of pyrrhotite and pentlandite), carbonates (typically dolomite) and to lesser extent phosphates and chromite (Endress et al. 1994; Greshake et al. 2002; Bonal et al. 2010; van Kooten et al. 2017a; Patzek et al. 2018). Detailed analyses of the phyllosilicate matrix mineralogy by TEM show areas of serpentine, interwoven with organic matter, as well as regions covered by sulfides and larger organics (van Kooten et al. 2017a). TEM sections of C1-like dark clasts in NWA 14250 show very limited aqueous alteration and consist predominantly of organic-sulfide-rich regions (resembling GEMS-like materials and amorphous silicates (van Kooten et al. 2024)).

Detailed accounts of the matrix mineralogy in C2 clasts from metal-rich carbonaceous chondrites stem solely from the CH/CB chondrite Isheyevo (Bonal et al. 2010; van Kooten et al. 2017a). Secondary alteration products such as sulfides, magnetite and carbonates are absent in C2 clasts, but coarse phyllosilicates, although rare, are present with compositions indistinguishable from C1 clasts. C2 clasts contain abundant anhydrous silicates and Fe-Ni metal beads that are embedded in the matrix (<100 μm). Most of these silicates are Mg-rich low-Ca pyroxenes (En_97–99_), whereas olivines are less abundant and compositionally more variable (Fo_83–98_) (van Kooten et al. 2017a). TEM analyses of the matrix show very fine-grained phyllosilicates that are embedded together with organic matter (diffuse and globular) and magnetite grains between anhydrous silicates, some of which show alteration at the edges towards phyllosilicates.

#### Changes in Organic Matter with Aqueous Alteration

Due to size limitations, detailed studies of the organic inventory of hydrated clasts are so far limited to those observed within the Isheyevo chondrite (van Kooten et al. 2017a) and to the H chondrite Zag clast (Kebukawa et al. 2019). TEM imaging of Isheyevo C2-like clasts shows the presence of small organic nanoglobules (∼100 nm diameter) with increased C=O group functionality relative to globules in CR, CM, and CI chondrites (Le Guillou et al. 2014) (Fig. 4f). De Gregorio et al. (2013) show correlations between nanoglobule size, functional group chemistry and degree of meteorite hydration that identify the importance of increasing aqueous alteration on the growth of nanoglobules in CR and CM chondrites. Hence, the small nanoglobules in C2 clasts from Isheyevo have been interpreted to be primitive organic components formed in the cold regions of the interstellar medium. The more hydrated C1 clasts in Isheyevo are depleted in C=O bonding and enriched in N-H functional groups, whereas the C2 clasts contain relatively more C-N groups. It is uncertain, from the structural information alone, if the decrease in C=O and C-N bonding environments in C1 relative to C2 clasts is the result of increasing aqueous alteration, but correlated N- and H-isotope data of these clasts suggest that they accreted isotopically different organic precursors and ices (i.e., NH_3_ and HCN; van Kooten et al. 2017a). The pristine dark clasts in NWA 14250 invoke the presence of ISM-derived D- and ^15^N-rich organic matter that is coated with D-poor and extremely ^15^N-rich ices. As such, the ^15^N-enrichments observed in metal-rich carbonaceous chondrite dark clasts are likely outer disk signatures from vertical mixing of small icy grains (van Kooten et al. 2024).

### Hydration of Clasts

#### What Is the Protolith of These Clasts?

Variations in the overall mineralogy of hydrated clasts involve different abundances of accessory phases and the presence of anhydrous minerals such as olivine, pyroxene, and metal grains. If we compare this general description of C1 clasts to CI chondrites, we observe many similarities to CI chondrites and samples returned from asteroid Ryugu (e.g., Yokoyama et al. 2023). The CI chondrites are chemically heterogeneous objects at small scales (< 200 mg; Barrat et al. 2012; see also Morlok et al. 2006), which suggests variations in mineralogy. This small size could potentially be a reason for the observed variations in mineralogy of the hydrated clasts. *In situ* S-isotope studies of sulfides in C1 clasts and CI chondrites indicate a difference in the isotopic reservoir and therefore it appears unlikely that CI chondrites and C1-like clasts from various groups share a common origin (Visser et al. 2019). This view is further strengthened by the bulk isotopic composition of hydrated clasts in ureilites (van Kooten et al. 2017b; Patzek et al. 2019) and Isheyevo (van Kooten et al. 2017a). Thus, the protoliths for different groups of C1 clasts possess a similar initial mineralogy, although the accreted material formed in unique isotopic environments.

In some C1 clasts, we find evidence for pseudomorphs of chondrules that have been heavily altered. From bulk CI chondrites, it is clear from the absence of chondrule pseudomorphs that a relationship to C2 chondrites by progressive aqueous alteration is ruled out. Very few refractory inclusions have been reported from CI chondrites (Frank et al. 2023). Moreover, nucleosynthetic isotope signatures of bulk chondrites have established different accretion regions for their respective parent bodies. Even for the unique C1 chondrite Flensburg, which has many overlapping characteristics with CM and CI chondrites, a genetic relationship to CI chondrites is unlikely (Bischoff et al. 2021). Unless we can observe a progressive mineralogical sequence between C1 and C2 clasts, as well as identical nucleosynthetic isotope fingerprinting, we cannot confirm the link between these clasts. For example, Isheyevo C1 and C2 clasts share the same nucleosynthetic Cr- and Mg-isotope systematics, but their organic structure and isotope composition excludes a shared origin on the same parent body (van Kooten et al. 2017a). In polymict ureilites, C1 and C2 clasts have very different Cr- and Mg-isotope compositions, as well as a different matrix composition and mineralogy (van Kooten et al. 2017b).

#### Timing and Location of Clast Hydration

It is important to constrain whether hydrated clasts were altered on a larger body with fluid circulation or if the alteration occurred in a closed system similar to IDPs, including possible interaction with a gaseous environment in the disk or by cosmic ray interaction. It is also possible for these clasts to have avoided melting of accreted ices (i.e., in the absence of live ^26^Al), but having experienced alteration within the host parent body. Furthermore, clasts could have also undergone multiple alteration events on their own precursor parent bodies, and subsequently in the host parent body rendering it important to constrain their alteration histories.

Hydrated clasts from the CH/CB chondrite Isheyevo record an aqueous alteration age (i.e., 4 Myr after CAI formation) that is about 1 Myr older than the upper limit accretion age of CB chondrite Gujba (i.e., with chondrule ages of 5 Myr after CAI formation). Hence, these clasts were altered before incorporation into the host parent body, and presumably, given the continuous nature of H-isotope systematics, on the same parent body (van Kooten et al. 2017a).

For hydrated clasts in other meteorites, the timing of incorporation can, so far, only be determined from textural observations. For example, hydrated clasts in type >5 ordinary chondrites were clearly incorporated after thermal metamorphism (>50 Myr after CAI formation; Blackburn et al. 2017), but experienced hydration before this.

Some clasts appear to be overprinted by hydrothermal alteration of the host parent body: dark clasts within CO3 and CV3 chondrites have frequently been described (e.g., Fruland et al. 1978; Bischoff et al. 1988; Palme et al. 1989; Kojima et al. 1993; Krot et al. 1997, 1999; Itoh and Tomeoka 2003) and could represent C1- or C2-like clasts that experienced metasomatism during which phyllosilicates were transformed into Fe-rich olivine (e.g., Krot et al. 1997, 1999). For such clasts, it may be difficult, if not impossible, to constrain earlier alteration events.

In general, it seems likely that there were many similar small, water-rich parent bodies. Many were probably destroyed by impact and fragments (clasts) were included in the regolith of other parent bodies (ureilites, HEDs, OCs) or re-accreted with other components to form new bodies (e.g., CR, CH, asteroid 2008TC3).

There are a number of open questions that need to be addressed in future studies, and are described in the Supplementary Text.

## Low-Temperature Alteration Effects on Presolar Grains

Primitive meteorite matrices, micrometeorites, and IDPs, as well as cometary and asteroid dust returned by NASA and JAXA missions, contain low concentrations of refractory dust grains which condensed in the ejecta of evolved stars or stellar explosions (novae and supernovae) before the formation of the Sun and the Solar System. This “presolar” dust is characterized by highly anomalous isotopic compositions representing nucleosynthetic fingerprints of their stellar parents. After injection into the interstellar medium (ISM), these grains were exposed to highly energetic irradiation, shockwaves from nearby supernova explosions, and grain-grain-collisions. Presolar grains found today in primitive Solar System materials largely escaped alteration and homogenization processes in the early Solar System (e.g., Zinner 2014; Nittler and Ciesla 2016). In contrast to the more refractory presolar dust species (silicon carbide [SiC], graphite, silicon nitride [Si_3_N_4_], oxides), silicates cannot be separated chemically from their meteoritic hosts (Nguyen and Zinner 2004). Instead, they have to be analyzed *in situ* or among physically separated matrix grains with high spatial resolution techniques like NanoSIMS ion imaging (e.g., Hoppe et al. 2013), or SIMS+SCAPS (stacked CMOS-type active pixel sensor) detector systems (e.g., Nagashima et al. 2004). It is estimated that the dust fraction of the presolar molecular cloud from which our Solar System formed consisted of ∼3% circumstellar grains; while 97% of the population formed in the ISM. In the following, the term “presolar” refers to *circumstellar* presolar grains (Zhukovska et al. 2016; Hoppe et al. 2017; Alexander et al. 2017a). Silicates are the most abundant presolar dust species (≤250 ppm in meteorite matrix, up to the percent level in individual IDPs) and are large enough for the isotopic analysis of individual grains (e.g., Floss and Haenecour 2016; Nittler et al. 2018, 2021), followed by SiC (several tens ppm), refractory oxides (a few ppm to a few ten ppm), graphite (a few ppm), and Si_3_N_4_ (at the ppb-level) (e.g., Davidson et al. 2014a; Zinner 2014).

### Presolar Grain Abundances and Petrologic Type of the Host Material

The various presolar mineral phases are affected differently by low-temperature parent body aqueous alteration or thermal metamorphism. Therefore, comparing the abundances of the different presolar grain species should yield information on the degree of alteration of the grains and their host material, although several conditions have to be met: (i) the presolar grain inventories of all fine-grained primitive Solar System materials were largely the same prior to any alteration processes; (ii) the sample size (i.e., the scanned area per meteorite) has to be sufficiently large to monitor potential heterogeneous distributions of presolar dust within the matrix of a given meteorite ($>10,000~\upmu \text{m}^{2}$ per meteorite).

**Fig. 5:**
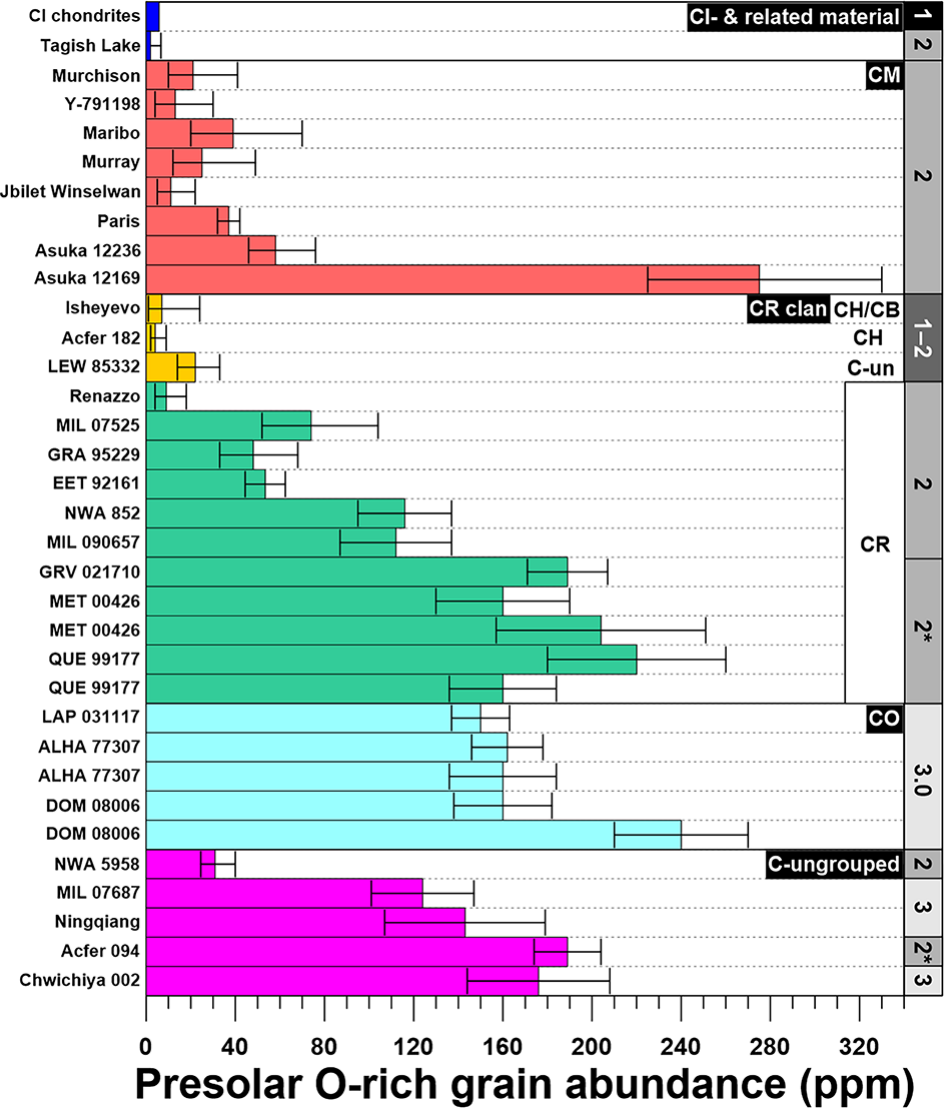
Matrix-normalized presolar O-rich grain abundances together with the petrologic types of the host meteorites. All errors are 1 sigma. Data sources: Barosch et al. (2022); Marhas et al. (2006); Leitner et al. (2012, 2016, 2018, 2020); Verdier-Paoletti et al. (2020); Nittler et al. (2018, 2020, 2021); Davidson et al. (2014b, 2015); Zhao et al. (2011, 2013); Floss and Stadermann (2009); Nguyen et al. (2010); Haenecour et al. (2018); Vollmer et al. (2009); Smith et al. (2023). *These meteorites are officially classified as type 2, but suggestions that they be considered as type 3/3.0 are supported by their high presolar silicate abundances

Silicates are more susceptible to aqueous alteration than more refractory presolar grain species like Al-rich oxides and SiC. Thus, with progressive alteration, presolar silicate abundances will decrease (e.g., Floss and Haenecour 2016) (Fig. 5). Although we observe a general trend in the presolar silicate abundances that supports this concept, this is not always the case (Fig. 5). This is evident for the CRs and CM chondrites. Among the CRs, the degree of aqueous alteration varies widely (Sect. 2.1). There is an ongoing discussion whether the CR chondrites with high presolar grain abundances (MET 00426, QUE 99177, GRV 021710) are petrologic type 2 or 3. Matrix in MET 00426 and QUE 99177 shares many similarities with the fine-grained material in ALH 77307 (CO3.00) and Acfer 094 (C-ung3.00) (Abreu and Brearley 2010), and the O-rich presolar grain abundances are also comparable (Fig. 5). However, there is evidence for aqueous alteration (Le Guillou and Brearley 2014), which would argue against a classification as type 3. Nevertheless, from a presolar grain perspective, the silicate stardust abundances in MET 00426, QUE 99177, and GRV 021710 are clearly higher than those in “moderately” altered CR2 chondrites, and also in the CM chondrites, with the exception of Asuka (A-) 12169, which is also considered to be “very primitive” (Fig. 5).

Thus, a more sensitive scale for the degree of aqueous alteration experienced by CR chondrites is desirable and could ultimately allow differences to be discerned in the presolar grain abundances due to contrasting degrees of alteration and inherent heterogeneities in the distribution of presolar dust. Several studies have attempted to establish more refined classification schemes (Alexander et al. 2013; Harju et al. 2014; Howard et al. 2015). These approaches do not provide petrological classifications that reflect the vast differences in presolar grain abundances and distributions in CR chondrites. For more extensively altered CRs, the scale devised by Harju et al. (2014) is consistent with the significantly lower presolar silicate and oxide abundances observed for Renazzo (CR2.4) (Fig. 5). In contrast, e.g., Alexander et al. (2013) assigned a petrologic type to Renazzo which puts it in line with the most pristine CR chondrites (CR2.5), indicating less aqueous alteration than in QUE 99177 (CR2.4). Several possible explanations for this apparent discrepancy exist: (i) the CRs might have been heterogeneously altered, and in addition, variations in alteration temperatures could have affected the fraction of surviving presolar silicates and oxides (Alexander et al. 2013); (ii) TEM investigations showed that amorphous silicates in the minimally altered CR chondrite MET 00426 contain water (Le Guillou and Brearley 2014), which has been ascribed to the simultaneous accretion of dust and ice. The ice could have produced localized centers of aqueous alteration on sublimation, which would explain the survival of larger amounts of presolar grains in MET 00426 and the other minimally altered CRs than in the more extensively altered CR2 chondrites, which might have experienced higher alteration temperatures. This model could also be applicable to the CM chondrites. Here, the presolar silicate and oxide abundances for petrologic types ≤2.6 are generally low and suffer from comparably large statistical errors (due to small grain numbers), while A-12169, the less altered regions of Paris (estimated ≥2.8), and A-12236, show higher abundances (Verdier-Paoletti et al. 2020; Nittler et al. 2021). The bulk O-isotope compositions of fine-grained matrix of CR chondrites (Schrader et al. 2014) appear to correlate quite well with both the degree of alteration of the respective meteorite and the abundances of presolar O-anomalous grains. So far, there is only little overlap between the sample sets, and differing results for individual samples from the same meteorite seem to indicate heterogeneous alteration, and more work is needed.

A combination of low- and high-temperature alteration could potentially “reset” petrologic type and be responsible for comparably low presolar silicate abundances in several chondrites that appear to be pristine at a first glance. LEW 85332, an ungrouped member of the CR clan, has been classified as C3.0–3.1 (Rubin and Kallemeyn 1990). However, its matrix was aqueously altered, and subsequently heated to ∼500–600 °C (Brearley 1997; Tonui et al. 2002), and despite its apparent low petrologic type, it has a low presolar silicate abundance (∼22 ppm; Leitner et al. 2018).

The abundances of presolar oxides are typically low (a few ppm) and the small grain numbers are responsible for large errors for the respective ratios. Thus, statistically significant silicate/oxide ratios are only available for very thoroughly studied meteorites, or specimens that contain unusually high abundances of presolar oxides (e.g., Leitner et al. 2012, 2020 and references therein). Haenecour et al. (2016) investigated the silicate/oxide ratio of sub-micrometer-sized matrix silicates and oxides in LAP 031117 (CO3.0). The elemental compositions and compositional range of both presolar and non-presolar silicates showed no significant differences. This indicates that the sub-micrometer-sized non-presolar silicate and oxide populations could also be used to assess the degree of alteration of a given matrix area, but with better statistics.

A more promising approach might be to use the presolar SiC abundances instead, which are typically in the range of a few tens of ppm (e.g., Davidson et al. 2014a) (Fig. S1). However, this requires even more effort, since both O-anomalous and C-anomalous grain abundances have to be measured. Such investigations are relatively time-consuming, especially if presolar grain abundances are low. Moreover, for a few meteorites, as well as for a matrix clast in material from asteroid Ryugu, higher SiC abundances have been observed; this fact might complicate the use of presolar silicate/SiC ratios as indicators of matrix alteration.

### Remaining Questions and Future Work

More comprehensive studies of the presolar silicate and oxide inventories of moderate to heavily altered chondritic matrix would yield better statistics and allow monitoring of differences between the presolar grain populations of chondrites of petrologic types 1, 2 and 3. However, such studies will be more time-consuming than those of more pristine meteorites, owing to the lower stardust abundances in the target material, especially when the abundances of carbonaceous presolar phases are investigated in addition.

Even if presolar silicates are not destroyed by aqueous alteration, their chemical compositions can be affected (Fig. S2). Iron is of particular interest in this context, and more detailed studies of Fe in presolar silicates could allow discrimination between “primary” and “secondary” Fe, the latter introduced into the grains by aqueous alteration processes (Supplementary text). This could be achieved by measurements of the Fe oxidation states in presolar silicates, e.g., by TEM-based EELS and high-spatial resolution measurements of Fe-isotopes. Generally, extending the dataset of elemental compositions of presolar silicates from petrologic type 1 and 2 chondrites would allow searching for systematic differences caused by parent body alteration between these grains and those from highly primitive meteorites.

## Evolution of Organic Matter During Low-Temperature Aqueous Alteration

### Introduction

Organic matter likely underwent modification during aqueous alteration. It is divided into soluble organic material (SOM) and insoluble organic matter (IOM). The SOM is extracted using water or organic solvents, while the IOM is the residue recovered after demineralization. IOM shares similarities with type III terrestrial kerogen (Quirico and Bonal 2019). Unlike kerogens, IOM and SOM exhibit enrichments in D and ^15^N (Robert and Epstein 1982; Kerridge et al. 1987; Alexander et al. 2007). Chondritic organic matter was likely synthesized at very low temperatures (<40 K) in the local interstellar medium or in the outer solar nebula (Robert and Epstein 1982; Kerridge 1983; Yang and Epstein 1983, 1984; Alexander et al. 1998, 2007; Remusat et al. 2006). IOM displays elemental, structural and isotopic variations within and between chondritic groups (e.g., Alexander et al. 2017b). However, the causes of these variations are still debated. Do they originate from different mixtures of heterogeneous precursor materials, or are they the products of aqueous alteration in the chondrite parent bodies? (Alexander et al. 2007, 2010, 2017b; Remusat et al. 2010; Orthous-Daunay et al. 2013; Le Guillou et al. 2014; Vollmer et al. 2014, 2020b; Vinogradoff et al. 2017; Changela et al. 2018).

### The Impact of Aqueous Alteration on the Organic Matter

The CR, CM, and CI chondrites, along with the ungrouped C2 Tagish Lake chondrite, exhibit varying degrees of aqueous alteration, offering valuable insights into the evolution of organic matter through aqueous alteration. Pearson et al. (2002) first noticed the spatial association between organic matter and clay minerals using SEM and osmium labelling, that was later confirmed by scanning transmission X-ray microscopy (STXM) and TEM studies (Garvie and Buseck 2007; Le Guillou et al. 2014; Vinogradoff et al. 2017). Le Guillou et al. (2014) reported diffuse organic components intimately distributed among phyllosilicates and connected to fractures, likely indicating fluid transport at the micrometer scale. Changela et al. (2018) compared CR2 with CR1 chondrites (Al Rais and GRO 95577) and interpreted the observed molecular changes as cracking occurring in the presence of fluid at temperature above 150 °C.

Other indications of the influence of aqueous alteration on organic matter are evident in the diverse lithologies of the Tagish Lake chondrite, which have experienced varying alteration conditions (Zega et al. 2010; Blinova et al. 2014b). Alexander et al. (2014) found a correlation between the degree of alteration of the host lithology and decreases in both the $\delta $D values and bulk H/C, accompanied by increases in aromaticity of their IOM. These authors suggested that the IOM in Tagish Lake lost its D-rich material and/or underwent isotopic exchange with water possibly as a result of short-term heating. Vinogradoff et al. (2017) also observed similar trends between the IOM extracted from the weakly altered CM Paris and the more altered CM Murchison. Laser desorption ionization coupled to ultra-high-resolution mass spectrometry on various IOM from CM chondrites reveals the gradual loss of heteroatoms and increase in aromaticity with increasing alteration degree (Laurent et al. 2022a). It also shows that the IOM of CMs and CIs preserve records of slightly different precursors.

The diverse chondritic groups might have accreted IOM of different compositions, suggesting that aqueous alteration only played a minor role. Remusat et al. (2010) reported D-rich IOM hotspots in the matrices of CR, CM, and CI chondrites, suggesting minimal or no isotopic diffusion has occurred between the organic matter and hydrous minerals. Conversely, Alexander et al. (2017b) argued that water-organic exchange is slower than diffusion into the grains. Other evidence from the alkyl abundance (CH_2_ and CH_3_) in aqueously altered chondritic IOM shows no correlation with alteration degree (Orthous-Daunay et al. 2013), but instead reflects thermal processing or precursor heterogeneity (Quirico et al. 2014).

### Soluble Organic Matter

The SOM in carbonaceous chondrites is composed of huge diversity of compounds, with tens of thousands of molecules containing C, H, N, O and S (Schmitt-Kopplin et al. 2010), a diversity also found in Ryugu samples (Schmitt-Kopplin et al. 2023). There have been many studies focusing on amino acids, nucleobases, carboxylic acids and amines (see Glavin et al. 2018 for a review), while recent papers have reported the distribution of polycyclic aromatic hydrocarbons (PAHs) in CM chondrites (Sephton et al. 2000; Kalpana et al. 2021; Graham et al. 2022; Lecasble et al. 2022). If aqueous alteration seems to have a subtle impact on the molecular distribution of these chemically recalcitrant molecules, their H-isotope composition has likely been disturbed by exchange with the fluid during aqueous alteration (Lecasble et al. 2023). Their C-isotope signatures, however, are inherited from pre-accretion synthesis (Naraoka et al. 2000).

Amino acids, which have been studied extensively due to their biological interest, are found in over 90 different forms, including isomers (Glavin et al. 2018). Their abundances significantly vary within and between chondrite groups, with the highest contents reported in some CR chondrites (up to 300 ppm vs. 80 ppm in the Murchison meteorite). In the CM and CR groups, the amino acid content decreases with increasing aqueous alteration (Martins et al. 2007; Glavin et al. 2020), suggesting that aqueous alteration might cause amino acid decomposition or introduce analytical bias, as more altered chondrites contain more phyllosilicates that could trap amino acids. Amino acid yields increase when the water extract is subjected to acid hydrolysis, likely indicating the presence of small peptides or amino acids precursors in carbonaceous chondrites (Serra et al. 2022). The amino acids form through various synthetic routes, including the Strecker synthesis, Michael addition or formose reaction (Elsila et al. 2012; Vinogradoff et al. 2020b). Some amino acids can exhibit enantiomeric excesses (Engel and Nagy 1982), with excesses of the L-form, notably of the non-proteaginous isovaline, reported in various carbonaceous chondrites (Glavin and Dworkin 2009). These excesses tend to be larger in more altered meteorites, suggesting enantiomeric excess amplification during parent body alteration (Glavin and Dworkin 2009), though irradiation by circular polarized light could also explain the enantiomeric excess (Modica et al. 2014).

### Laboratory Experiments

Experimental studies can help to better understand the behaviour of organics during hydrothermal alteration. Two approaches have been followed: (i) study of the evolution of molecules akin to those detected in chondrites, including SOM and IOM, (ii) assumptions about the nature of accreted molecules.

Hydrous pyrolysis experiments on IOM from Murray and Murchison at ∼300 °C revealed a decrease of the H/C, $\delta$D and $\delta$^15^N values, and a lower aliphatic content (Yabuta et al. 2007; Oba and Naraoka 2009). The release of PAHs and carboxylic acids in the aqueous solutions showed that IOM can be a source of SOM. Laurent et al. (2022b) subjected the IOM of the CM Paris to aqueous alteration at 150 °C for ∼50 days. They observed that the fraction of aromatic C increases with alteration, while D-rich hotspots are almost unaffected. The temperature and duration of the experiments affect the molecular and isotope evolution of the IOM, with rapid modifications of the isotopic, elemental and structural properties occurring at ≥250 °C (Foustoukos et al. 2021). Kebukawa et al. (2021) suggested that D/H isotopic exchange between D-rich IOM and D-poor water must occur slowly at low temperatures (probably close to 0 °C) to prevent equilibration of the D/H ratio between organic matter and water on timescales of millions of years.

Formaldehyde (H_2_CO) or hexamethylenetetramine (HMT, (CH_2_)_6_N_4_) could have been accreted directly, as they are found in the interstellar medium, comets or ice irradiation experiments. Upon heating in the presence of water, both go through the formose reaction and polymerize. This reaction has also been proposed to account for the formation of sugars (Furukawa et al. 2019). Cody et al. (2011) showed that formaldehyde in aqueous solution at 250 °C could form IOM and globule-like morphologies similar to chondritic IOM. Follow up studies investigated the effect of NH_3_ in the original mixtures, as well as the kinetic evolution of the products (Kebukawa et al. 2013; Kebukawa and Cody 2015). HMT follows a similar behaviour (Vinogradoff et al. 2017). The presence of phyllosilicates modifies the chemical pathways, lowering IOM yield and trapping organic matter within their interlayer (Vinogradoff et al. 2020a; Viennet et al. 2022). Amino acids are also produced in all these experiments (Kebukawa et al. 2017; Vinogradoff et al. 2020b).

### Remaining Questions and Future Work

Critical questions relating to the effects of low-temperature alteration on organic matter are described in the Supplementary text.

## Physicochemical Modelling and Hydrothermal Alteration Experiments

Important questions remain regarding the physicochemical conditions (e.g., pH, $f$O_2_, fluid composition, etc.), mechanisms, and rates of alteration in chondrite parent bodies. Two main approaches have been used to address these questions: (1) thermodynamic and kinetic modelling of alteration processes in systems of different compositions and/or (2) alteration experiments carried out under controlled settings. Both approaches have some limitations. In thermodynamic modelling, the most serious limitations are: (*i*) the lack of appropriate thermodynamic data for the minerals/phases of interest and (*ii*) the lack of complete chemical equilibrium in the system even though some coexisting phases may have been in local equilibrium. The major limitation of laboratory experiments are: (*i*) slow rates of alteration reactions and (*ii*) a large number of physicochemical parameters controlling such reactions. As a result, a comprehensive experimental study of these processes becomes economically prohibitive. However, a proper combination of both approaches, with thermodynamic and kinetic modelling followed by intelligently designed experiments, provides insights into relative sequences of complicated alteration reactions and physicochemical conditions under which the observed reactions most likely occurred.

### Physicochemical Modelling

Two major types of thermodynamic modelling used in simulation of alteration processes are (1) evaluation of observed alteration reactions at a range of pressure ($P$), temperature ($T$), and activities of involved phases and ions (e.g., Alexander et al. 1989; Krot et al. 1998) or (2) calculation of full chemical equilibrium in a system of assumed composition at specified $T$ and $P$ (e.g., Zolensky et al. 1989; Rosenberg et al. 2001; Schulte and Shock 2004; Zolotov et al. 2006). The latter type of modelling is briefly reviewed here; details can be found in the original publications referenced below.

Zolensky et al. (1989) were the first to use an equilibrium code (Eq. 3/6) to model interaction between liquid water containing dissolved CO_2_ with concentrations of total C ranging from $10^{-8}$ to $10^{-2}$ moles and varying amounts of an anhydrous mineral assemblage (olivine + orthopyroxene + glass + spinel + chromite + Fe-Ni metal + Fe sulfides) similar to either CM or CV chondrites at temperatures of 1, 25, 50, 99, and 150 °C. While the pressures are not explicitly reported, these should have been at least equal to the saturated pressures of H_2_O vapor at these temperatures, *i.e.* 0.007, 0.03, 0.12, 0.98, and 4.75 bars, respectively. It was found that the alteration mineralogy of the CM chondrites is best reproduced by interaction of the anhydrous precursor mineralogically similar to the CM chondrites with water containing C in a wide range of concentrations at temperatures of 1 to ∼25 °C. The alteration mineralogy is essentially the same for a wide range of W/R ratios. Under these conditions solution pH changes from 7 to >12, with most alteration taking place at a pH greater than ∼10. Solution Eh changes from −0.5 to −7.5 V, with increasing alteration. The mineralogy of the CI chondrites can be well-reproduced by alteration of either CM or CV anhydrous precursor at temperatures of 50–150 °C and high C concentrations in aqueous solution of $10^{-3} \text{--}10^{-2}$ moles for a wide range of W/R ratios. Under these conditions solution pH and Eh change from 7 to ∼10 and from −0.3 to −0.8 V, respectively, as alteration progresses

A follow up study by Rosenberg et al. (2001) focussed on more detailed modelling of CM chondrite alteration using the REACT code, which is very similar to Eq. 3/6. The calculations were performed at 25 °C using two different precursors, ‘reduced’ and ‘oxidized’, with most Fe being present as metal in the former and fayalite [Fe_2_SiO_4_] in the latter, and about the same amount of Fe sulfide in both. The mineralogy of the precursors is qualitatively similar to that of Zolensky et al. (1989), but their chemical compositions differ significantly. The results of modelling are generally consistent with those of Zolensky et al. (1989). The alteration mineralogy of the ‘reduced’ and ‘oxidized’ precursors is mostly similar. The main difference is a release of large volumes of H_2_ gas in the former that has to be lost (open system behaviour) to allow alteration reactions to proceed further and produce Fe-rich silicates. Rosenberg et al. (2001) noted that the aqueous solutions produced by such alteration are ‘dominated by Ca and Si and are strongly basic and reducing’.

Schulte and Shock (2004) used the Eq. 3/6 code to model reactions of a rock of the average CM chondrite composition with a variety of different aqueous solutions of unspecified compositions at temperatures of 2–200 °C and pressures of saturated H_2_O vapor, focussing on the possible formation of soluble organic matter during aqueous alteration. Their results are generally consistent with the studies mentioned above. They concluded that “alteration of the CM parent body occurred at temperatures perhaps as high as ∼150 °C at a W/R ratio greater than 1 and involved a fluid with concentrations of C and N greater than 0.1 molal. The fluid was initially more oxidizing than the rock and the pH of the fluid was much greater than neutral.”

Since 2006, Zolotov and colleagues (Zolotov 2012, 2014, 2017; Zolotov and Mironenko 2007, 2008a, 2008b; Zolotov et al. 2006, 2015) investigated water-rock interaction in several systems which emulate aqueous alteration in CR, CM, CI, CV and ordinary chondrites. They used the equilibrium GEOCHEQ code equipped with a module accounting for mineral dissolution kinetics in addition to phase equilibria. The open and closed chemical systems explored include major rock-forming and volatile elements (O, H, C, Cl, S, Mg, Fe, Ca, Na, K, Mn, Si, Al, Ni, Co, Cr, P) partitioned among solid, gaseous, and aqueous solutions at temperatures and pressures up to ∼300 °C and 300 bars, respectively, W/R ratios up to 1000, and a wide ranges of assumed porosities of unaltered precursors. So far it is the most comprehensive physicochemical study of aqueous alteration of primitive meteorites and their putative or sampled (asteroid Ryugu; Nakamura et al. 2023) parent bodies. In addition to mineralogical changes, which are generally consistent with the previous results described above, they report a great deal of information on chemical composition and speciation of gaseous and aqueous solutions and their relative amounts that is very useful for planning laboratory experiments on hydrothermal alteration. The major finding of these studies are (1) depending on local W/R ratio and $T$ during interaction of primary anhydrous chondritic minerals with H_2_O-rich ices, H_2_O can be present as mostly vapor or vapor + aqueous solution or mostly aqueous solution; (2) the mineralogies of alteration products are relatively independent of starting ‘chondritic’ composition and are mainly controlled by the local W/R ratios, $T$, amounts of anion-forming elements such as Cl, C, N in a water source/ice, and the dissolution kinetics of primary phases; (3) the dissolution rates of primary minerals increase in order forsterite → enstatite/diopside [Mg_2_Si_2_O_6_/CaMgSi_2_O_6_] → feldspar → Fe-Ni metal resulting, as alteration progresses, in formation of magnetite + Fe-rich serpentine first, followed by Mg,Fe-serpentine + magnetite + Ca-Na-K-Fe-bearing saponite ± pyrrhotite, and finally Fe-rich phyllosilicates + fayalite when solutions is consumed; (4) the pH of the aqueous solution changes from neutral or slightly acidic (if Cl is present in the ices) at the beginning to progressively more alkaline; (5) the dominant solutes are $\text{Na}^{+}$, $\text{Cl}^{-}$, $\text{HCO}_{3}^{-}$ with subordinate amounts of $\text{Ca}^{2+}$, $\text{Mg}^{2+}$, $\text{Fe}^{2+}$, etc.; (6) oxidation of sulfides is kinetically inhibited, so formation of sulfates requires photochemical and radiolytic processes that produce O-rich oxidants either derived from or accreted with ices.

### Hydrothermal Alteration Experiments

These experiments aim to replicate alteration conditions inferred from meteorites, tracking mineralogical and chemical evolution at every stage. Comparing experimental products with meteorites can test models of alteration and constrain key factors such as the initial mineralogy, timescales, fluid compositions, and W/R mass ratios.

The original mineralogies and initial stages of alteration are difficult to constrain in highly altered CM and CI chondrites where little of the precursor material remains. Nonetheless, mineralogical observations of the least altered type 3 carbonaceous chondrites suggest that Fe-Ni metal grains, anhydrous silicates and/or amorphous silicates were precursors to secondary minerals (Abreu and Brearley 2010; Davidson et al. 2019; Dobrică and Brearley 2020). That the matrices of the least altered chondrites often contain amorphous silicate grains that are susceptible to aqueous alteration (Leroux et al. 2015) has led several studies to use amorphous materials as reactants in alteration experiments (e.g., Nakamura-Messenger et al. 2011). Le Guillou et al. (2015b) produced micrometer-thick layers of synthetic amorphous or crystalline silicates of fayalitic composition and exposed them under anoxic conditions to deionized water at a W/R mass ratio of 150, and $T $ of 90 °C for two weeks or 190 °C for two hours. At both temperatures, the surfaces of the amorphous films were altered into an amorphous assemblage with a Fe-rich serpentine and a Mg-rich phyllosilicate composition (similar in composition to saponite), replicating some important aspects of the amorphous Fe-rich material and oxidized phyllosilicates reported in CR chondrites.

To understand the formation mechanisms of TCIs in CM chondrites (see Sect. 2.2), Vacher et al. (2019b) conducted hydrothermal experiments on synthetic glass, iron metal (Fe^0^) particles and forsterite at 80 °C for 30–60 days, at a W/R mass ratio of 10 under anoxic conditions. The experiments produced crystals of tochilinite and cronstedtite from the reaction between synthetic glass, Fe^0^ and S-bearing or S-free water, respectively, and demonstrate that TCIs are likely formed from the dissolution of amorphous silicates and Fe-Ni metal in CM chondrite matrices.

Forsterite and enstatite are the main anhydrous silicates in carbonaceous chondrites, often occurring as chondrule phenocrysts. Ohnishi and Tomeoka (2007) explored hydration of enstatite, altering synthesized Mg-enstatite grains at temperatures of 100–300 °C and pH of 0–14 for between 1–14 days. Alteration of enstatite was only observed under neutral to alkaline conditions, with the abundance of phyllosilicates (serpentine and saponite, similar to those found in CI chondrites) increasing with temperature and/or run duration. This suggests that a variety of Mg-Fe-rich minerals, in particular enstatite and Mg-Fe olivine, act as precursors during extensive aqueous alteration to promote the formation of Mg-bearing serpentine and/or saponite.

#### The Timescales of Aqueous Alteration

The Mn-Cr and I-Xe chronometers indicate that alteration in carbonaceous chondrites lasted for extended periods of time (∼2–15 My after CV CAIs; Fujiya et al. 2012; Jilly-Rehak et al. 2017; Pravdivtseva et al. 2018) (Sect. 9). However, experiments applicable to carbonaceous chondrites suggest that alteration may have occurred on short timescales, even at low temperatures. For example, Le Guillou et al. (2015b) reported that the reaction rate of a crystalline film is ten times slower than an amorphous film, suggesting that under low temperature conditions (90 °C) chondritic amorphous silicates are probably altered rapidly relative to the timescales on which asteroid interiors were heated by radiogenic decay (i.e., within a few million years). Jones and Brearley (2006) hydrated cubes of a CV chondrite under highly oxidising conditions at W/R mass ratios of 1:1 to 9:1 and temperatures of 100–200 °C for 7–180 days. Altered cubes had Ca- and Mg-sulphates, Ca-carbonates, and Fe-(oxy)hydroxides on exterior surfaces, while interiors contained serpentine interlayered with saponite and an amorphous SiO_2_-rich phase. This assemblage resembles the CI chondrites (except the sulphates, which form due to terrestrial weathering, e.g., Imae et al. 2024), and demonstrates that phyllosilicate formation within asteroids was likely rapid, on the order of days to weeks at temperatures of ∼150 °C, and 100s to 1000s of years at temperatures of <100 °C.

#### What Was the Composition of the Alteration Fluid and Was the Alteration Under a Closed or Open System?

Determining fluid compositions using hydrothermal experiments has received little attention. Vacher et al. (2019b) used S-rich (pH = 11.5) and S-free (pH = 6.5) fluids to form tochilinite and cronstedtite, respectively. The pH has an important influence on dissolution of silicate glass; in S-free experiments the Mg/Si ratio of the solutions was higher (i.e., less Si was dissolved), permitting the precipitation of Mg-rich minerals, whereas cronstedtite was only formed with S-free fluids and reactants with high Fe contents. Tochilinite was only identified in experiments using S-rich fluids. Vacher et al. (2019b) proposed a model for TCI formation whereby the precipitation of tochilinite caused the S activity of the fluid to decrease to the point at which Fe-Si-rich phyllosilicates could precipitate.

Models generally assume alteration in a closed system due to the apparent isochemical composition of altered carbonaceous chondrites and their low permeability (Bland et al. 2009). Such properties indicate limited fluid flow and chemical transport inside asteroids. Alternative models predict large-scale transport over km scales in an open system through exhalation flow from the interior to the surface of the asteroid (Young et al. 1999, 2003; Palguta et al. 2010). These models are supported by meter-sized carbonate veins at the surface of B-type asteroid Bennu (Kaplan et al. 2020). Suttle et al. (2022b) altered CO chondrite chips in Teflon reaction vessels, with gases able to escape and produce a partially open system. Chondrules in the chips remained largely unaltered, whereas the matrix displayed localized alteration features including Fe-enrichments (leached from Fe-Ni metal), decreased porosity, and the opening of large ∼100 μm wide channels. Alteration products included Fe-(oxyhydroxides), magnetite, and fayalite, plus minor Fe-sulfides, carbonates, and sulphates, with the alteration similar in stye to the Fe-alkali metasomatism reported in CV chondrites.

Kikuchi et al. (2022) altered synthetic chondrite mixtures at reducing conditions within Teflon bottles. To approximate the loss of H gas from an asteroid, the bottles were deliberately opened every 5–7 days to remove the headspace air. Saponite encrusted primary minerals in all experiments, with its formation taking 1 day at 80 °C and 200 days at 25 °C. It was proposed that phyllosilicate formed via direct transformation (i.e., replacement of the initial phase without an intermediate) or a two-step process involving an SiO_2_-rich intermediate phase following the dissolution and recrystallization of silicates. Serpentine was not detected, with the experimental products most closely resembling the mildly altered CO, CR, and CM chondrites.

#### Remaining Questions and Future Work

Despite extensive data regarding alteration processes having been accumulated through laboratory experiments, numerous questions persist. Conducting further experiments could provide new insights, including: (1) was water completely consumed during aqueous alteration, or did the alteration occur in an open system; (2) can mineral textures provide information concerning kinetics and fluid compositions; (3) how does terrestrial weathering affect the formation of secondary phases; (4) do isotopic exchanges occur during low-temperature aqueous processes; (5) what are the triggers for the formation of secondary phases, considering the complexity and variability observed due to multiple episodes of alterations; (6) how would the addition of anionic elements (C, N, S) to the experimental solutions affect the secondary mineralogy?

## Fluid Inclusions in Aqueously Formed Minerals

Fluid inclusions in a mineral retain the aqueous fluid that was present when the mineral was formed and are fossils of the aqueous fluid that mediated aqueous alteration. They provide information on the composition of the ices that caused the aqueous alteration, and alteration conditions. Two major types of fluid inclusions that are not contaminated with terrestrial water or other liquids are currently found in extraterrestrial samples: those in halite (NaCl) clasts in H chondrites (Zolensky et al. 1999c; Whitby et al. 2000; Rubin et al. 2002), and those in minerals in an aqueously altered carbonaceous chondrite (Tsuchiyama et al. 2021) and related material from the asteroid Ryugu (Nakamura et al. 2023).

### Fluid Inclusions in Halite Clasts in H Chondrites

Two H chondrites, Monahans (1998) (hereafter Monahans) (H5) and Zag (H3-6) are regolith breccias and have clasts consisting of blue to purple halite (NaCl) crystals up to 1 cm in size (mostly a few mm in Monahans and a few hundred μm in Zag). Fluid inclusions were found in these halite crystals for the first time in extraterrestrial materials (Fig. S3a) (reviewed by Zolensky et al. 2017). The Monahans halite contains inclusions of sylvite (KCl) (Rubin et al. 2002). The color is thought to be due to solar/galactic cosmic ray irradiation or exposure to ^40^K beta decay of the sylvite (Zolensky et al. 2017). They have early Solar System formation ages (4.66 ± 0.08 to 4.03 ± 0.05 Ga, Zolensky et al. 1999c; Bogard et al. 2001; Whitby et al. 2000).

Fluid inclusions in the halite grains are mostly <10 μm in size (Zolensky et al. 1999c; Rubin et al. 2002) and sometimes have bubbles inside (∼25% of Monahans; Zolensky et al. 1999c). Faceted cubic negative crystals are also observed (Fig. S3a). Raman analysis revealed a significant peak at $3400\text{ cm}^{-1}$, corresponding to an aqueous salt solution (not pure water), whereas CO_2_, N_2_, and CH_4_ were not detected (Zolensky et al. 2017). The freezing and melting temperatures of the fluid inclusions suggest the absence of CO_2_ and likely indicate the presence of ($\text{Fe}^{2+}$, $\text{Ca}^{2+}$, or $\text{Mg}^{2+}$) in addition to Na^+^ and K^+^, respectively (Zolensky et al. 1999c). Their formation temperature estimated from the bubble volume was <100 °C (possibly <50 °C). The O- and H-isotope compositions of the fluids measured using cryo-SIMS varied widely among the individual fluid inclusions, −400‰<δD<+1300‰ and $-20‰<\Delta^{17}\text{O}<+30‰$ (Yurimoto et al. 2014), indicating isotopic disequilibrium before incorporation into the halite. This variation is explained by the mixing of carbonaceous chondritic water ($\delta $D-poor, low $\Delta ^{17}\text{O}$) and outer Solar System (cometary) water ($\delta $D-rich, high $\Delta ^{17}\text{O}$) during rock-water interaction. The presence of some of these cations in the Monahans fluid inclusions, as well as a large assemblage of organic molecules, was subsequently demonstrated by ToF-SIMS (Bodnar et al. 2019).

A variety of mineral inclusions have also been found in halite: olivine, high- and low-Ca pyroxenes, feldspars, magnetite, Fe-Ni sulfides, Fe-Ni metals, lepidocrocite (FeO(OH)), carbonates, diamond, apatite [Ca_5_(PO_4_)_3_(F,OH,Cl)], phyllosilicates, and zeolites (Fries et al. 2011; Zolensky et al. 2017). Halite also contains a variety of carbonaceous materials and organic matter: macromolecular C, aliphatic materials, and amino acid precursors (Fries et al. 2011; Kebukawa et al. 2016; Chan et al. 2018). These are thought to be organic matter precipitated from brines along with halite in the parent body. Similar organic matter has been found in dark clasts in Monahans and Zag and has been linked to fluid inclusions (Kebukawa et al. 2016, 2019, 2023).

The formation temperature of fluid inclusions shows that the halites with fluid inclusions were incorporated into the H chondrites after thermal metamorphism (approximately 700 °C for H5). This is thought to be related to cryovolcanism of ice-bearing small bodies, where the eruption of brine containing organic matter and rocky mantle components occurred (Fries et al. 2013; Zolensky et al. 2017). C-type asteroid Ceres is a possible source because of the similarity of mineralogy between inclusions in the halite and the Ceres regolith observed by Dawn and of the intersecting orbits of Ceres and 6 Hebe proposed as the origin of the H chondrites.

### Potential Fluid Inclusions in Carbonaceous Chondrites

Carbonaceous chondrites that have undergone aqueous alteration contain minerals that grew from aqueous fluids. Thus, fluid inclusions in these minerals have been sought in CIs, CMs, and the Tagish Lake meteorite after careful sample preparation to avoid water contamination; potential fluid inclusions have been found in carbonates, sulfides, olivine, and enstatite (Zolensky et al. 2017). Some of these contain bubbles, but because of their small size (<a few μm), they have not been confirmed as fluid inclusions by chemical analysis.

X-ray tomography (XCT), a nondestructive analytical technique, is highly effective in the search for fluid inclusions. In particular, microsampling of a region of interest (ROI) using a focused ion beam (FIB) and imaging of the ROI with X-ray nanotomography (XnCT) make it possible to search for even small fluid inclusions. By combining two different XnCT techniques using synchrotron radiation, namely dual-energy tomography (DET) for absorption images (Tsuchiyama et al. 2013) and scanning-imaging X-ray microscopy (SIXM) for phase-shift images (Takeuchi et al. 2013) (DET-SIXM method), many mineral phases, plus organic matter and aqueous fluid, can be identified with an effective spatial resolution of ∼200 nm (Matsumoto et al. 2019). A fluid inclusion (∼7 μm) with a bubble in terrestrial quartz microsampled by FIB from a thin section was clearly imaged using DET-SIXM (Yoshida et al. 2016).

By using this method, a number of inclusions <∼5 μm were observed in calcite from the Sutter’s Mill (CM) chondrite (Tsuchiyama et al. 2014, 2021; Zolensky et al. 2014, 2017). However, no fluid was observed in micrometer-sized inclusions, although some had facets (negative crystals) which may indicate that they once contained aqueous fluid (Fig. S3b). IOM inclusions with bubbles (Fig. S3c) were confirmed using microRaman, TEM, STIM-XANES, and NanoSIMS analyses performed after XnCT (Tsuchiyama et al. 2017). IOM inclusions also occur in enstatite (Tsuchiyama et al. 2017). This is consistent with the observation that IOM inclusions are universally present in CM carbonates (calcite and dolomite) (Chan et al. 2017). Many inclusions found in Fe sulfides and Fe-Ni sulfides are irregular in shape and connected outward in three dimensions (Tsuchiyama et al. 2014).

### Fluid Nanometer-Sized Inclusion in Sutter’s Mill (CM) Calcite

Numerous nanometer-size inclusions (<1 μm) were identified by XnCT (Fig. S3b) (Tsuchiyama et al. 2021) but their contents could not be examined owing to spatial resolution limitations. Well-developed {001} facets of these inclusions observed by TEM (Fig. S3d) indicate the possibility that aqueous fluid remained in some inclusions. Aqueous fluid was identified in one of them (Fig. S3e) by selected area electron diffraction (SAED) patterns of the inclusion in cryo-TEM, showing additional reflection spots identified as CO_2_ ice or CO_2_ hydrate ($\text{CO}_{2}\cdot 5.75\text{H}_{2}\text{O}$) at −100 °C. According to the phase diagram of the CO_2_-H_2_O system (Longhi 2005), this fluid is enriched in CO_2_ (CO_2_/H_2_O>∼0.15). Some nanometer-sized particles of Na-Mn and Fe sulfates, and FeOOH (or hematite [Fe_2_O_3_]), that occur as trapped or daughter crystals in some inclusions, suggest that the fluid was brine. The estimated pressure at which the CO_2_-rich fluid was incorporated during aqueous alteration within the Sutter’s Mill parent body was P>∼50 bars (possible >100 bars), suggesting that fluid uptake likely occurred in the interiors of relatively large bodies of >100 km (possibly >200 km).

CO_2_-rich ice has been proposed as the origin of C in the CO_2_-rich fluid, in which case the Sutter’s Mill parent body may have formed outside the CO_2_ snow line and inside the CO snow line (∼75 K and ∼ 22 K, respectively; Okuzumi et al. 2016). This is consistent with the Grand Tack model (e.g., Walsh et al. 2012), which suggests that the parent body formed outside the orbit of Jupiter in the early Solar System and then moved inward, as well as with the isotope dichotomy of Solar System materials (e.g., Warren 2011).

### Fluid Inclusions in Ryugu Samples and CI Chondrite?

The Hayabusa2 spacecraft collected samples from the surface/subsurface of the Cb-type asteroid Ryugu and returned them to Earth in 2020 (Tachibana et al. 2022). They were identified as CI meteorites or related materials in the initial analysis (e.g., Nakamura et al. 2023). Potential fluid inclusions of a few μm were found in pyrrhotite grains by XnCT (Fig. S3f) and confirmed by cryo-TOF-SIMS (Nakamura et al. 2023). Secondary ion signals detected inside the inclusion indicate that it was filled with a fluid containing H_2_O, CO_2_, S species, and N- and Cl-bearing organic compounds. The presence of CO_2_ indicates that Ryugu’s parent body also formed outside the CO_2_ snowline. There are many possible IOM inclusions in pyrrhotite, dolomite, breunnerite, and apatite grains; however, no potential fluid inclusions have been identified thus far (Tsuchiyama et al. 2024). Fluid inclusions in dolomite have also been explored in Ivuna (CI) using DET-SIXM and cryo-TEM (Tsuchiyama et al. 2019). Ivuna dolomite also had numerous micrometer- and nanometer-sized inclusions, similar to calcite in Sutter’s Mill. Some micrometer-sized inclusions had facets, but they were empty. Nanometer-sized inclusions also had facets, but no fluid inclusions were detected using cryo-TEM. Aqueous fluid would have once been present in these inclusions but is now missing not only by mechanical disturbance but also due to terrestrial weathering after falling to Earth (e.g., Nakamura et al. 2023).

### Remaining Questions and Future Work

See the Supplementary text.

## Isotope Tracers

### Oxygen Isotopes

Oxygen has three stable isotopes: ^16^O (99.757%), ^17^O (0.038%) and ^18^O (0.205%). Oxygen-isotope compositions are expressed in delta units relative to the Vienna Standard Mean Ocean Water (VSMOW, ^17^O/^16^O = 0.0003829 and ^18^O/^16^O = 0.0020052). The deviation from the TFL, a line of slope ∼0.52 defined by common terrestrial solids and waters (Robert et al. 1992), is expressed as $\Delta ^{17}\text{O} = \delta ^{17}\text{O} - 0.52 \times \delta ^{18}\text{O}$. Hence, mass-dependent variations are characterized by constant $\Delta ^{17}\text{O}$ while mass-independent fractionation presents variable $\Delta ^{17}\text{O}$. In the three-oxygen isotope diagram, $\delta ^{17}\text{O}$ *vs*. $\delta ^{18}\text{O}$, anhydrous chondritic constituents (e.g., CAIs, amoeboid olivine aggregates, chondrules) plot along a slope-1 line, referred to as the Primitive Chondrule Minerals line (PCM; Ushikubo et al. 2012). This line is thought to represent mixing between ^16^O-depleted (H_2_O) and ^16^O-enriched (CO) reservoirs in the protoplanetary disk, which may have been produced by CO self-shielding by UV light in the protosolar molecular cloud and/or in the outer part (>5 AU) of the protoplanetary disk (Yurimoto and Kuramoto 2004; Lyons and Young 2005). A striking feature of most Solar System materials, excluding refractory inclusions and so-called cosmic symplectite, is that their O-isotope compositions cluster closer to the TFL ($\Delta^{17}\text{O}∼0‰$) than to the solar composition ($\Delta^{17}\text{O}∼-29‰$; McKeegan et al. 2011). Potential explanations include: (i) the initial silicates inherited from the molecular cloud had a bulk $\Delta^{17}\text{O}∼0‰$ (Krot et al. 2010), or (ii) the bulk composition of the initial silicates and water ices had $\Delta^{17}\text{O}∼0‰$ and this composition has imprinted on solar materials by thermal processing in regions with elevated (dust + ice)/gas ratios (Alexander et al. 2017b) and/or because H_2_O isotopically exchanged with amorphous interstellar silicates much faster than CO (Yamamoto et al. 2018).

#### Bulk Compositions

**Fig. 6:**
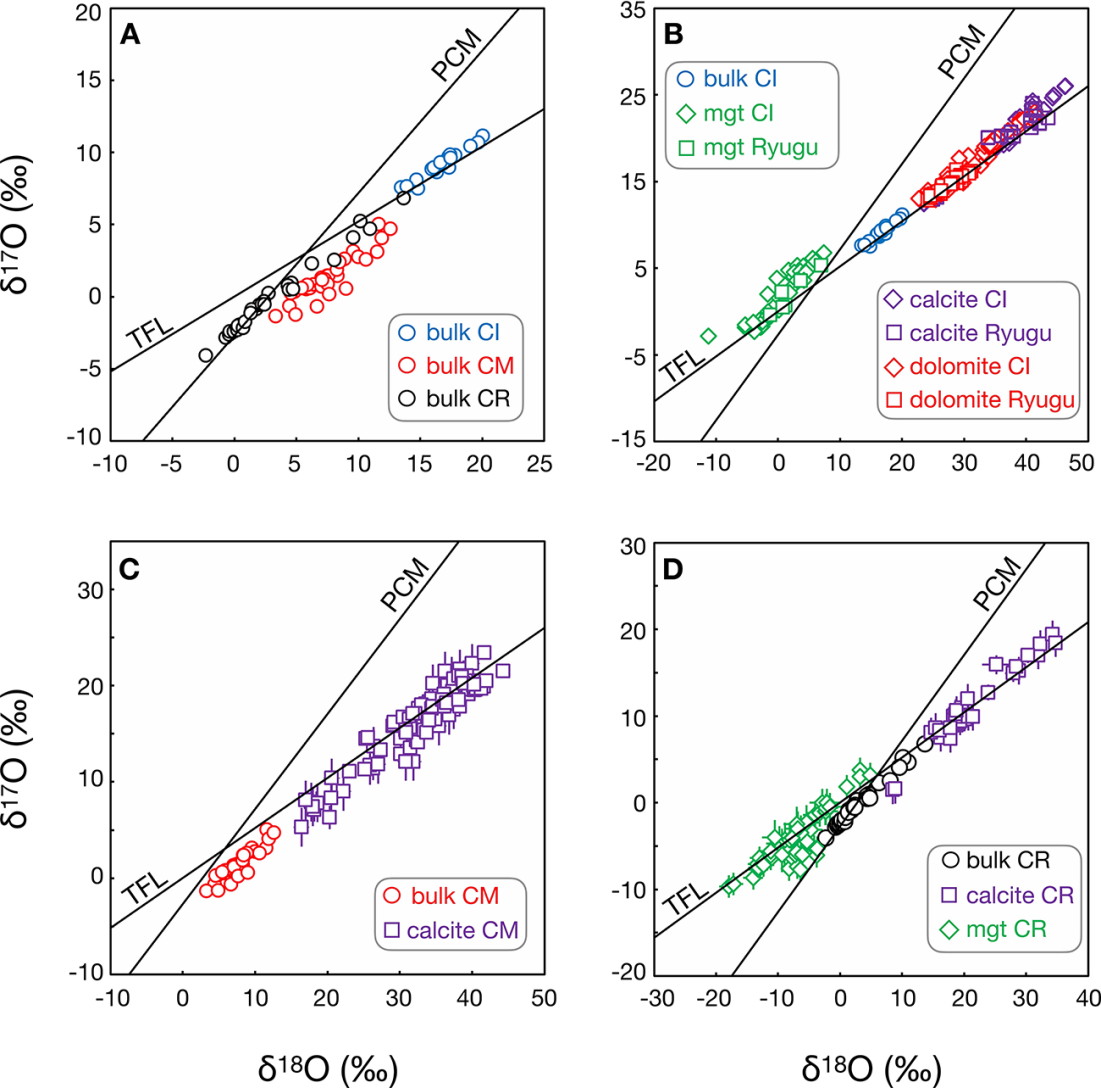
A) Oxygen three-isotope plot for bulk CI (including Ryugu), CM and CR chondrites. Data from Clayton and Mayeda (1999), Schrader et al. (2011), Hewins et al. (2014), Yokoyama et al. (2023). B) Oxygen-isotope composition of bulk CI chondrites and Ryugu samples and their secondary minerals (calcite, dolomite, magnetite (mgt)). Data from Clayton and Mayeda (1999), Piralla et al. (2020), Nakamura et al. (2022), Yokoyama et al. (2023), McCain et al. (2023), Fujiya et al. (2023). C) Oxygen-isotope compositions of bulk CM chondrites and representative isotopic compositions of CM calcite grains. Data from Clayton and Mayeda (1999), Lindgren et al. (2017), and Vacher et al. (2019a). D) Oxygen-isotope compositions of bulk CR chondrites and CR calcite and magnetite grains. Data from Clayton and Mayeda (1999) and Jilly-Rehak et al. (2018). Published carbonate data are listed in Table S1

In a plot of $\delta ^{17}\text{O}$ *vs* $\delta ^{18}\text{O}$ (Fig. 6A), whole rock and matrix samples of hydrated chondrites (i.e., CR, CM, CI, C1-ungrouped like Flensburg, and C2-ungrouped like Adelaide or MAC 87300) show significant departure from the line of slope 1 defined by unaltered refractory inclusions and chondrules. CI and C2-ungrouped chondrites show narrow O-isotope variations falling on or near the TFL and are higher in both $\delta ^{17}\text{O}$ and $\delta ^{18}\text{O}$ than anhydrous silicates and oxides in these meteorites (Fig. 6A; Clayton and Mayeda 1984, 1999; Ushikubo and Kimura 2021; Marrocchi et al. 2021b; Kawasaki et al. 2022; Morin et al. 2022). Furthermore, CM and CR chondrites with varying degrees of alteration have O-isotope compositions defining lines of intermediate slopes between the PCM and the TFL (Fig. 6A; Clayton and Mayeda 1984, 1999; Schrader et al. 2011; Marrocchi et al. 2018).

These continuous isotopic trends with extent of alteration do not correspond to the primary characteristics established during disk evolution but relate to secondary alteration processes that took place in asteroidal settings (Clayton and Mayeda 1984). They reveal that the main process controlling the O-isotope compositions of CM and CR chondrites is dictated by different W/R ratios involving ^16^O-rich anhydrous silicates and ^17,18^O-rich fluids (Clayton and Mayeda 1984, 1999; Brearley 2006; Zolensky et al. 2008; Krot et al. 2015; Marrocchi et al. 2018; Alexander 2019). This generates bulk O-isotope compositions that are progressively enriched in ^17,18^O with increasing degree of alteration. Assuming anhydrous chondritic protoliths plot along the PCM line, the CM and CR secondary isotopic trends offer the possibility to estimate the initial O-isotope composition of their respective anhydrous protoliths. Such a method implied initial bulk $\Delta ^{17}\text{O}$ values in the CM and CR protoliths of −4.5±0.6‰ and −2.1±0.6‰, respectively (Piralla et al. 2020) probably representing different chondrule populations sampled by these chondrites (Tenner et al. 2015; Marrocchi et al. 2022, 2024).

#### *In situ* Analysis of Aqueously Formed Minerals

The proportions of the original components of chondritic parent bodies (refractory inclusions, chondrules, Fe-Ni metal beads, sulfides, fine-grained matrix, and water ices) are highly variable between different chondrite groups. Heat released from the decay of short-lived ^26^Al led to the melting of the water ice, resulting in the establishment of prolonged fluid alteration processes within asteroids commencing shortly after their accretion (Sect. 9). Aqueous fluids affected the anhydrous primary chondritic components, leading to the formation of a large spectrum of secondary minerals whose nature is dependent on the physicochemical conditions and the duration of alteration (Sects. 2, 3, 6). Although the nature and abundances of secondary phases vary among CR, CM, CI, C1- and C2-ungrouped chondrites, they share two minerals whose O-isotope compositions can be analyzed using bulk techniques (Grady et al. 1988; Alexander et al. 2015) and/or *in situ* measurements by secondary ion mass spectrometry (SIMS): carbonate (Table S1) and magnetite.

In CI chondrites and Ryugu samples, Ca-carbonate grains (calcite and dolomite) plot on or slightly above the TFL and have higher $\delta ^{18}\text{O}$ compared to the bulk values (Fig. 6B; Piralla et al. 2020; Nakamura et al. 2022; Yokoyama et al. 2023; Fujiya et al. 2023; McCain et al. 2023). On average, dolomites are more homogeneous and have lower $\delta ^{18}\text{O}$ values than the calcites ($\delta ^{18}\text{O}_{\text{average}}$ of 30.2±4.4‰ *vs*. 37.2±6.6‰, respectively; Piralla et al. 2020; Nakamura et al. 2022; Yokoyama et al. 2023; Fujiya et al. 2023; McCain et al. 2023). Magnetite grains are ^16^O-enriched compared to bulk values (i.e., $\Delta^{17}\text{O}=1.70\pm 0.05‰$; Clayton and Mayeda 1999) and were historically considered as out of equilibrium with carbonates (i.e., having different $\Delta ^{17}\text{O}$; Clayton and Mayeda 1999). However, the recent isotopic appraisal of Ryugu samples has revealed a less clear situation: calcite, dolomite and magnetite grains have $\Delta ^{17}\text{O}$ possibly indicating isotopic equilibrium within uncertainty (Piralla et al. 2020; Nakamura et al. 2022; Yokoyama et al. 2023; Fujiya et al. 2023; McCain et al. 2023), although microstructural features of some magnetite grains indicate that they formed under disequilibrium conditions (Dobrică et al. 2023).

In CM and CR chondrites, Ca-carbonates show large isotopic variations ($\delta ^{18}\text{O}$ varying by more than 30‰ plotting on either side of the TFL). Interestingly, in both chondrite groups, carbonates extend the trends defined by bulk rocks and matrices (Fig. 6C, D; Benedix et al. 2003; Tyra et al. 2012, 2016; Fujiya et al. 2015; Verdier-Paoletti et al. 2017, 2019; Jilly-Rehak et al. 2017; Vacher et al. 2016, 2017, 2018, 2019a). These continuous trends confirm that O-isotope exchange between ^16^O-rich anhydrous silicates and ^17,18^O-rich water controls the O-isotope evolution of bulk CMs and CRs, and their respective secondary alteration minerals (Clayton and Mayeda 1984, 1999; Marrocchi et al. 2018). In such a scheme, ^17,18^O-rich secondary minerals correspond to early precipitates from alteration fluids that had suffered the least from O-isotope exchange with anhydrous silicates. Conversely, lower $\Delta ^{17}\text{O}$ values indicate that the carbonates formed following significant alteration, and as the O-isotope compositions of fluids evolved toward the O-isotope composition of anhydrous silicates and fluid (Verdier-Paoletti et al. 2017).

Magnetite grains are ubiquitous alteration products in CR matrices (Weisberg et al. 1993; Fig. 6D). Their O-isotope compositions plot along the TFL (with the notable exception of those in the CR MIL 09292 that have $\Delta^{17}\text{O}∼-3‰$) with $\delta ^{18}\text{O}$ ranging from −18‰ to +5‰ (Jilly-Rehak et al. 2017). CM chondrite magnetite grains have similar $\delta ^{18}\text{O}$ values ranging from −14‰ to +4‰ (Telus et al. 2019). A few CR and CM chondrites have magnetite and Ca-carbonates plotting on a single mass-fractionation line possibly allowing the equilibrium temperature to be estimated (Jilly-Rehak et al. 2017; Marrocchi et al. 2018; Telus et al. 2019). The bulk O-isotope measurements of magnetite grains separated from CIs show similar $\Delta^{17}\text{O}∼+1.5‰$ (Rowe et al. 1994) as those determined by SIMS (Nakamura et al. 2022; Yokoyama et al. 2023; McCain et al. 2023).

Of note, *in situ* SIMS measurements of the O-isotope compositions of carbonates are affected by complex matrix effects that require specific sets of standards, especially for quantifying the effects of minor elements (i.e., up to −0.3‰ per wt.% MgO for carbonates poor in Fe, Mn, Rollion-Bard and Marin-Carbonne 2011; Śliwiński et al. 2017). Assessing this potential instrumental bias is not straightforward as: (1) carbonates are rarely chemically and isotopically homogeneous, and (2) the instrumental bias is non-linear as a function of the concentrations of minor elements. These specificities are sources of uncertainties and call for the development of well-characterized carbonate standards for precisely determining the O-isotope compositions. This is particularly true for breunnerite, a rare Mg-Fe-carbonate commonly observed in CI chondrites. In addition, SIMS measurements of magnetite grains are affected by crystal orientation effects that are not always taken into account when measuring their O-isotope compositions (Huberty et al. 2010).

#### Oxygen Isotope Compositions of Carbonaceous Chondrite Water Ices

Determining the initial O-isotope compositions of water-ice grains accreted by chondritic asteroids is not an easy task as water-rock interactions continuously modified the O-isotope compositions of altering fluids. Although several approaches have been attempted for estimating the initial water ice compositions, most have focused on CM chondrites as bulk and Ca-carbonate compositions define a trend with varying $\Delta ^{17}\text{O}$ values.

Based on bulk O-isotope compositions and a two-stage model of gas-solid isotopic exchange, Clayton and Mayeda (1984, 1999) estimated the $\delta ^{18}\text{O}$ and $\Delta ^{17}\text{O}$ of the primordial CM water to be ∼30.3‰ and ∼4.4‰, respectively. A different estimate for the initial CM water composition was based on mass-balance calculations ($\delta^{18}\text{O}=55‰$ and $\Delta^{17}\text{O}=6.4‰$; Fujiya 2018). Estimates of the O-isotope composition of fluid at the time of phyllosilicate formation and carbonate precipitation were also reported using (1) clumped-isotope thermometry of CO_2_ ($\delta ^{18}\text{O}$ ranging from 2‰ to 8.1‰ and $\Delta ^{17}\text{O}$ ranging from −1.4‰ to −0.5‰; Guo and Eiler 2007) and (2) water extracted under stepped pyrolysis ($\delta^{18}\text{O}=11.5‰$ and $\Delta^{17}\text{O}=0.6‰$; Baker et al. 2002). Alexander (2019) proposed that all CCs accreted ices with a similar O-isotope composition of about $\delta^{18}\text{O}=30.9‰$ and $\Delta^{17}\text{O}=3.5‰$. Interestingly, such estimates define a line ($\text{R}^{2} = 0.987$; Fig. S4) with a slope that is indistinguishable, within error, from the bulk-matrix-carbonate trend, but with a different intercept (Verdier-Paoletti et al. 2017). This implies that the initial O-isotope composition of CM water was likely ^17,18^O-rich and does not plot along the slope-1 line but is shifted to the right from it. To the first order, this appears inconsistent with a self-shielding scenario, but the meaning is uncertain and needs to be investigated further.

#### Thermal Conditions of Aqueous Alteration and Water/Rock Ratio

Secondary minerals, such as carbonate and magnetite, represent direct proxies of the asteroidal fluids from which they formed and can, in theory, be used to decipher their formation temperatures (Clayton and Mayeda 1984). However, determining these temperatures requires knowledge of the O-isotope compositions of their parental fluids, which itself requires knowledge of the carbonate/magnetite precipitation temperatures, leading to a seemingly circular problem. This issue can be overcome when secondary phases with similar $\Delta ^{17}\text{O}$ are present in a given chondrite, which allow alteration temperatures to be estimated (assuming equilibrium that is not so straightforward). This approach led to (1) contrasting estimates of alteration temperatures of CM chondrites ranging from 0-20 °C (Benedix et al. 2003) to 80–120 °C (Baker et al. 2002) and (2) more coherent results for CR chondrites with temperatures of 55–90 °C (Jilly-Rehak et al. 2018) and 35–110 °C (Marrocchi et al. 2018). A similar approach for the CI-related Ryugu samples gave estimates of ∼0-80 °C (Yokoyama et al. 2023; McCain et al. 2023; Nakamura et al. 2022; Kita et al. 2024). Other estimates for CM chondrites are based on (1) clumped-isotope thermometry of CO_2_ ($\Delta ^{47}$, which corresponds to anomalous enrichments of mass 47 (i.e., $^{13}\text{C}$^18^O^16^O in CO_2_ derived from H_3_PO_4_ digestion of carbonates, Ghosh et al. 2006): 20–70 °C (Guo and Eiler 2007), ∼5–51 °C and ∼75–101 °C for calcite and dolomite, respectively (Clog et al. 2024), (2) bulk $\delta ^{13}\text{C}$ and $\delta ^{18}\text{O}$ trends in carbonates: 0–130 °C (Alexander et al. 2015), and (3) the distance between the respective trends defined by CM water and bulk + carbonates: 50–300 °C (Verdier-Paoletti et al. 2017; Vacher et al. 2019b). Discrepancies could in part be due to the lack of standard materials with compositions perfectly matching the chondritic minerals to correct the SIMS instrumental mass fractionation or relate to the possibly incorrect estimate of equilibrium between components used for the temperature calculation.

Water/rock ratios of chondritic parent bodies can be estimated by combining bulk and mineral O-isotope data (Alexander 2019 and references therein). These estimates indicate variable W/R mass ratios for hydrated chondrite groups with CR = 0.10–0.40, CM = 0.10–0.40, and CI = 0.38–0.43 (Alexander 2019 and references therein).

### Hydrogen Isotopes

#### Bulk Compositions

Hydrogen has two stable isotopes ^1^H (99.9885%) and ^2^H (deuterium: D) (0.0115%). The H-isotope composition is reported in the delta notation relative to the SMOW (D/H = 0.00015576). Bulk H-isotope compositions have been measured in a large range of phyllosilicate-rich chondrites including CR, CM, CI, and C1- and C2-ungrouped CCs either by step heating (e.g., Robert and Epstein 1982; Eiler and Kitchen 2004) or using an elemental analyzer coupled with an isotope ratio mass spectrometer (EA-IRMS) allowing permil-level measurements on aliquots of a few milligrams (e.g., Alexander et al. 2012, 2018; Vacher et al. 2020; Marrocchi et al. 2023b). In water-rich chondrites, bulk $\delta $D values range from −300‰ in the ungrouped C1 chondrite Flensburg (Bischoff et al. 2021) to >+600‰ in CRs and some C-ungrouped chondrites (see McCubbin and Barnes 2019; Piani et al. 2021 for compilations).

**Fig. 7:**
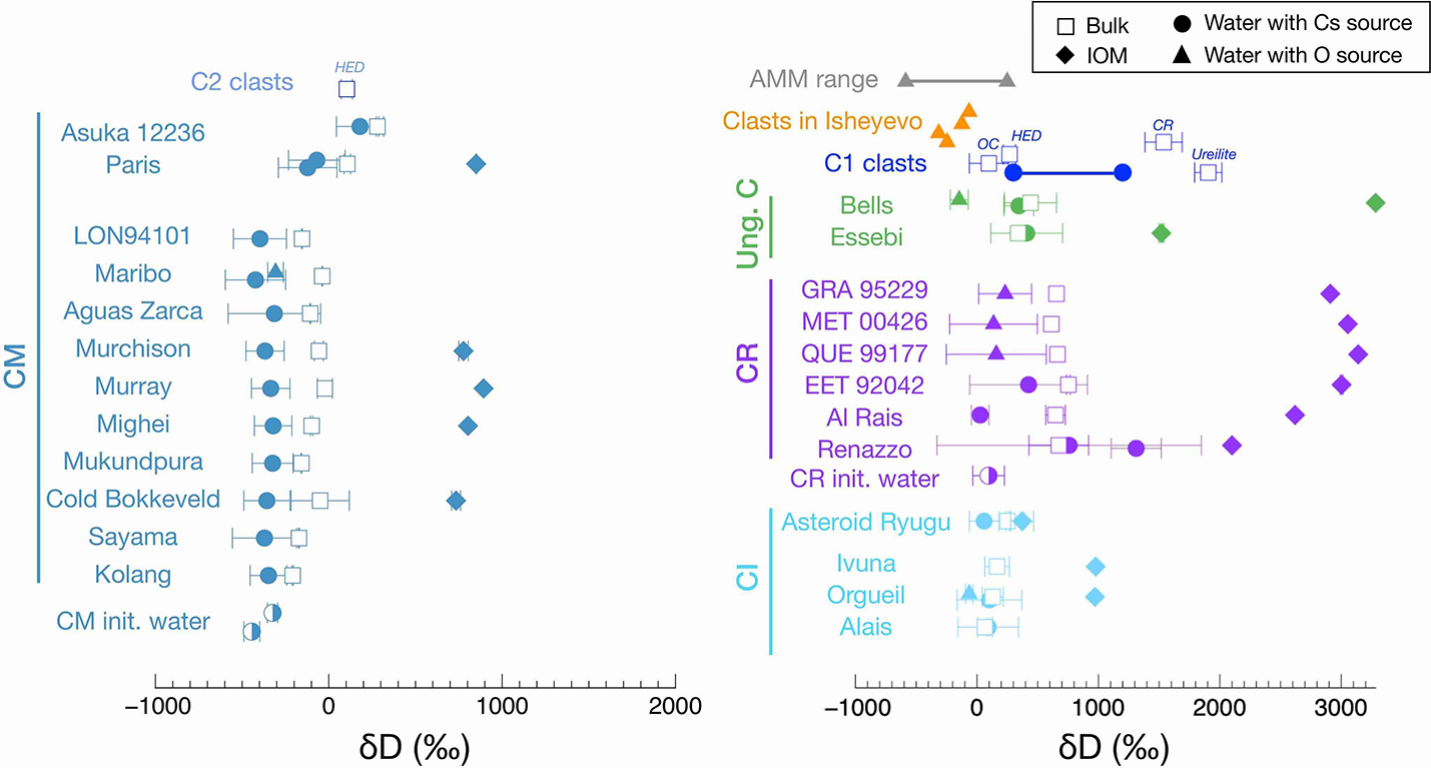
Comparison of the H-isotope composition (expressed in delta notation against standard mean ocean water, SMOW) measured in bulk, IOM and hydrous silicates in hydrated CR, CM, CI, and ungrouped CCs, hydrated clasts, Ryugu samples and Antarctic micrometeorites (AMMs). Hydrous silicate data were obtained by SIMS using two different measurement protocols, with an O (triangle symbols) and a cesium (round symbols) primary beam, respectively. CM chondrites are listed from the more altered at the bottom to the least altered to the top. References are in Table S2 in the supplementary materials. For AMMs and water in C1 clasts, the symbols and horizontal line indicates the range of $\delta $D values. The host meteorites of the C1 and C2 clasts are indicated above the corresponding symbol (for more detailed see Table S2)

In CM and CR chondrites, bulk H-isotope measurements reveal the presence of positive and distinct correlations between the $\delta $D values and C/H ratios (Fig. S5A; Alexander et al. 2012). These correlations were interpreted to reflect mixing lines between a D-poor and C-free component (presumably the water from which the hydrated minerals formed) with D-rich organic matter. The H-isotope intercepts were used to estimate the $\delta $D of the initial water components accreted by the CM and CR parent bodies, which are several hundreds of permil lower than the bulk compositions (Alexander et al. 2012; Fig. 7 and Table S2). The bulk H-isotope analyses performed after degassing to remove atmospheric water (Vacher et al. 2020; Lee et al. 2021) produce a CM mixing line in $\delta $D versus C/H space that is consistent with the existing IOM being the C-rich and D-rich endmember of the mixture (Fig. S5B; Marrocchi et al. 2023b). This result suggests that only limited isotopic exchange occurred during aqueous alteration in CM chondrites (a few tens of permil at most) and that the primary H-isotope signatures in IOM and water were preserved during alteration (Remusat et al. 2010; Marrocchi et al. 2023b). On the other hand, the H-isotope variations observed among CM IOM (ranging from ∼800‰ to ∼1200‰; Alexander et al. 2010) cannot be explained by this model, and either require that there was H_2_O-IOM isotopic exchange or heterogeneity in the IOM accreted by CMs.

#### Hydrogen Isotopes in Hydrated Minerals

Hydrogen isotopes are powerful tracers of the origin of chondritic water-ice and organic molecules, as those inherited from the molecular cloud should be enriched in D by ∼2–3 orders of magnitude relative to those formed or re-equilibrated during the evolution of the protoplanetary disk (Ceccarelli et al. 2014). However, direct measurements of $\delta $D signatures in water or organics are challenging because chondrites reflect complex mixtures of organics and hydrated minerals that cannot be physically separated. *In situ* analyses at the micrometer-scale by SIMS allows for determination of the approximate H-isotope compositions from hydrated minerals to be obtained using two different analytical protocols.

A first protocol proposed by Deloule and Robert (1995) takes advantage of the preferential emission of H^+^ and D^+^ from hydrated silicates relative to organics when using a O^−^ source for sputtering the sample surface (at least one order of magnitude lower; Bonal et al. 2010). Due to the large proportions of H-rich minerals in the matrix of aqueously altered chondrites, the $\delta $D values measured in this way should approximate the bulk compositions of hydrated minerals (Fig. 7 and references therein). However, the C content should be monitored to avoid the risk of local contributions from D-rich organics to the measured $\delta $D values.

With a Cs^+^ primary beam, the difference of H^−^ and D^−^ emissions between hydrated silicates and organics is less pronounced (Bonal et al. 2010; Piani et al. 2012) and thus both phases contribute to the measured $\delta $D. The second protocol is based on the simultaneous measurement of the $\delta $D and C/H ratios. Depending on the location of the measurement in the chondrite matrix, the $\delta $D and C/H ratios vary with the relative amounts of hydrated minerals and organic matter defining a mixing line between a D-poor, C-free endmember (hydrated minerals) and a D-rich and C-rich organic endmember (Piani et al. 2018). The extrapolation to the C-free endmember (C/H = 0) allows the $\delta $D of hydrated minerals to be estimated without H contributions from adjacent organics (Fig. 7 and references therein). The limitation of this protocol is the large error (±100‰ for a given chondrite $\delta $D) coming from extrapolations to the zero intercepts. Additionally, the H-isotope compositions of individual coarse-grained hydrated silicates and chondrule glasses were measured in CR chondrites using a Cs^+^ ion beam (Bonal et al. 2013; Shimizu et al. 2021).

Chondrites measured by the two protocols are in good agreement implying that the two techniques produce reliable results (Fig. 7). Nonetheless, the H-isotope measurements by SIMS in chondrites would gain in accuracy with the development of hydrated silicate standards covering the large range of D/H variations present in chondritic minerals (several hundred of ‰). Except for CI chondrites, for which hydrated minerals dominate the H budget, the $\delta $D values of hydrated minerals are systematically lower than bulk and IOM (Fig. 7). Like for IOM (e.g., Alexander et al. 2017b), distinct $\delta $D values are observed for water in the different chondrite groups with δD∼−350‰ in CM, ∼+100‰ in CI and CR, and ∼+400‰ for the ungrouped Bells and Essebi (Alexander et al. 2012; Piani et al. 2021).

Hydrated clasts have been identified in the majority of chondrite and achondrite groups as C1-like and C2 objects that underwent aqueous alteration in their own parent bodies and possibly also in their host bodies (Sect. 3). Hydrogen-isotope studies of C1 dark clasts in ordinary chondrites (OC), HED meteorites, ureilites, CR and CH/CB chondrites have been carried out by NanoSIMS using primary O^−^ and Cs^+^ sources. Patzek et al. (2020) found high bulk $\delta $D values for the aforementioned clasts (300–2000‰), relative to most of the other CCs (Fig. 7). The ureilite and CR clasts are among the most D-rich bulk Solar System materials, and their anomalous H-isotope signatures have been interpreted as reflecting the accretion of abundant D-rich ices and organics relative to CCs. Like bulk values, the ureilite and CR clast water (δD∼300–1200‰) derived from the zero intercepts of correlations between D/H and Si/H or C/H ratios are significantly increased relative to CCs. In contrast, Isheyevo lithic clasts that contain the highest ^15^N-enrichments found in Solar System materials and indicative of outer Solar System signatures (Briani et al. 2009; Bonal et al. 2010; van Kooten et al. 2017a), have phyllosilicate compositions that are comparable to other CCs (δD∼−100/−300‰; van Kooten et al. 2017a; Fig. 7).

In CM chondrites, for which samples with different degrees of aqueous alteration are available, the $\delta $D values measured *in situ* for hydrated minerals are independent to the extent of aqueous alteration, indicating the CM chondrites accreted D-poor water ice with δD∼−350‰ (Fig. 7). Certain regions of Paris and Asuka 12236 are the least altered CM lithologies (Hewins et al. 2014; Kimura et al. 2020) and are amongst the exceptions in displaying unusually D-rich H-isotope compositions possibly reflecting the presence of another H-bearing component in addition to organics and water (Piani et al. 2018; Marrocchi et al. 2023b). Hydrated amorphous silicates, that are ubiquitous in these two chondrites, were proposed as a H-bearing component that could retain H acquired before the onset of CM parent-body alteration (Marrocchi et al. 2023b).

#### Origin of H-Isotope Variations of Organic Matter and Water Among Chondrite Groups

The highly variable H-isotope signatures of hydrated minerals and organics among chondritic materials (Fig. 7) can be interpreted in two ways. They can either be the result of secondary processes occurring in asteroids and disturbing the initial isotope signatures, or they reflect the heterogeneous distributions and compositions of water ice and organics in the protoplanetary disk at the time of asteroid accretion. In asteroids, the accreted ice, once melted, percolates among the solid phases. The main process thought to have affected the H-isotope composition in H-rich chondrites is H-isotope exchange between D-rich organic components and D-poor aqueous fluids (e.g., Alexander et al. 2010). Such exchanges are rapid and almost complete in laboratory experiments conducted at ≥250 °C, resulting in a decrease of the IOM H/C and $\delta $D in the presence of D-poor water (Yabuta et al. 2007; Oba and Naraoka 2009; Kebukawa et al. 2021; Foustoukos et al. 2021) (Sect. 5.4). A progressive decrease of IOM H/C and $\delta $D has been observed in lithologies of the C2 Tagish Lake that experienced varying degrees of aqueous alteration and elevated temperatures for relatively short periods of time (Alexander et al. 2014). However, no evidence for H-isotope exchange between organic particles and water retained in the surrounding hydrated minerals was observed at the micrometer scale in the matrix (Remusat et al. 2010) nor H-isotope evolution in CM chondrites of increasing alteration degrees (Fig. 7). Temperatures might explain the differences in exchange efficiency for water and organics in laboratory experiments performed above 150 °C (Oba and Naraoka 2009; Kebukawa et al. 2021; Foustoukos et al. 2021). The experimentally determined D–H exchange between organic matter and water being relatively fast, alteration temperatures close to 0 °C would likely be necessary to maintain H-isotope disequilibrium between organic matter and water for millions of years (Kebukawa et al. 2021). These low-temperatures are at odd with the peak temperatures commonly estimated for aqueous alteration (50–300 °C; Sect. 8.1.4); the discrepancy between experiments and natural samples constitute a major issue for the interpretation of the isotopic data.

If not resulting from asteroidal processes, the variable H-isotope signatures of organics and water in the different chondrites could reflect an isotopically heterogeneous distribution in the protoplanetary disk, possibly inherited from multiple episodes of infall from the surrounding molecular cloud to the disk (Piani et al. 2021; Kuznetsova et al. 2022). Because the preservation of D-rich interstellar water and/or organics in the disk is only possible below their respective sublimation temperatures (160 K and 350–450 K, respectively), the H-isotope signatures of chondritic materials could depend on both the timing and location at which their parent asteroids formed, both varying with the thermal evolution of the disk (Yang et al. 2013; Jacquet and Robert 2013).

### Carbon, Nitrogen, and Sulfur Isotopes

#### Carbon Isotopes in Carbonates and Conditions of Parent Body Alteration

The bulk C- and O-isotope compositions of carbonates in CI chondrites have been measured for Orgueil and Ivuna (Grady et al. 1988; Alexander et al. 2015). The data are consistent with, on average, the carbonates having formed from a single fluid at temperatures of ∼0–40 °C (Alexander et al. 2015). From *in situ* measurements obtained in Ivuna and Ryugu samples, calcites show large variations for C ($\delta^{13}\text{C}=65\text{–}108‰$ relative to the Vienna Peedee Belemnite; $^{13}\text{C}/^{12}\text{C} = 0.01123720$) and O-isotopes (Sect. 8.1.2), while dolomite is more homogeneous ($\delta^{13}\text{C}=65\text{–}75‰$; Fujiya et al. 2023). The isotope variations in calcite are thought to result from their early precipitation over a large range of prograde temperatures and of $f$O_2_ associated with the evolution of the proportions of CH_4_, CO, and CO_2_ in the fluid. In contrast, the isotopically homogeneous dolomite grains seem to have formed later, possibly during retrograde cooling, from a fluid that was close to equilibrium with a CO_2_-dominated gas (Fujiya et al. 2023).

In CM chondrites, C-isotopes in carbonates measured either by bulk techniques (Grady et al. 1988; Alexander et al. 2015; Tyra et al. 2016; Vacher et al. 2020), SIMS (Lee et al. 2013; Fujiya et al. 2015; Vacher et al. 2017; Telus et al. 2019) or using clumped isotopes (Sect. 8.1.4). Guo and Eiler (2007) show a large range of $\delta ^{13}\text{C}$, from ∼25‰ to ∼80‰. Positive correlations for $\delta ^{13}\text{C}$ and $\delta ^{18}\text{O}$ observed in bulk carbonates have been used to infer that on average they precipitated from a single fluid under variable temperatures of 0–130 °C that roughly correlate with extent of alteration for many but not all meteorites (Alexander et al. 2015). On the other hand, negative correlations for $\delta ^{13}\text{C}$ and $\delta ^{18}\text{O}$ observed using clumped isotopes of a smaller sample of meteorites have been interpreted as the result of the progressive ^13^C-enrichment in the fluid due to the loss of ^12^C-enriched CH_4_ gas (Guo and Eiler 2007). *In situ* measurements of individual carbonate grains performed by SIMS revealed a more complex picture with (1) calcite being more heterogeneous than dolomite, and (2) no systematic link between O- and C-isotope variations (Fig. S6). The latter possibly indicates that carbonates in CM chondrites probably result from several episodes of carbonate dissolution and reprecipitation (e.g., Tyra et al. 2012).

#### Carbon, Nitrogen, and Sulfur Isotopes: Implication for Understanding the Nature of Chondritic Water Ices

The range of $\delta ^{13}\text{C}$ values in chondrite carbonates can be related to the nature of the primary C-bearing precursors and could provide clues on the formation location of their parent bodies. The highest $\delta ^{13}\text{C}$ values measured in CI carbonates call for the presence of ^13^C-rich ices (Fujiya et al. 2023) that might have also been sampled by comets (Hässig et al. 2017). The ^13^C-rich compositions of ices, preserved in the colder regions of the Solar System, could originate from self-shielding processes during CO photodissociation in the solar nebula or its parent molecular cloud (e.g., Lyons et al. 2018). Similarly, the C2 Tagish Lake was found to contain both calcite and dolomite with $\delta ^{13}\text{C}$ values that were as high as in the CI chondrite carbonates (∼70‰; Fujiya et al. 2019), but lower than the higher values measured recently in Ryugu and Ivuna calcites (up to 108‰; Fujiya et al. 2023; Fig. S7). Associated with the unusually high abundance of carbonates (Grady et al. 2002), the isotopic composition of Tagish Lake could indicate its parent body accreted a relatively high proportion of ^13^C-rich CO_2_ ices and thus possibly formed beyond the CO_2_ snowline (Fujiya et al. 2019). However, precisely deducing the chondrite formation location based on the inferred amounts of CO_2_ in water ice is difficult because some amounts of CO_2_ can be trapped in H_2_O ice even at temperatures higher than the CO_2_ condensation temperature (e.g., Fayolle et al. 2012).

Soluble organic compounds have been proposed to be candidates for the C source of CM chondrite carbonates due to their comparable isotopic ranges (0–65‰; Vacher et al. 2017; Fig. S7). However, the high abundance of carbonates would require much more abundant soluble organics than measured in any chondrite (Aponte et al. 2016). ^13^C-rich ices could also be the source of CM carbonate C (Telus et al. 2019), but the $\delta ^{13}\text{C}$ composition of ice accreted by CMs would have to be less enriched in ^13^C than in CIs or Tagish Lake chondrites suggesting fractionation processes operating in the parent bodies and/or the sampling of different C-bearing ice reservoirs during accretion.

Differences in the S-isotope compositions of sulfides ($\delta^{34}\text{S}∼-2‰$ and +1‰ in CMs and CIs, respectively, relative to the Vienna-Canyon Diablo Troilite) were suggested to derive from the sampling of two distinct S reservoirs in CI and CM chondrites (Visser et al. 2019). The presence of mass-independent variations observed in the inorganic phases of CM chondrites are likely derived from photochemistry occurring in the nebula (Labidi et al. 2017). The heterogeneity of mass-independent S-isotope signatures among different CM chondrites and between fragments of the CM chondrite Murchison argues for heterogeneous accretion of at least two S-bearing components with distinct isotopic signatures (Labidi et al. 2017). Nonetheless, analysis of $\delta ^{34}\text{S}$ in a large suite of bulk chondrites (mainly CR and CM) and their IOM have revealed the absence of large, systematic variations in $\delta ^{34}\text{S}$ among chondrite groups or petrological types (Alexander et al. 2022).

In CM and CO chondrites, bulk $\delta ^{15}$N value decreases with increasing degree of alteration, likely due the progressive removal of an organic ^15^N-rich component during alteration (Pearson et al. 2006). The presence of extreme $\delta ^{15}$N variations in the hydrated clasts of the CB/CH chondrite Isheyevo with D-depletion compared to organic matter were proposed to derive from the heterogeneous accretion of NH_3_ and HCN-bearing ices during the cooling of the protoplanetary disk (van Kooten et al. 2017a). Such ice reservoirs do not appear to have been sampled by other water-rich CCs.

Overall, the isotopic compositions of H, C, O and, possibly, S could be consistent with the fact that the ices sampled by the parent bodies of the different chondrites originate from distinct reservoirs in the protoplanetary disk, but efforts must continue to quantify the influence of parent body processes on the isotopic signatures.

### Remaining Questions and Future Work

Studies coupling precise mineralogical observations and O-isotope analyses with proper standards (i.e., effects of trace elements) in alteration phases are needed to validate the use of the O-isotopes in estimating the temperatures of aqueous alteration in chondrites. *In situ* C-isotope analyses of CR carbonates would also be of help to characterize the alteration conditions of their parent body.

Efforts should be undertaken to reconcile the temperature estimates obtained from different geochemical constraints and relate them to the complex (prograde/retrograde) thermal history of the chondrite parent asteroids.

## Chronology of Aqueous Alteration

In this section, we provide an overview of the existing chronological data regarding low-temperature aqueous alteration of CR, CM, CI, and ungrouped carbonaceous chondrites, hydrated clasts in other meteorites, and surface materials of asteroid Ryugu. The chronology of aqueous alteration in these materials is crucial for constructing thermal evolution models of their parent bodies. Since the 1980s, it has been inferred from ^87^Rb-^87^Sr isotopic systematics that aqueous alteration is one of the earliest geological processes in the Solar System and took place within 100 Myr after its birth (Macdougall et al. 1984). Furthermore, the evidence for *in situ* decay of ^53^Mn, a short-lived radionuclide with a half-life of 3.7 Myr, in carbonate minerals in CI chondrites confirmed that there was water activity in their parent body within a few tens of Myr after the birth of the Solar System (Endress et al. 1996). Therefore, the chronology of low-temperature aqueous alteration can be best explored by using short-lived radionuclides, which normally provide higher temporal resolution than long-lived radionuclides. Thus, we here focus on the chronology using ^53^Mn-^53^Cr ($t_{1/2} = 3.7\text{ Myr}$) and ^129^I-^129^Xe ($t_{1/2} = 16.1\text{ Myr}$) decay systems.

### ^53^Mn-^53^Cr Chronology of Carbonates in CR, CM, CI, and Ungrouped Carbonaceous Chondrites, Hydrated Clasts in Other Meteorites, and Ryugu Samples

In general, radiometric dating of secondary minerals works well only if these minerals have high abundance ratios of parent/daughter nuclides resulting from the chemical fractionation during aqueous alteration. Therefore, the application of ^53^Mn-^53^Cr dating of low-temperature aqueous alteration is predominantly limited to carbonate minerals, which incorporate $\text{Mn}^{2+}$ into their crystal structure but hardly contain any $\text{Cr}^{3+}$.

Carbonate minerals can commonly be found in CR, CM, CI, and ungrouped carbonaceous chondrites, hydrated clasts in other meteorites, and Ryugu samples (e.g., Johnson and Prinz 1993; Riciputi et al. 1994; Endress and Bischoff 1996; Endress et al. 1996; Ivanova et al. 2008; Lee et al. 2013; Jilly-Rehak et al. 2017; Bischoff et al. 2021; Nakamura et al. 2023). Calcium carbonate (calcite or aragonite), Ca- and Mg-carbonate (dolomite), and Mg-, Fe-, and Mn-carbonate (breunnerite) are the most common carbonate minerals (Sect. 2). Their ^53^Mn-^53^Cr systematics record the timing of carbonate formation during aqueous alteration; later disturbance of ^53^Mn-^53^Cr systematics in carbonates by Mn and Cr diffusion is unlikely because the peak temperatures experienced by these meteorites (metamorphosed CI and CM chondrites are not discussed here) are low (<220–240 °C) (Busemann et al. 2007; Sect. 8). Notably, carbonates in CM chondrites often preserve zoning in Mn (and other minor elements) within single grains, indicating that the Mn was not homogenized after the carbonate formation (e.g., Lee et al. 2014; Fujiya et al. 2020).

**Fig. 8:**
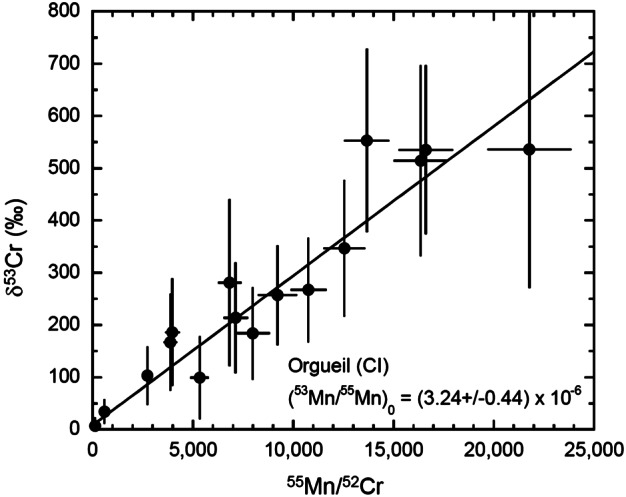
^53^Mn-^53^Cr isochron diagram of dolomite in the Orgueil CI chondrite. Note that the ^55^Mn/^52^Cr ratios were estimated using the RSF of a synthetic calcite standard (Sugiura et al. 2010), which may lead to systematic bias. Data from Fujiya et al. (2013)

Since the pioneering work by Endress et al. (1996), the ^53^Mn-^53^Cr ages of carbonates have been measured *in situ* using SIMS. For ^53^Mn-^53^Cr dating, ^53^Cr/^52^Cr and ^55^Mn/^52^Cr ratios must be measured, and the slope of the regression line of the data in ^53^Cr/^52^Cr *vs*. ^55^Mn/^52^Cr space (^53^Mn-^53^Cr isochron diagram) corresponds to the initial ^53^Mn/^55^Mn ratio [(^53^Mn/^55^Mn)_0_] when the Mn-Cr isotope system achieved closure (Fig. 8). The SIMS measurements of ^55^Mn/^52^Cr ratios are challenging because the so-called matrix effect of SIMS influences the observed ^55^Mn^+^/^52^Cr^+^ ion intensity ratios relative to the true ^55^Mn/^52^Cr ratios, the extent of which depends on the target material (matrix). Therefore, a matrix-matched standard material with a known ^55^Mn/^52^Cr ratio must be used to determine the relative sensitivity factor (RSF: the ^55^Mn^+^/^52^Cr^+^ ion intensity ratio divided by the true ^55^Mn/^52^Cr ratio) and to calculate the ^55^Mn/^52^Cr ratios of unknown samples from the observed ^55^Mn^+^/^52^Cr^+^ ion intensity ratio.

The importance of using a proper carbonate standard was not recognized by the earliest studies. Natural carbonates including those in meteorites contain little Cr, which makes the preparation of a proper carbonate standard difficult. Therefore, most work until the 2010s used silicate (e.g., San Carlos olivine: SCol) standards, leading to a systematic uncertainty in ^55^Mn/^52^Cr ratios, and therefore, in the (^53^Mn/^55^Mn)_0_ of carbonates. Sugiura et al. (2010) first showed that the RSF of synthetic Mn- and Cr-bearing calcite is ∼30% lower than that of SCol, implying that the ^53^Mn-^53^Cr ages obtained using SCol appear ∼2.2 Myr older than those obtained using the synthetic calcite standard.

In Fig. S8 and Table S3, we summarize the (^53^Mn/^55^Mn)_0_ of carbonates in CR, CM, CI, and ungrouped carbonaceous chondrites, hydrated clasts in the Isheyevo CH/CB-like meteorite, and Ryugu samples and their relative formation ages after CV CAIs ($\Delta t$_CAI_). Table S3 also includes the corresponding absolute ages and standard materials used. In Figure S8, the ages obtained using proper (i.e., matrix-matched) standards are shown in filled symbols. It should be noted that we calculated these absolute ages by using the U-corrected Pb-Pb age of 4563.37 ± 0.25 Ma (Brennecka and Wadhwa 2012) and the (^53^Mn/^55^Mn)_0_ of $(3.54 \pm 0.18) \times 10^{-6}$ (McKibbin et al. 2015) of the D’Orbigny angrite. Because ^53^Mn is a short-lived radionuclide, a time anchor with a known ^53^Mn/^55^Mn ratio and Pb-Pb absolute age is required to translate the inferred (^53^Mn/^55^Mn)_0_ to absolute ages. Therefore, the absolute ages of carbonates depend on the selection of a time anchor, which potentially introduces uncertainty. The above Pb-Pb age of D’Orbigny was obtained using measured ^238^U/^235^U ratios and seems to be the most reliable age among those reported so far. Another (^53^Mn/^55^Mn)_0_ of D’Orbigny, $(3.24 \pm 0.04) \times 10^{-6}$ was reported by Glavin et al. (2004). If we use this number as an anchor, the absolute ages of carbonates will be older by ∼0.5 Myr. The calculation of the time after CAI formation includes further complication; we need the absolute age of CAIs, which is still under significant debate (e.g., Desch et al. 2023). Here, we use the absolute age of 4567.30 Ma for CAI formation reported by Connelly et al. (2012) to calculate the $\Delta t_{\mathrm{CAI}}$ values.

An overview of the previously reported ^53^Mn-^53^Cr ages of carbonates in CR, CM, CI, ungrouped carbonaceous chondrites, hydrated clasts in other meteorites, and Ryugu samples, is described here. Calcite and dolomite in CM chondrites have been dated to ∼4–6 Myr after CV CAI formation (Fujiya et al. 2012), which is consistent with more recent data (4 ± 2 Myr after CV CAIs) for carbonates in CMs and CIs, and CM and CI clasts in other meteorite types (Visser et al. 2020). The ^53^Mn-^53^Cr ages for dolomite and breunnerite grains in CI chondrites range from ∼3–7 Myr after CV CAIs, with the petrography and intricate chemical zoning in carbonates recording how the composition of the fluid evolved as alteration progressed (Endress et al. 1996; Hoppe et al. 2007; Fujiya et al. 2013). The ^53^Mn-^53^Cr ages of carbonates in CR chondrites have been obtained for a small number of samples (Jilly-Rehak et al. 2017). This is a challenging task, because carbonates in CR chondrites are much rarer than in CM chondrites, tend to be significantly smaller, and are all calcite with low concentrations of Mn. However, relatively large polycrystalline calcite grains, up to 100 μm in size, occur in Renazzo and grains of Mn-bearing dolomite occur in dark inclusions and in the CR1 chondrite GRO 95577. Jilly-Rehak et al. (2017) showed that calcite in Renazzo, and a dolomite in a dark inclusion from Renazzo, show ^53^Cr excesses, consistent with the presence of live ^53^Mn during crystallization of both carbonates. These excesses indicate crystallization ages of ∼4–5 Myr after CV CAIs, very similar to ages measured for CI and CM chondrite carbonates. In contrast, dolomite in GRO 95577 is significantly younger with formation ages of ∼12 Myr after CV CAIs, indicating that hydrothermal systems could be maintained for extended durations on the CR chondrite parent body. Calcite and dolomite in Flensburg formed simultaneously at 2.7 ± 1.0 after CV CAIs, making them, together with calcite in the Jbilet Winselwan heated CM chondrite (∼2.6 Myr after CV CAIs), the oldest ^53^Mn-^53^Cr ages yet measured for chondrites. The ^53^Mn-^53^Cr ages of carbonate grains in Ryugu samples obtained in different laboratories are not consistent: Yokoyama et al. (2023) and Nakamura et al. (2022) obtained formation ages of ∼3–6 Myr after CV CAIs for dolomite, which is largely consistent with ages for carbonates in CR, CM, CI chondrites, whereas McCain et al. (2023) reported ages as old as ∼0.4 Myr after CV CAIs.

There are few ^53^Mn-^53^Cr ages that have been obtained using proper standards (Fig. S8). In particular, most Mn-Cr ages of dolomite have been estimated without using a dolomite standard, because the laboratory synthesis of Mn- and Cr-bearing dolomite is more difficult than that of calcite (Dunohue et al. 2019). Another approach to produce carbonate standards is Cr-ion implantation into natural carbonates by a particle accelerator (Steele et al. 2017; McCain et al. 2020). A few dolomite ages have been obtained using carbonate standards with implanted Cr, and they appear somewhat older than those obtained with synthetic calcite standards. Notably, two ages of Ryugu carbonates obtained using carbonate standards with implanted Cr are very old (4566.9 Ma or 0.4 Myr after CAIs; McCain et al. 2023). The validity of these ages will be discussed in the supplementary materials and should be examined in future work.

The ^53^Mn-^53^Cr ages of carbonates obtained with proper standards in carbonaceous chondrites discussed here range from 3 to 5 Myr after CV CAIs (Fig. S8). These observations indicate that carbonate formation (and possibly, the onset of aqueous alteration) was almost simultaneous among water-rich planetesimals. This result, in turn, implies that these bodies accreted almost simultaneously. Fujiya et al. (2012, 2013) argued that CM and CI chondrite parent bodies formed 3–4 Myr after CV CAIs based on a thermal evolution model where the energy of ^26^Al decay was taken into account as the heat source. On the other hand, the old ages of Ryugu carbonates (McCain et al. 2023) suggest that the Ryugu parent body may have accreted very early, within 0.4 Myr of CV CAIs. Early formation of the Ryugu parent body must have resulted in high peak temperatures. However, Ryugu samples preserve phyllosilicates without apparent signatures of dehydration and have not been heated above ∼100 °C (Yokoyama et al. 2023; Nakamura et al. 2023). This discrepancy may be resolved if the Ryugu parent body was smaller than ∼20 km in diameter, or if it was larger than ∼20 km in diameter but disrupted before the internal temperatures rose to the dehydration temperature (McCain et al. 2023). Another scenario would be fluid convection which may have occurred within the Ryugu parent body and efficiently transferred internal heat to its outer regions (e.g., Palguta et al. 2010). In any case, these arguments demonstrate that the Mn-Cr ages of carbonates provide important insights into the formation age, size, and thermal history of water-rich planetesimals in the early Solar System.

### ^129^I-^129^Xe Chronology of Magnetite in CI Chondrites

The ^129^I-^129^Xe chronometer is based upon the decay of now-extinct ^129^I, where the ratio of the accumulated daughter ^129^Xe to stable ^127^I in the host mineral reflects the I-isotope ratio ^129^I/^127^I at closure to Xe loss. It is measured as the ratio of radiogenic ^129^Xe to ^128^Xe produced by artificial neutron irradiation. Since none of the parent nuclei remains, ^129^I-^129^Xe is by nature a relative chronometer but, when referenced to a standard of a known age, it becomes an absolute chronometer reflecting a true closure time. It is a logical technique to apply to early Solar System processes like aqueous alteration; the short half-life of ^129^I (16.14 ± 0.12 Myr) gives the I-Xe chronometer the precision needed for high temporal resolution (Hohenberg and Pravdivtseva 2008). I-Xe dating cannot be done *in situ*, and samples to be analyzed have to be separated from their host meteorites.

Samples are typically irradiated to the fluence of about $2\times 10^{19}$ thermal neutrons/cm^2^. This ensures that the ratio of I-derived radiogenic ^129^Xe to ^128^Xe is close to 1 and thus could be measured with high precision. Each irradiation has its unique ratio of ^128*^Xe (production by neutron irradiation) to ^127^I, which would be nominally established by estimation of the neutron capture probability, thus yielding the chronometrically significant ^129^I/^127^I ratio at closure of the iodine host mineral. However, direct monitoring of the neutron capture probability is impractical. An alternative calibration procedure by means of a reference meteorite standard, the Shallowater aubrite, is the usual method used in I-Xe dating. This method yields the highest precision of both relative (to the irradiation standard) and absolute closure times.

The absolute age of Shallowater has not been measured directly due to the low U content; instead, it is derived from the observed correlation between I–Xe and Pb–Pb ages in several samples. This approach allows fine tuning of the absolute age normalization when the new data becomes available. Currently, the re-evaluated absolute I–Xe age of Shallowater is 4562.4 ± 0.2 Ma (Pravdivtseva et al. 2017). The only way for chronometric data to have a meaningful interpretation is to know what mineral phase is being analyzed. Identification of iodine host phases is not easy when these components are small, and the separation of pure mineral phases is not possible. For CI chondrites, magnetite is known to be an iodine carrier, and another carrier(s) appears to exist in ferromagnetic separates (Hohenberg et al. 2000; Pravdivtseva et al. 2018).

The ^129^I-^129^Xe ages of pure magnetite chemically separated from the Orgueil (CI) chondrite have been reported by Lewis and Anders (1975) and revisited by Pravdivtseva et al. (2018). The refined I-Xe ages are consistent with each other within uncertainties and correspond to 4564.3 ± 0.3 Ma (2.9 ± 0.3 Myr after CAIs) (Pravdivtseva et al. 2018). On the other hand, the I-Xe ages of ferromagnetic separates prepared with a hand magnet from a finely-ground powder are younger than the magnetite ages by 3.4 Myr (Pravdivtseva et al. 2018) or by 4.9 Myr (Hohenberg et al. 2000). Based on these ages, it has been argued that the I-Xe ages of the ferromagnetic separates can be explained by the mixture of magnetite and a hypothetical, not yet identified second carrier phase. These ages also suggest that aqueous alteration of Orgueil started at ∼2.9 Myr after CAIs and lasted for at least 5 Ma. The former value is in reasonably good agreement with ^53^Mn-^53^Cr ages of carbonates in carbonaceous chondrites (Fig. S8).

### Remaining Questions and Future Work

See the Supplementary text.

## Supplementary Information

Below are the links to the electronic supplementary material.

Supplementary text (DOCX 37 kB)

Supplementary figures (DOCX 1.7 MB)

Table S1 (XLSX 21 kB)

Table S2 (XLSX 28 kB)

Table S3 (DOCX 39 kB)
