# Supplementary material for: Low-Temperature Aqueous Alteration of Chondrites
Source: Space Sci Rev. 2025 Feb 4;221(1):11. doi: 10.1007/s11214-024-01132-8 (PMC11794400; doi:10.1007/s11214-024-01132-8)
Supplement: Supplementary file 1 — Supplementary text (DOCX 37 kB) [file 11214_2024_1132_MOESM1_ESM.docx]

**Low-Temperature Aqueous Alteration of Chondrites, Supplementary text**

**Section 3. Mineralogy of hydrated dark clasts**

**Remaining questions and future work**

From the overview and discussions in this section, it is clear that dark clasts represent an important reservoir of materials to study the earliest evolution of our Solar System. In this regard, the following questions come to mind that should drive future research on these objects:

1. How are C1 clasts from different chondrites related to each other? Or how many unsampled parent bodies do these clasts represent?
2. Are they chemically and isotopically CI-like?
3. What does this tell us about the dust composition of the disk over time?
4. How are these clasts of importance to the prebiotic inventory of the inner Solar System?
5. When did they arrive in the inner Solar System?

**Section 4. Low-temperature alteration effects on presolar grains**

**Potential effects of low-temperature aqueous alteration on grain compositions**

Observations have revealed circumstellar silicates to be Fe-poor (<10 at. %) (e.g., Min et al. 2007), which is supported by equilibrium condensation models (e.g., Gail 2010). In contrast, many stardust silicates have relatively high Fe contents (≥10 at. %) (Bose et al. 2010; Ong and Floss 2015; Leitner et al. 2020) (Fig. S2), contradicting equilibrium condensation models predicting much lower Fe contents for silicates forming in stellar environments (e.g., Min et al. 2007; Gail 2010).

The origin of this Fe is still being studied (e.g., Floss et al. 2008; Floss and Stadermann 2009a; Vollmer et al. 2009b; Bose et al. 2010, Nguyen et al. 2010; Ong and Floss 2015; Leitner et al. 2020). Enhanced Fe contents may be explained by non-equilibrium condensation in stellar outflows (Gail and Sedlmayr 1999; Gail 2010), Fe ion implantation, preferential sputtering of Mg and Si relative to Fe in the ISM (e.g., Jones 2000), or parent body processes like fluid-mineral interactions (Le Guillou et al. 2015; Hopp and Vollmer 2018; Vollmer et al. 2020a), and diffusion (e.g., Vollmer et al. 2009b; Floss and Stadermann 2012). The dataset for presolar silicates from chondrites of petrologic type <3.0 is much smaller than for type ≥3.0. However, even for circumstellar grains from minimally altered host materials, there is a significant fraction with Mg/(Mg+Fe) <0.5 (Fig. S2). Thus, Fe appears to be introduced into the grains prior to incorporation into the asteroidal parent bodies, or already during the earliest stages of parent body alteration.

**Section 5: Evolution of organic matter during low-temperature aqueous alteration**

**Remaining questions and future work**

Several critical questions still need to be explored to understand the effects of low-temperature alteration on organic matter: (1) How does the analytical distinction between IOM and SOM affect our understanding of alteration processes and the precursors of organic compounds in meteorites? (2) Which molecules and functional groups are modified by alteration when trapped within phyllosilicates, and how does this affect the overall composition? (3) What can the alteration conditions and the relationships between various organic compounds tell us about the nature of the precursors in the early Solar System? (4) The IOM, SOM and carbonates do not account for all the C (Alexander et al. 2017). Where is the ~30–50 % of the total C that remains unaccounted for? Does it correspond to compounds weakly bound to the IOM and lost during its extraction, or in soluble material so tightly associated to phyllosilicates that its extraction is inefficient? If this reservoir is related to aqueous alteration, its molecular and isotopic characterization would offer another chance to understand the role of fluids on the organic matter evolution.

**Section 7.** **Fluid inclusions in aqueously formed minerals**

**Remaining questions and future work**

Fluid inclusions found in extraterrestrial materials have been divided into (1) those in halite clasts of H chondrites (Monahans and Zag) and (2) those in minerals from aqueously altered samples; calcite from CM (Sutter's Mill) and pyrrhotite from Ryugu samples. (1) is CO_2_-free while (2) is CO_2_-rich. Ice as the source of (1) originated from a celestial body outside of the Solar System (cometary) and is probably related to cryovolcanism in ice-bearing bodies (C-type Ceres). (2) were trapped in minerals during aqueous alteration in the parent bodies (C-type asteroids), which formed outside the CO_2_ snow line.

Water on Earth and many other bodies in the Solar System are essentially brines, in common with Mars (Rivera-Valentín et al. 2020), Ceres (Nathues et al. 2022), several satellites (e.g., Triton, Titan and Enceladus, Europa, Ganymede, and Miranda), and the Kuiper Belt object Charon (e.g., Lellouch et al. 2003; Cook et al. 2007). An exception is pure water in the perpetual shadows of the Moon and Mercury because of repeated evaporation-condensation processes (Teolis et al. 2023). Water in asteroids, where aqueous alteration occurred, is no exception to this. The associated organic matter indicates the importance of water-mineral-organics interactions.

In the future, we hope to analyze many meteorites and returned extraterrestrial samples. New analytical methods should be combined with XCT, such as cryo-TEM/EDS (Yoshida et al. 2018), electron energy loss spectroscopy (EELS), atomic force microscopy-based infrared spectroscopy (AFM-IR), and STXM. The inclusions in calcite (Tsuchiyama et al. 2021) suggest that smaller inclusions have a greater chance for aqueous fluid to survive mechanical disturbance, such as by shock. Improving the spatial resolution of the analysis is important for future research. In addition to the detection of CH_4_, NH_3_, and various cations and anions, quantitative analysis, including the CO_2_/H_2_O ratio and isotopic compositions such as O and H, will further deepen our understanding of the origin of extraterrestrial fluids and the locations of parent body formation.

**Section 9. Chronology of aqueous alteration**

**Remaining questions and future work**

Here we raise a few issues regarding the chronology of low-temperature aqueous alteration to be addressed in future work.

1. It is not clear to what event the secondary mineral formation exactly corresponds. For instance, whether the carbonate formation occurred during prograde or retrograde alteration is not obvious. For constructing thermal evolution models (e.g., Fujiya et al. 2012; Bischoff et al. 2021), most previous studies have assumed carbonate formation in the initial stages of alteration. Specifically, the time in the models when internal temperatures of water-rich planetesimals rise to 0 °C and liquid water becomes available has been adjusted to the ages of carbonates. An experimental study by Vacher et al. (2019b) suggests that carbonates are one of the first-generation secondary minerals in CM chondrites followed by tochilinite and cronstedtite. Further experimental as well as theoretical studies of aqueous alteration are needed so that the formation ages of carbonates can be used to constrain thermal evolution models of meteorite parent bodies.

2. There are still a limited number of Mn-Cr ages of carbonates obtained using proper standards. Since the work by Sugiura et al. (2010), the importance for using carbonate standards has been recognized. However, because the synthesis of dolomite grains with homogeneous Mn and Cr contents has been unsuccessful, most dolomite ages have been obtained using synthetic calcite standards (Table S3). Recently, Sugawara et al. (2022) synthesized dolomite in a laboratory via amorphous Ca- and Mg-carbonate. Amorphous carbonate is structurally flexible, and thus, can contain incompatible elements like U, Pb, and REEs (Miyajima et al. 2020). If dolomite with Mn and Cr can be synthesized via amorphous carbonate, it would provide a great improvement on the accuracy of Mn-Cr ages obtained from that mineral.

Implantation of Cr ions into natural carbonates is an ingenious approach to produce carbonate standards with Cr and was used for the Mn-Cr dating of Ryugu carbonates. However, one should take special care to keep the analytical conditions of SIMS exactly the same between standards and unknown samples. For measurements of standards, accurate depth profiles of implanted Cr up to 1 µm beneath the outer surface of the standards must be obtained (Steele et al. 2017; McCain et al. 2020). To do so, the SIMS primary ion beam is usually rastered over large (e.g., 50×50 μm^2^) areas to make pits with a shallow and flat bottom. However, due to small grain sizes, a primary ion beam was rastered over smaller areas (2×2 to 5×5 μm^2^ for Ryugu carbonates, resulting in deeper SIMS pits; McCain et al. 2023). Since it is known that RSFs are highly dependent on the analytical conditions, in particular, the depth of SIMS pits and raster size of a primary ion beam (Sugiura et al. 2010), this different approach may have resulted in a significant difference between RSFs of Ryugu and standard carbonates. In future work, measurements of Ryugu carbonates, carbonate standards with implanted Cr, and Cr-bearing synthetic dolomite under the same analytical conditions may confirm the old ages of Ryugu carbonates or revise them to younger ones.

3. The duration of aqueous activity provides crucial information about the size of meteorite parent bodies, however, it remains poorly constrained, because the number of ^129^I-^129^Xe and ^53^Mn-^53^Cr ages of aqueously formed carbonates and magnetite obtained is very limited. For example, ^129^I-^129^Xe ages of magnetite have been obtained only for one CI chondrite, Orgueil. More representative data set for several CI chondrites may reveal a range of I-Xe ages and could constrain the duration of aqueous alteration. Another approach to constrain the duration of aqueous alteration would be measuring the Mn-Cr ages of the so-called type 2 calcite. Calcite grains in CM chondrites formed either by precipitation from a fluid or by replacement of other minerals. The former is referred to as type 0 or 1 calcite and typically has blocky shapes, small grain sizes and few inclusions. On the other hand, the latter is type 2 calcite, which is larger and contains sulfide inclusions (Tyra et al. 2012; Lee et al. 2013; Vacher et al. 2017, 2018). It is inferred from their oxygen isotopic compositions that type 2 grains formed from more isotopically evolved fluids resulting from O-isotope exchange between ^16^O-rich anhydrous rock and ^16^O-poor water. Therefore, type 2 calcite likely postdates type 0 and 1 calcite. The Mn-Cr ages of calcite have been measured only for type 1 grains, and thus, the Mn-Cr dating of type 2 grains might provide younger ages than type 1 grains and put a lower limit on the duration of aqueous alteration in the CM chondrite parent body.

**References**

Alexander CMO’D, Cody GD, De Gregorio BT et al (2017) The nature, origin and modification of insoluble organic matter in chondrites, the major source of Earth’s C and N. Geochemistry 77:227–256.

Bischoff A, Alexander CMO’D, Barrat J-A et al (2021) The old, unique C1 chondrite Flensburg – Insight into the first processes of aqueous alteration, brecciation, and the diversity of water-bearing parent bodies and lithologies. Geochim Cosmochim Acta 293:142–186.

Bose M, Floss C, Stadermann FJ (2010) An investigation into the origin of Fe-rich presolar silicates in Acfer 094. Astrophys. J. 714:1624–1636.

Cook JC, Desch SJ, Roush TL et al (2007) Near-infrared spectroscopy of Charon: possible evidence for cryovolcanism on Kuiper belt objects. Astrophys J 663:1406.

Floss C, Stadermann FJ (2009a) Auger Nanoprobe analysis of presolar ferromagnesian silicate grains from primitive CR chondrites QUE 99177 and MET 00426. Geochim Cosmochim Acta 73:2415–2440.

Floss C, Stadermann FJ (2009b) High abundances of circumstellar and interstellar C-anomalous phases in the primitive CR3 chondrites QUE 99177 and MET 00426. Astrophys J 697:1242–1255.

Floss C, Stadermann FJ (2012) Presolar silicate and oxide abundances and compositions in the ungrouped carbonaceous chondrite Adelaide and the K chondrite Kakangari: The effects of secondary processing. Meteorit Planet Sci 47:992–1009.

Floss C, Stadermann FJ, Bose M (2008) Circumstellar Fe oxide from the Acfer 094 carbonaceous chondrite. Astrophys J 672:1266–1271.

Fujiya W, Sugiura N, Hotta H et al (2012) Evidence for the late formation of hydrous asteroids from young meteoritic carbonates. Nature Comms 3:627.

Gail H-P (2010) Formation and evolution of minerals in accretion disks and stellar outflows. Lect Notes Phys 815:61–141.

Gail H-P, Sedlmayr E (1999) Mineral formation in stellar winds. I. Condensation sequence of silicate and iron grains in stationary oxygen rich outflows. Astron Astrophys 347:594–616

Hopp T, Vollmer C (2018) Chemical composition and iron oxidation state of amorphous matrix silicates in the carbonaceous chondrite Acfer 094. Meteorit Planet Sci 53:153–166.

Jones AP (2000) Depletion patterns and dust evolution in the interstellar medium. J Geophys Res 105:10257–10268.

Le Guillou C, Changela HG, Brearley AJ (2015) Widespread oxidized and hydrated amorphous silicates in CR chondrites matrices: Implications for alteration conditions and H2 degassing of asteroids. Earth Planet Sci Lett 420:162–173.

Lee MR, Sofe MR, Lindgren P et al (2013) The oxygen isotope evolution of parent body aqueous solutions as recorded by multiple carbonate generations in the Lonewolf Nunataks 94101 CM2 carbonaceous chondrite. Geochim Cosmochim Acta 121:452–466.

Leitner J, Metzler K, Vollmer C et al (2020) The presolar grain inventory of fine-grained chondrule rims in the Mighei-type (CM) chondrites. Meteorit Planet Sci 55:1176–1206.

Lellouch E, Paubert G, Moses JI et al (2003) Volcanically emitted sodium chloride as a source for Io's neutral clouds and plasma torus. Nature 421:45–47.

McCain KA, Liu M-C, McKeegan KD (2020) Calibration of matrix-dependent biases in isotope and trace element analyses of carbonate minerals. J Vac Sci Technol B 38:044005.

McCain, K.A., Matsuda, N., Liu, M.C et al (2023) Early fluid activity on Ryugu inferred by isotopic analyses of carbonates and magnetite. Nature Astro 7:309–317.

Min M, Waters LBFM, de Koter A et al (2007) The shape and composition of interstellar silicate grains. Astron Astrophys 462:667–676.

Miyajima Y, Saito A, Kagi H et al (2020) Incorporation of U, Pb and rare earth elements in calcite through crystallisation from amorphous calcium carbonate: Simple preparation of reference materials for microanalysis. Geostand Geoanalytical Res 45:189–205.

Nathues A, Hoffmann M, Schmedemann N et al (2022) Brine residues and organics in the Urvara basin on Ceres. Nat Comms 13:927.

Nguyen AN, Nittler LR, Stadermann FJ et al (2010) Coordinated analyses of presolar grains in the Allan Hills 77307 and Queen Elizabeth Range 99177 meteorites. Astrophys J 719:166–189.

Ong WJ, Floss C (2015) Iron isotopic measurements in presolar silicate and oxide grains from the Acfer 094 ungrouped carbonaceous chondrite. Meteorit Planet Sci 50:1392–1407.

Rivera-Valentín, E.G., Chevrier, V.F., Soto, A. et al. (2020) Distribution and habitability of (meta)stable brines on present-day Mars. Nat Astron 4:756–761.

Steele RCJ, Heber VS, McKeegan KD (2017) Matrix effects on the relative sensitivity factors for manganese and chromium during ion microprobe analysis of carbonate: Implications for early Solar System chronology. Geochim Cosmochim Acta 201:245–259.

Sugawara S, Fujiya W, Kagi H et al (2022) Heat-induced dolomitization of amorphous calcium magnesium carbonate in a CO_2_‑filled closed system. ACS Omega 7:44670–44676.

Sugiura N, Ichimura K, Fujiya W et al (2010) Mn/Cr relative sensitivity factors for synthetic calcium carbonate measured with a NanoSIMS ion microprobe. Geochem J 44:e11-e16.

Teolis B, Sarantos M, Schorghofer N et al (2023) Surface exospheric interactions. Space Sci Rev 219:4.

Tsuchiyama A, Miyake A, Okuzumi S et al (2021) Discovery of primitive CO_2_-bearing fluid in an aqueously altered carbonaceous chondrite. Sci Adv 7:eabg9707.

Tyra MA, Farquhar J, Guan Y et al (2012) An oxygen isotope dichotomy in CM2 chondritic carbonates – a SIMS approach. Geochim Cosmochim Acta 77:383–395.

Vacher LG, Truche L, Faure, F et al (2019b) Deciphering the conditions of tochilinite and cronstedtite formation in CM chondrites from low temperature hydrothermal experiments. Meteorit Planet Sci 54:1870–1889.

Vacher LG, Marrocchi Y, Villeneuve J et al (2017) Petrographic and C & O isotopic characteristics of the earliest stages of aqueous alteration of CM chondrites. Geochim Cosmochim Acta 213:271–290.

Vacher L, Marrocchi Y, Villeneuve J et al (2018) Collisional and alteration history of the CM parent body. Geochim Cosmochim Acta 239:213–34.

Vollmer C, Hoppe P, Stadermann FJ et al (2009a) NanoSIMS analysis and Auger electron spectroscopy of silicate and oxide stardust from the carbonaceous chondrite Acfer 094. Geochim Cosmochim Acta 73:7127–7149.

Vollmer C, Brenker FE, Hoppe P et al (2009b) Direct laboratory analysis of silicate stardust from red giant stars. Astrophys J 700:774–782.

Vollmer C, Pelka M, Leitner J et al (2020a) Amorphous silicates as a record of solar nebular and parent body processes—A transmission electron microscope study of fine-grained rims and matrix in three Antarctic CR chondrites. Meteorit Planet Sci 55:1491–1508.

Yoshida K, Orozbaev R, Hirajima T et al (2018) Micro-excavation and direct chemical analysis of individual fluid inclusion by cryo-FIB-SEM-EDS: Application to the UHP talc-garnet-chloritoid schist from the Makbal Metamorphic Complex, Kyrgyz Tian-Shan. Geochem J 52:59–67.
