# Supplementary material for: Low-Temperature Aqueous Alteration of Chondrites
Source: Space Sci Rev. 2025 Feb 4;221(1):11. doi: 10.1007/s11214-024-01132-8 (PMC11794400; doi:10.1007/s11214-024-01132-8)
Supplement: Supplementary file 2 — Supplementary figures (DOCX 1.7 MB) [file 11214_2024_1132_MOESM2_ESM.docx]

**Low-Temperature Aqueous Alteration of Chondrites, Supplementary figures**

**Fig S1** Matrix-normalized presolar O-rich grain abundances and SiC abundances for chondritic meteorites and material from asteroid Ryugu in parts per million (ppm). All errors are 1 sigma. Data sources: Marhas et al. (2006); Vollmer et al. (2009a); Zhao et al. (2011, 2013. 2014); Floss and Stadermann (2009a, 2009b, 2012); Nguyen et al. (2010, 2023); Leitner et al. (2012, 2016, 2018, 2020); Heck et al. (2013); Davidson et al. (2014a, 2014b 2015); Floss and Haenecour (2016a, 2016b); Haenecour et al. (2018); Nittler et al. (2018, 2020, 2021); Verdier-Paoletti et al. (2020); Barosch et al. (2022a, 2022b); Smith et al. (2023)

**Fig S2** Mg-Fe-Si element compositions (in atomic %) obtained by auger electron spectroscopy for presolar silicates from the most pristine carbonaceous chondrites (sky blue circles; Acfer 094, ALHA 77307, DOM 08006, LAP 031117) (Vollmer et al. 2009a; Bose et al. 2010, 2012; Nguyen et al. 2010; Haenecour et al. 2018), minimally altered CR chondrites (deep blue circles; MET 00426, QUE 99177, GRV 021710) (Floss and Stadermann 2009a; Nguyen et al. 2010; Zhao et al. 2013), together with data for silicates from more altered CR and CM chondrites (red circles; EET 92161, GRA 95229, Jbilet Winselwan, Murchison, Murray) (Leitner et al. 2016, 2020), and the C-ungrouped chondrite Adelaide (black diamonds) (Floss and Stadermann 2012). Dotted vertical lines denote the compositions of stoichiometric pyroxene ([Mg+Fe]/Si = 1) and olivine ([Mg+Fe]/Si = 2). The horizontal line marks the border between Mg-rich and Fe-rich compositions


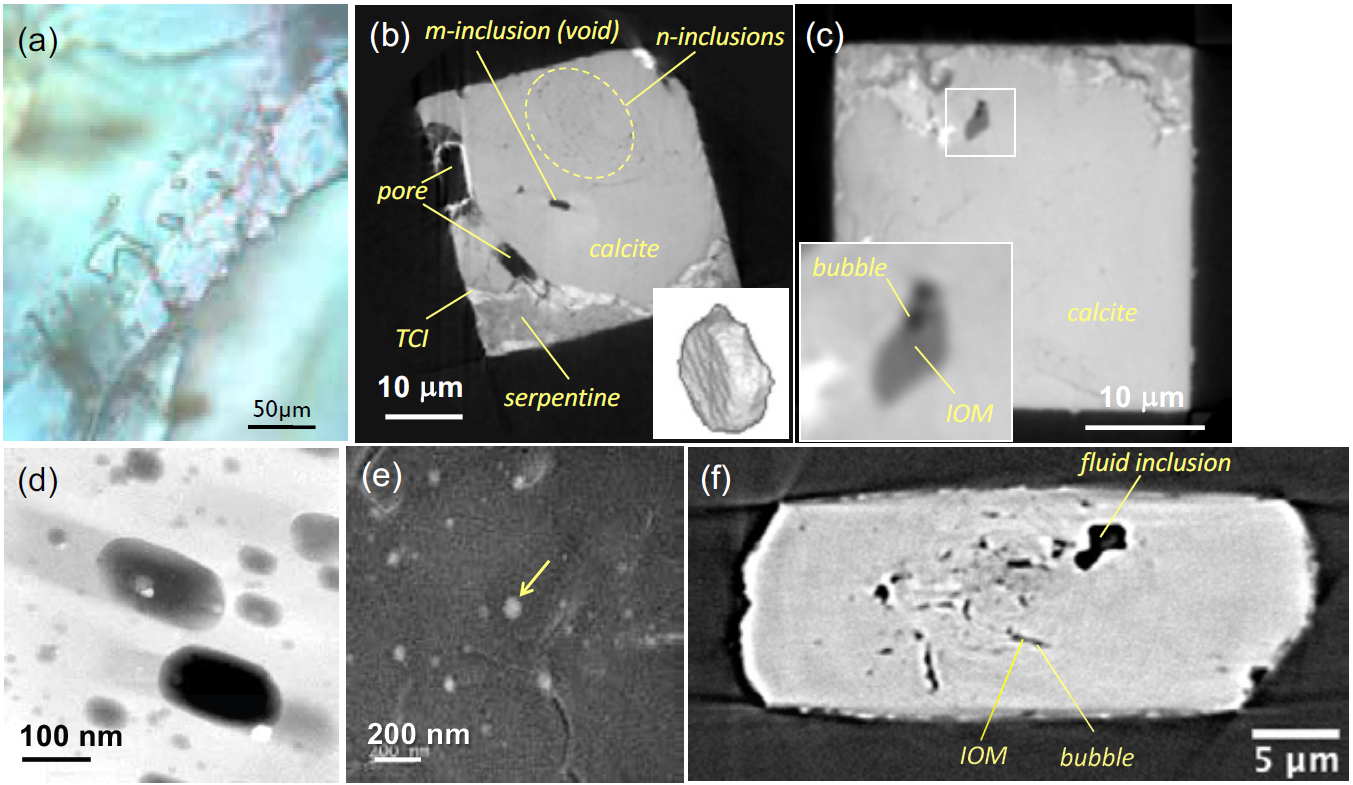


**Fig S3** Aqueous fluid and related inclusions in minerals from different extraterrestrial materials. (a) Aqueous fluid inclusions in Monahans halite. (b) Phase-shift CT image (8 keV) of a micrometer-sized inclusion (m-inclusion) in Sutter’s Mill calcite together with matrix (Tsuchiyama et al. 2021). The micrometer-sized inclusion is empty and has a hexagonal plate shape (inlet). Two large pores are connected to the matrix or the outside of the sample in 3D. The region where a large number of nanometer-sized inclusions (n-inclusions) are distributed is indicated by the dotted circle. TCI: tochilinite-cronstedtite inclusion. (c) Phase-shift CT image (8 keV) of an IOM inclusion in Sutter’s Mill calcite together with matrix. The IOM inclusion contains two bubbles (inlet). (d) STEM image of nanometer-sized inclusions in Sutter’s Mill calcite. The inclusions have facets, and some contain tiny faceted objects as trapped or daughter crystals. (e) TEM bright-field image of an aqueous fluid inclusion (indicated by an arrow) in Sutter’s Mill calcite. Modified from Tsuchiyama et al. (2021). (f) Absorption CT image (7 keV) showing a fluid inclusion in Ryugu pyrrhotite. IOM inclusions with bubbles connected to the exterior are also observed.

**Fig S4** Estimates for the oxygen-isotopic composition of the CM water based on different methods defining a line with a slope of 0.669 ± 0.029 and intercept of -1.92 ± 0.80 (details in text; Clayton and Mayeda 1984, 1999; Baker et al. 2002; Guo and Eiler 2007; Fujiya 2018; Alexander 2019). PCM = primitive chondrule mineral line.

**Fig S5** A) Positive trends observed between bulk δD and C/H ratios obtained for CM and CR chondrites and interpreted as two component mixtures of D-rich organic matter and D-poor/C-free water ices. For CM chondrites, a comparison between data obtained with (Vacher et al. 2020) and without (Alexander et al. 2012) pre-degassing of the samples (48 h, 120 °C under vacuum) prior to analyzing their H-isotope compositions is presented. The linear regressions obtained for these two sets of data have distinct slopes but similar intercepts, within error. The zero intercepts give estimates of the H-isotope compositions of the water ice accreted on the CM and CR parent bodies (Alexander et al., 2012). Results obtained using stepwise pyrolysis for indigenous CM chondrite water (after removal of the weakly-bound water) are also plotted for comparison (Lee et al. 2023). B) The average D/H ratio of IOM in CM chondrites is consistent with the extrapolation of the regression based on the pre-degassed measurements, but inconsistent with those performed without pre-degassing (IOM data from Alexander et al. 2010). Figure adapted from Alexander et al. (2012) and Marrocchi et al. (2023b). Considering the anomalous nature of the C2-ungrouped chondrites Bells and Essebi (e.g., Hewins et al. 2021; Marrocchi et al. 2022), these chondrites were not included in the CM bulk and IOM dataset. These can lead to differences with data reported in Alexander et al. (2010, 2012).

**Fig S6** C and O isotope *in situ* analyses obtained on carbonates in CM chondrites (Fujiya et al. 2015; Tyra et al. 2016; Vacher et al. 2017; Telus et al. 2019) and in the CI chondrite Ivuna and in Ryugu’s samples (Fujiya et al. 2023) showing the absence of a unique trend between δ^13^C and δ^18^O. Data in Table S1

**Fig S7** δ^13^C values of Ca-carbonates measured in CM chondrites (Grady et al. 1988; Fujiya et al. 2015; Alexander et al. 2015; Tyra et al. 2016; Vacher et al. 2017, 2018; Telus et al. 2019), CI and Ryugu’s samples (Fujiya et al. 2023) and in the C-ungrouped Tagish Lake (Fujiya et al. 2019) compared to cometary δ^13^C values (in CN averaged over 19 comets and bulk δ^13^C estimated for the 67P/Churyumov-Gerasimenko (67P/C-G) comet visited by the Rosetta mission; Müller et al. 2022 and references therein), δ^13^C of bulk compositions of chondrites (see compilation), chondritic insoluble and soluble organic matter (IOM and SOM) values (data from Alexander et al. 2007, 2010; Naraoka et al. 2023; Pizzarello et al. 2017 and reference therein).


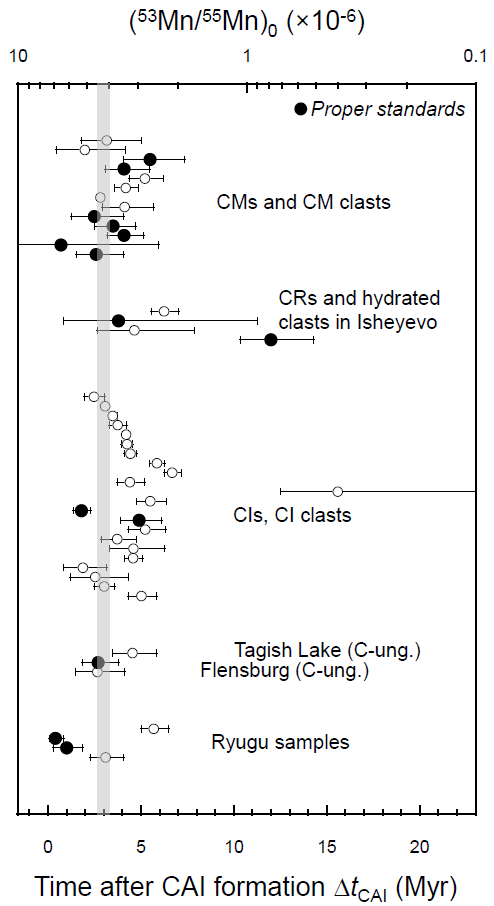


**Fig. S8** The initial ^53^Mn/^55^Mn ratios [(^53^Mn/^55^Mn)_0_] and ages of carbonates in CR, CM, CI, and ungrouped carbonaceous chondrites, hydrated clasts in the Isheyevo CH/CB-like meteorite, and Ryugu samples relative to CV CAIs (Δ*t*_CAI_). Data obtained using proper (matrix-matched) standards are shown by filled symbols. The gray band represents the ^129^I-^129^Xe age of magnetite from Orgueil (Pravdivtseva et al. 2018).

**References**

Alexander CMO’D, Fogel M, Yabuta H, Cody G (2007) The origin and evolution of chondrites recorded in the elemental and isotopic compositions of their macromolecular organic matter. Geochim Cosmochim Acta 71:4380–4403.

Alexander CMO’D, Newsome SD, Fogel ML et al (2010) Deuterium enrichments in chondritic macromolecular material—Implications for the origin and evolution of organics, water and asteroids. Geochim Cosmochim Acta 74:4417–4437.

Alexander CMO’D, Bowden R, Fogel M et al (2012) The provenances of asteroids, and their contributions to the volatile inventories of the terrestrial planets. Science 337:721–723.

Alexander CMO’D, Bowden R, Fogel ML et al (2015) Carbonate abundances and isotopic compositions in chondrites. Meteorit Planet Sci 50:810–833.

Alexander CMO’D (2019) Quantitative models for the elemental and isotopic fractionations in chondrites: The carbonaceous chondrites. Geochim Cosmochim Acta, 254:277–309.

Baker L, Franchi IA, Wright IP et al (2002) The oxygen isotopic composition of water from Tagish Lake: Its relationship to low-temperature phases and to other carbonaceous chondrites. Meteorit Planet Sci 37:977–985.

Barosch J, Nittler LR, Wang J et al (2022a) Presolar O- and C-anomalous grains in unequilibrated ordinary chondrite matrices. Geochim Cosmochim Acta 335:169–182.

Barosch J, Nittler LR, Wang J et al (2022b) Presolar stardust in asteroid Ryugu. Astrophys J Lett 935:L3–L14.

Bose M, Floss C, Stadermann FJ (2010) An investigation into the origin of Fe-rich presolar silicates in Acfer 094. Astrophys. J. 714:1624–1636.

Bose M, Floss C, Stadermann FJ et al (2012) Circumstellar and interstellar material in the CO3 chondrite ALHA77307: an isotopic and elemental investigation. Geochim Cosmochim Acta 93:77–101.

Clayton RN and Mayeda TK (1984) The oxygen isotope record in Murchison and other carbonaceous chondrites. Earth Planet Sci Lett 67:151–166.

Clayton RN, Mayeda TK (1999) Oxygen isotope studies of carbonaceous chondrites. Geochim Cosmochim Acta 63:2089–2104.

Davidson J, Busemann H, Nittler LR, et al (2014a) Abundances of presolar silicon carbide grains in primitive meteorites determined by NanoSIMS. Geochim Cosmochim Acta 139:248–266.

Davidson J, Nittler LR, Alexander CMO’D et al (2014b) Presolar materials and nitrogen isotope anomalies in the unique carbonaceous chondrite Miller Range 07687. Lunar Planet Sci 45:abstract #1603

Davidson J, Alexander CMO’D, Schrader DL et al (2015) Miller Range 090657: a very pristine Renazzo-like (CR) Carbonaceous Chondrite. Lunar Planet Sci 46:abstract #1376

Floss C, Haenecour P (2016a) Presolar silicate abundances in the unequilibrated ordinary chondrites Meteorite Hills 00526 and Queen Alexandra Range 97008. Meteorit Planet Sci 51:A257.

Floss C, Haenecour P (2016b) Meteorite Hills (MET) 00526: An unequilibrated ordinary chondrite with high presolar grain abundances. Lunar Planet Sci 47:abstract #1030

Floss C, Stadermann FJ (2009a) Auger Nanoprobe analysis of presolar ferromagnesian silicate grains from primitive CR chondrites QUE 99177 and MET 00426. Geochim Cosmochim Acta 73:2415–2440.

Floss C, Stadermann FJ (2009b) High abundances of circumstellar and interstellar C-anomalous phases in the primitive CR3 chondrites QUE 99177 and MET 00426. Astrophys J 697:1242–1255.

Floss C, Stadermann FJ (2012) Presolar silicate and oxide abundances and compositions in the ungrouped carbonaceous chondrite Adelaide and the K chondrite Kakangari: The effects of secondary processing. Meteorit Planet Sci 47:992–1009.

Fujiya W (2018) Oxygen isotopic ratios of primordial water in carbonaceous chondrites. Earth Planet Sci Lett 481:264–272.

Fujiya W, Sugiura N, Marrocchi Y et al (2015) Comprehensive study of carbon and oxygen isotopic compositions, trace element abundances, and cathodoluminescence intensities of calcite in the Murchison CM chondrite. Geochim Cosmochim Acta 161:101–117.

Fujiya W, Hoppe P, Ushikubo T et al (2019) Migration of D-type asteroids from the outer Solar System inferred from carbonate in meteorites. Nat Astron 3:910–915.

Fujiya W, Kawasaki N, Nagashima K et al. (2023) Carbonate record of temporal change in oxygen fugacity and gaseous species in asteroid Ryugu. Nat Geosci 16:675–682.

Grady MM, Wright IP, Carr RH (1988) Differences in isotopic composition of carbonaceous components in enstatite chondrites. Earth Planet Sci Lett 87:293–302.

Guo W, Eiler JM (2007) Temperatures of aqueous alteration and evidence for methane generation on the parent bodies of the CM chondrites. Geochim Cosmochim Acta 71:5565–5575.

Haenecour P, Floss C, Zega TJ et al (2018) Presolar silicates in the matrix and fine-grained rims around chondrules in primitive CO3.0 chondrites: Evidence for pre-accretionary aqueous alteration of the rims in the solar nebula. Geochim Cosmochim Acta 221:379–405.

Heck PR, Floss C, Davis AM (2013) Stardust in the Sutter’s Mill meteorite. Meteorit Planet Sci 48:A158.

Hewins RH et al. (2021) Northwest Africa (NWA) 12563 and ungrouped C2 chondrites: Alteration styles and relationships to asteroids. Geochimica et Cosmochimica Acta 311, 238-273,

Lee, M.R., Hallis, L.J., Daly, L. and Boyce, A.J. (2023), The water content of CM carbonaceous chondrite falls and finds, and their susceptibility to terrestrial contamination. Meteorit Planet Sci, 58: 1760-1772.

Leitner J, Vollmer C, Hoppe P et al (2012) Characterization of presolar material in the CR Chondrite Northwest Africa 852. Astrophys J 745:38–53.

Leitner J, Vollmer C, Floss C et al (2016) Ancient stardust in fine-grained chondrule dust rims from carbonaceous chondrites. Earth Planet Sci Lett 434:117–128.

Leitner J, Hoppe P, Zipfel J (2018) A study of presolar material in hydrated lithic clasts from metal-rich carbonaceous chondrites. Meteorit Planet Sci 53:204–231.

Leitner J, Metzler K, Vollmer C et al (2020) The presolar grain inventory of fine-grained chondrule rims in the Mighei-type (CM) chondrites. Meteorit Planet Sci 55:1176–1206.

Marhas KK, Hoppe P, Stadermann FJ et al (2006) The distribution of presolar grains in CI and CO meteorites. Lunar Planet Sci 37:abstract #1959

Marrocchi Y, Rigaudier T, Piralla M et al (2023b) Hydrogen isotopic evidence for nebular pre-hydration and the limited role of parent-body processes in CM chondrites. Earth Planet Sci Lett 611:118151.

Marrocchi Y, Piralla M, Regnault M et al (2022) Isotopic evidence for two chondrule generations in CR chondrites and their relationships to other carbonaceous chondrites. Earth Planet Sci Lett 593:117683.

Müller DR, Altwegg K, Berthelier JJ et al. (2022) High D/H ratios in water and alkanes in comet 67P/Churyumov-Gerasimenko measured with Rosetta/ROSINA DFMS. Astron Astrophys 662:1–13.

Naraoka, H., Takano, Y., Dworkin, J.P et al (2023) Soluble organic molecules in samples of the carbonaceous asteroid (162173) Ryugu. Science (1979) 379.

Nguyen AN, Nittler LR, Stadermann FJ et al (2010) Coordinated analyses of presolar grains in the Allan Hills 77307 and Queen Elizabeth Range 99177 meteorites. Astrophys J 719:166–189.

Nguyen N et al (2023) Abundant presolar grains and primordial organics preserved in carbon-rich exogenous clasts in asteroid Ryugu. Sci Adv 9:eadh1003.

Nittler LR, Alexander CMO’D, Davidson J et al (2018) High abundances of presolar grains and 15N-rich organic matter in CO3.0 chondrite Dominion Range 08006. Geochim Cosmochim Acta 226:107–131.

Nittler LR, Stroud RM, Alexander CMO’D et al (2020) Presolar grains in primitive ungrouped carbonaceous chondrite Northwest Africa 5958. Meteorit Planet Sci 55:1160–1175.

Nittler LR, Alexander CMO’D, Patzer A et al (2021) Presolar stardust in highly pristine CM chondrites Asuka 12169 and Asuka 12236. Meteorit Planet Sci 56:260–276.

Pizzarello S, Shock E (2017) Carbonaceous Chondrite Meteorites: the Chronicle of a Potential Evolutionary Path between Stars and Life. Origins of Life and Evolution of Biospheres 47, 249–260.

Pravdivtseva O, Krot AN, Hohenberg CM (2018) I-Xe dating of aqueous alteration in the CI chondrite Orgueil: I. Magnetite and ferromagnetic separates. Geochim Cosmochim Acta 227:38–47.

Smith LR, Haenecour P, Barnes J et al (2023) Abundance of presolar grains in the C3.00-ungrouped chondrite Chwichiya 002. Lunar Planet Sci 54:abstract #2425

Telus M, Alexander CMO’D, Hauri EH et al (2019) Calcite and dolomite formation in the CM parent body: Insight from in situ C and O isotope analyses. Geochim. Cosmochim. Acta 260:275–291.

Tyra M, Brearley A, Guan Y (2016) Episodic carbonate precipitation in the CM chondrite ALH 84049: An ion microprobe analysis of O and C isotopes. Geochim Cosmochim Acta 175, 195–207.

Vacher LG, Marrocchi Y, Villeneuve J et al (2017) Petrographic and C & O isotopic characteristics of the earliest stages of aqueous alteration of CM chondrites. Geochim Cosmochim Acta 213:271–290.

Vacher L, Marrocchi Y, Villeneuve J et al (2018) Collisional and alteration history of the CM parent body. Geochim Cosmochim Acta 239: 213–34.

Vacher LG, Piani L, Rigaudier Y et al (2020) Hydrogen in chondrites: Influence of parent body alteration and atmospheric contamination on primordial components. Geochim Cosmochim Acta 281:53–66.

Verdier-Paoletti MJ, Nittler LR, Wang J (2020) New estimation of presolar grain abundances in the Paris meteorite. Lunar Planet Sci 51:abstract #2523

Vollmer C, Hoppe P, Stadermann FJ et al (2009a) NanoSIMS analysis and Auger electron spectroscopy of silicate and oxide stardust from the carbonaceous chondrite Acfer 094. Geochim Cosmochim Acta 73:7127–7149.

Zhao X, Floss C, Stadermann FJ et al (2011) Continued investigation of presolar silicate grains in the carbonaceous chondrite Ningqiang. Lunar Planet Sci 42:abstract #1982

Zhao X, Floss C, Lin Y et al (2013) Stardust investigation into the CR chondrite Grove Mountain 021710. Astrophys J 769:49–64.

Zhao X, Lin Y, Yin Q-Z et al (2014) Presolar grains in the CM2 chondrite Sutter’s Mill. Meteorit Planet Sci 49:2038–2046.
