# Supplementary material for: Low-Temperature Aqueous Alteration of Chondrites
Source: Space Sci Rev. 2025 Feb 4;221(1):11. doi: 10.1007/s11214-024-01132-8 (PMC11794400; doi:10.1007/s11214-024-01132-8)
Supplement: Supplementary file 5 — Table S3 (DOCX 39 kB) [file 11214_2024_1132_MOESM5_ESM.docx]

**Low-Temperature Aqueous Alteration of Chondrites, Section 9 Chronology, Table S3**

Initial ^53^Mn/^55^Mn ratios and absolute ages of carbonate minerals in CR, CM, CI, and ungrouped carbonaceous chondrites and Ryugu samples.

| Sample | Meteorite group | Mineral | (^53^Mn/^55^Mn)_0_ (×10^-6^) | 2σ | Absolute age (Ma) | er. + | er. - | Δ*t*_CAI_ (Myr) | Standard | Ref |
| --- | --- | --- | --- | --- | --- | --- | --- | --- | --- | --- |
| QUE 93005 | CM | dolomite (dominant), calcite | 4.1 | 1.2 | 4564.2 | 1.4 | 1.9 | 3.1 | SC olivine | [1] |
| ALH 83100 | CM | dolomite | 5.1 | 1.7 | 4565.3 | 1.5 | 2.2 | 2.0 | SC olivine | [1] |
| Murchison | CM | calcite | 2.66 | 0.79 | 4561.8 | 1.4 | 1.9 | 5.5 | Syn calcite | [2] |
| Y-791198 | CM | calcite | 3.40 | 0.74 | 4563.2 | 1.1 | 1.3 | 4.1 | Syn calcite | [2] |
| ALH 83100 | CM | dolomite | 2.79 | 0.47 | 4562.1 | 0.8 | 1.0 | 5.2 | Syn calcite | [2] |
| Sayama | CM | dolomite | 3.38 | 0.40 | 4563.1 | 0.6 | 0.7 | 4.2 | Syn calcite | [2] |
| QUE 93005 | CM | dolomite | 4.37 | 0.19 | 4564.5 | 0.2 | 0.2 | 2.8 | Syn diopside | [3] |
| Sutter's Mill | CM | dolomite | 3.42 | 0.86 | 4563.2 | 1.2 | 1.5 | 4.1 | Syn calcite | [4] |
| LON 94101* | CM | calcite | 4.80 | 6.60 | 4565.0 | 4.6 | - | 2.3 | Syn calcite | [5] |
| Kaidun1 | CM clast in C ung. | calcite | 4.66 | 1.20 | 4564.8 | 1.2 | 1.6 | 2.5 | Syn calcite | [5] |
| Kaidun1_apr | CM clast in C ung. | calcite | 3.84 | 0.78 | 4563.8 | 1.0 | 1.2 | 3.5 | Syn calcite | [5] |
| Kaidun1 combo | CM clast in C ung. | calcite | 3.44 | 0.62 | 4563.2 | 0.9 | 1.1 | 4.1 | Syn calcite | [5] |
| NWA 7542A* | CM clast in eucrite | calcite | 2.02 | 6.40 | 4560.4 | 7.6 | - | 6.9 | Syn calcite | [5] |
| PRA 04401 | CM clast in howardite | calcite | 6.53 | 4.10 | 4566.6 | 2.6 | 5.3 | 0.7 | Syn calcite | [5] |
| PRA 04402* | CM clast in howardite | calcite | 1.40 | 6.70 | 4558.4 | 9.4 | - | 8.9 | Syn calcite | [5] |
| Jbilet Winselwan | CM heated | calcite | 4.51 | 1.06 | 4564.7 | 1.1 | 1.4 | 2.6 | Syn calcite | [6] |
|  |  |  |  |  |  |  |  |  |  |  |
| Isheyevo | hydrated clast in CH/CB | dolomite | 2.30 | 0.31 | 4561.1 | 0.7 | 0.8 | 6.2 | Syn calcite | [7] |
| Renazzo N1127 | CR | calcite | 3.6 | 2.7 | 4563.5 | 3.0 | 7.4 | 3.8 | Syn calcite | [8] |
| Renazzo N1126 | CR | dolomite | 3.1 | 1.4 | 4562.7 | 2.0 | 3.2 | 4.6 | Syn calcite | [8] |
| GRO 95577 | CR | calcite | 0.79 | 0.28 | 4555.3 | 1.6 | 2.3 | 12.0 | Syn calcite | [8] |
| Orgueil, Ivuna* | CI | dolomite | 1.99 | 0.16 | 4560.3 | 0.4 | 0.4 | 7.0 | Unknown | [9] |
| Orgueil A-9 | CI | dolomite | 4.66 | 0.47 | 4564.8 | 0.5 | 0.6 | 2.5 | SC olivine and syn glass(es) | [10] |
| Orgueil B-13 | CI | dolomite | 4.16 | 0.14 | 4564.2 | 0.2 | 0.2 | 3.1 | SC olivine and syn glass(es) | [10] |
| Orgueil A-4 | CI | dolomite | 3.85 | 0.17 | 4563.8 | 0.2 | 0.2 | 3.5 | SC olivine and syn glass(es) | [10] |
| Orgueil B-8 | CI | dolomite | 3.67 | 0.32 | 4563.6 | 0.4 | 0.5 | 3.7 | SC olivine and syn glass(es) | [10] |
| Orgueil A-9 | CI | breunnerite | 3.37 | 0.06 | 4563.1 | 0.1 | 0.1 | 4.2 | SC olivine and syn glass(es) | [10] |
| Orgueil B-13 | CI | breunnerite | 3.34 | 0.19 | 4563.1 | 0.3 | 0.3 | 4.2 | SC olivine and syn glass(es) | [10] |
| Orgueil OC-3-4 | CI | breunnerite | 3.23 | 0.20 | 4562.9 | 0.3 | 0.3 | 4.4 | SC olivine and syn glass(es) | [10] |
| Orgueil OC-1 | CI | breunnerite | 2.47 | 0.18 | 4561.4 | 0.4 | 0.4 | 5.9 | SC olivine and syn glass(es) | [10] |
| Orgueil B-19 | CI | breunnerite | 2.12 | 0.18 | 4560.6 | 0.4 | 0.5 | 6.7 | SC olivine and syn glass(es) | [10] |
| Orgueil | CI | dolomite | 3.24 | 0.44 | 4562.9 | 0.7 | 0.8 | 4.4 | Syn calcite | [11] |
| Orgueil young grain | CI | dolomite | 0.40 | 0.31 | 4551.7 | 3.1 | 8.0 | 15.6 | Syn calcite | [11] |
| Ivuna | CI | dolomite | 2.64 | 0.39 | 4561.8 | 0.7 | 0.9 | 5.5 | Syn calcite | [11] |
| Orgueil Breun-25 | CI | breunnerite | 5.28 | 0.47 | 4565.5 | 0.5 | 0.5 | 1.8 | Carbs with imp-Cr | [12] |
| Orgueil Breun-33 | CI | breunnerite | 2.96 | 0.60 | 4562.4 | 1.0 | 1.2 | 4.9 | Carbs with imp-Cr | [12] |
| Ivuna | CI | dolomite | 2.78 | 0.51 | 4562.1 | 0.9 | 1.1 | 5.2 | Syn calcite | [5] |
| Alais | CI | dolomite | 3.68 | 0.64 | 4563.6 | 0.9 | 1.0 | 3.7 | Syn calcite | [5] |
| Orgueil | CI | dolomite | 3.13 | 0.84 | 4562.7 | 1.3 | 1.7 | 4.6 | Syn calcite | [5] |
| Ivuna | CI | dolomite | 3.14 | 0.28 | 4562.7 | 0.5 | 0.5 | 4.6 | Syn calcite | [13] |
| Kaidun cavity-5 | CI clast in C ung. | dolomite | 5.2 | 1.1 | 4565.4 | 1.0 | 1.3 | 1.9 | SC olivine and syn glass(es) | [14] |
| Kaidun 3.10.i-2 | CI clast in C ung. | dolomite | 4.6 | 1.3 | 4564.8 | 1.3 | 1.8 | 2.5 | SC olivine and syn glass(es) | [14] |
| Kaidun 3.10.i-3 | CI clast in C ung. | dolomite | 4.20 | 0.43 | 4564.3 | 0.5 | 0.6 | 3.0 | SC olivine and syn glass(es) | [14] |
| Kaidun (2) | C1 clast in C ung. | dolomite | 2.89 | 0.41 | 4562.3 | 0.7 | 0.8 | 5.0 | Syn calcite | [5] |
| Tagish Lake | C ung. | dolomite | 3.16 | 0.69 | 4562.8 | 1.1 | 1.3 | 4.5 | Syn calcite | [11] |
| Flensburg | C ung. | calcite | 4.43 | 0.80 | 4564.6 | 0.9 | 1.1 | 2.7 | Syn calcite | [15] |
| Flensburg | C ung. | dolomite | 4.50 | 1.08 | 4564.6 | 1.2 | 1.5 | 2.7 | Syn calcite | [15] |
|  |  |  |  |  |  |  |  |  |  |  |
| Ryugu A0058 | CI-like | dolomite | 2.55 | 0.35 | 4561.6 | 0.7 | 0.8 | 5.7 | Syn calcite | [13] |
| Ryugu A0037 | CI-like | dolomite | 6.80 | 0.50 | 4566.9 | 0.4 | 0.4 | 0.4 | Carbs with imp-Cr | [16] |
| Ryugu A0060 | CI-like | dolomite (dominant), breunnerite, calcite | 6.10 | 0.90 | 4566.3 | 0.7 | 0.9 | 1.0 | Carbs with imp-Cr | [16] |
| Ryugu A0022, A0033, C0008 | CI-like | dolomite | 4.14 | 0.68 | 4564.2 | 0.8 | 1.0 | 3.1 | Syn calcite | [17] |

SC denotes San Carlos, Syn denotes synthetic, Carbs denotes carbonates, imp-Cr denotes implanted chromium

*Data are not shown in Figure S9.1.

[1] de Leuw S, Rubin AE, Schmitt AK et al (2009) ^53^Mn-^53^Cr systematics of carbonates in CM chondrites: Implications for the timing and duration of aqueous alteration. Geochim Cosmochim Acta 73:7433–7442.

[2] Fujiya W, Sugiura N, Hotta H et al (2012) Evidence for the late formation of hydrous asteroids from young meteoritic carbonates. Nature Comms 3:627.

[3] Lee MR, Lindgren P, Sofe MR et al (2012) Extended chronologies of aqueous alteration in the CM2 carbonaceous chondrites: Evidence from carbonates in Queen Alexandra Range 93005. Geochim Cosmochim Acta 92:148–169.

[4] Jilly CE, Huss GR, Krot AN et al (2014) ^53^Mn-^53^Cr dating of aqueously formed carbonates in the CM2 lithology of the Sutter’s Mill carbonaceous chondrite. Meteorit Planet Sci 49:2104–2117.

[5] Visser R, John T, Whitehouse MJ et al (2020) A short-lived ^26^Al induced hydrothermal alteration event in the outer solar system: Constraints from Mn/Cr ages of carbonates, Earth Planet Sci Lett 547:116440.

[6] Fujiya W et al (2022) Hydrothermal activities on C-complex asteroids induced by radioactivity. Astrophys J Lett 924:L16.

[7] Van Kooten EMME, Wielandt D, Schiller M et al (2016) Isotopic evidence for primordial molecular cloud material in metal-rich carbonaceous chondrites. Proc Nat Ac Sci USA 113:2011–2016.

[8] Jilly-Rehak CE, Huss GR, Nagashima K (2017) ^53^Mn–^53^Cr radiometric dating of secondary carbonates in CR chondrites: Timescales for parent body aqueous alteration. Geochim. Cosmochim Acta 201:224–244.

[9] Endress M, Bischoff A (1996) Carbonates in CI chondrites: Clues to parent body evolution. Geochim Cosmochim Acta 60:489–507.

[10] Hoppe P, Macdougall JD Lugmair GW (2007) High spatial resolution ion microprobe measurements refine chronology of carbonate formation in Orgueil. Meteorit Planet Sci 42:1309–1320.

[11] Fujiya W, Sugiura N, Sano Y et al (2013) Mn-Cr ages of dolomites in CI chondrites and the Tagish Lake ungrouped carbonaceous chondrite. Earth Planet Sci Lett 362:130–142

[12] Steele RCJ, Heber VS, McKeegan KD (2017) Matrix effects on the relative sensitivity factors for manganese and chromium during ion microprobe analysis of carbonate: Implications for early Solar System chronology. Geochim Cosmochim Acta 201:245–259.

[13] Yokoyama T, Nagashima K, Nakai I et al (2023) Samples returned from the asteroid Ryugu are similar to Ivuna-type carbonaceous meteorites. Science 379:eabn7850.

[14] Petitat M, Marrocchi Y, McKeegan KD et al (2011) ^53^Mn-^53^Cr ages of Kaidun carbonates. Meteorit Planet Sci 46:275–283.

[15] Bischoff A, Alexander CMO’D, Barrat J-A et al (2021) The old, unique C1 chondrite Flensburg – Insight into the first processes of aqueous alteration, brecciation, and the diversity of water-bearing parent bodies and lithologies. Geochim Cosmochim Acta 293:142–186.

[16] McCain, K.A., Matsuda, N., Liu, M.C et al (2023) Early fluid activity on Ryugu inferred by isotopic analyses of carbonates and magnetite. Nature Astro 7:309–317.

[17] Nakamura E, Kobayashi K, Tanaka R et al (2022) On the origin and evolution of the asteroid Ryugu: A comprehensive geochemical perspective. Proc Jpn Acad Ser B 98:227–282.
